# International Society for Diseases of the Esophagus consensus on the diagnosis and treatment of anastomotic leak after esophagectomy

**DOI:** 10.1093/dote/doag006

**Published:** 2026-02-05

**Authors:** Sanne K Stuart, Jobbe M G Lemmens, Grard A P Nieuwenhuijzen, Richard P T Evans, Sivesh K Kamarajah, Ian Y H Wong, Bas P L Wijnhoven, Ewen A Griffiths, Bastiaan R Klarenbeek, Sander Ubels, Camiel Rosman, ISDE Guideline AL Collaborative Group, James Bond, Magnus Nilsson, Riccardo Rosati, Robert E Merritt, B Mark Smithers, Simon Law, Peter P Grimminger, Seong Yong Park, George B Hanna, Michal Hubka, Yaxing Shen, Wietse J Eshuis, Roos E Pouw, Elke van Daele, Carmen L Mueller, John V Reynolds, Ivo Boškoski, Stefan Seewald, Arnaud Lemmers, Hitoshi Fujiwara

**Affiliations:** Department of Surgery, Radboud Institute of Health Sciences, Radboud University Medical Center, Nijmegen, The Netherlands; Department of Surgery, Radboud Institute of Health Sciences, Radboud University Medical Center, Nijmegen, The Netherlands; Department of Surgery, Catharina Hospital, Eindhoven, The Netherlands; Department of Upper Gastrointestinal Surgery, University Hospitals Birmingham NHS Foundation Trust, Queen Elizabeth Hospital Birmingham, Birmingham, UK; Institute of Immunology and Immunotherapy, College of Medical and Dental Sciences, University of Birmingham, Birmingham, UK; Department of Upper Gastrointestinal Surgery, University Hospitals Birmingham NHS Foundation Trust, Queen Elizabeth Hospital Birmingham, Birmingham, UK; NIHR Doctoral Fellow, Department of Applied Health Sciences, School of Health Sciences, University of Birmingham, Birmingham, UK; Department of Surgery, The University of Hong Kong, Queen Mary Hospital, Hong Kong, China; Erasmus MC Cancer Institute, Erasmus University Medical Center, Department of Surgery, Rotterdam, The Netherlands; Department of Upper Gastrointestinal Surgery, University Hospitals Birmingham NHS Foundation Trust, Queen Elizabeth Hospital Birmingham, Birmingham, UK; Institute of Immunology and Immunotherapy, College of Medical and Dental Sciences, University of Birmingham, Birmingham, UK; Department of Surgery, Radboud Institute of Health Sciences, Radboud University Medical Center, Nijmegen, The Netherlands; Department of Surgery, Radboud Institute of Health Sciences, Radboud University Medical Center, Nijmegen, The Netherlands; Department of Surgery, Canisius-Wilhelmina Hospital, Nijmegen, The Netherlands; Department of Surgery, Radboud Institute of Health Sciences, Radboud University Medical Center, Nijmegen, The Netherlands

**Keywords:** algorithms, anastomotic leak, consensus, esophagectomy

## Abstract

Background: Anastomotic leak (AL) is a severe complication after esophagectomy. Guidelines for the management of AL are lacking. This study aimed to develop a consensus statement for managing AL after esophagectomy. A three-stage modified Delphi study was performed in collaboration with the International Society for Diseases of the Esophagus Guidelines Committee. In Stage 1, a scoping systematic review was performed to identify available literature used to formulate Delphi statements. Stage 2 involved a two-round Delphi survey, distributed globally to surgeons and gastroenterologists. Consensus was defined as ≥80% (strong) (dis)agreement on a Delphi statement. During Stage 3 (guideline development), an international expert panel formulated clinical recommendations based on Delphi consensus and assigned strength in line with Grading of Recommendations Assessment, Development, and Evaluation principles. A clinical care algorithm was developed based on these recommendations. Of 5.843 articles screened, 118 were included to form Delphi statements. The Delphi survey was completed by 106 respondents in the first round and 136 in the second. Based on Delphi consensus and expert panel discussions, 12 diagnostic recommendations were formulated, covering clinical signs, biochemical tests, and imaging strategies. 11 recommendations were formulated regarding treatment strategies, including indications and techniques for supportive care, drainage and defect closure. This led to the development of a clinical care algorithm. A consensus statement for the diagnosis and treatment of AL after esophagectomy was developed. This may aid clinicians in the diagnosis and management of AL and provide a tool for standardizing clinical practice with the aim to improve patient outcomes.

## INTRODUCTION

Anastomotic leak (AL) is a severe complication after esophagectomy, occurring in 10%–20% of patients.^1,2^ AL is associated with substantial postoperative morbidity, mortality, reinterventions, prolonged hospital stay, and reduced quality of life.^2–6^ Diagnosis and treatment of AL is challenging, with large variation between centers likely driven by a lack of evidence from well-designed clinical trials, institutional experience, and the availability of diagnostic and interventional modalities.^7–9^ Since this is an evolving field with new modalities, there is a need for vivid guidelines and standardization, as a generally accepted approach for the diagnosis and treatment of AL is lacking, which may contribute to the interhospital and global variations in failure to rescue.^10–12^

Early diagnosis of AL is felt to be important to enable prompt treatment, prevent clinical deterioration, and thereby reduce failure to rescue.^12,13^ AL can be suspected based on various clinical and biochemical findings, usually followed by imaging or endoscopy to confirm the diagnosis, but a generally accepted algorithm is lacking.^10,14–16^ Various endoscopic, radiological and surgical treatment strategies have been reported,^17,18^ but robust scientific evidence supporting a specific strategy or technique is lacking.^18–20^

Therefore, a consensus statement could provide a base for guiding clinical practice.^21^ International participation may provide recommendations for clinical care to guide clinicians in the management of AL, promote standardization of care, and reduce practice variation, which may improve patient outcomes.^22^

This study aimed to develop an international consensus statement on the diagnosis and treatment of AL after esophagectomy for cancer and to develop a clinical care algorithm for the management of AL.

## METHODS

**Fig. 1:**
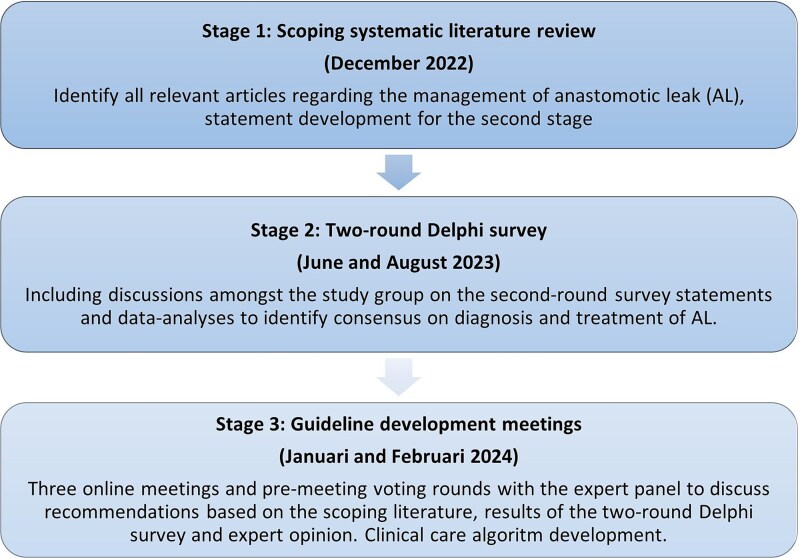
Overview of the modified Delphi process to develop a consensus statement for the diagnosis and treatment of AL after esophagectomy.

A modified Delphi study was conducted in collaboration with the Guidelines Committee of the International Society for Diseases of the Esophagus (ISDE), Oesophago-Gastric Anastomosis Audit (OGAA) initiative, and Treatment of AL after Esophagectomy (TENTACLE—Esophagus) study group. The modified Delphi study comprised three stages: (i) scoping systematic literature review, (ii) two-round Delphi survey, and (iii) guideline development meetings (Fig. 1). The Delphi method is a reliable process for developing consensus for a clinical problem, particularly when the supporting evidence base is scarce.^23^ The modified Delphi methodology has been used previously.^24^ This study did not require institutional review board approval under Dutch law, as it did not involve confidential patient data and no intervention.

Stage i: Scoping systematic literature review.

A scoping systematic literature review was performed using PubMed, Embase and the Cochrane Library to identify relevant articles on diagnosis and treatment of AL after esophagectomy published before January 2023. The search terms used were ‘anastomotic leak*’ and ‘esophagectom*,’ ‘oesophagectom*,’ ‘esophagogastrectom*,’ ‘oesophagogastrectom*,’ or ‘esophagus resection.’ Inclusion criteria were: (i) studies reporting on diagnosis and/or treatment of AL in patients after curative-intent esophagectomy with gastric tube reconstruction for esophageal or gastro-esophageal junction carcinoma; (ii) free full-text availability; (iii) published in the English language. Exclusion criteria were as follows: (i) studies including children (< 18 years of age) or pre-clinical or animal studies; (ii) conference abstracts or videos; (iii) and case reports or case series including <5 patients with AL. After excluding duplicates, two researchers (SS, JL) independently reviewed the identified articles, with discrepancies discussed and resolved by a third researcher (SU). The included studies were grouped by study domain: clinical signs, biochemical signs, imaging modalities, and the different treatment modalities. All included cohort and case–control studies were assessed for methodological quality using the Newcastle-Ottawa scale, which evaluates the risk of bias based on predefined criteria.^25^

Stage ii: Two-round Delphi survey.

A Delphi survey was developed by the study team based on the literature found in Stage 1. Delphi statements were formulated based on the findings, and conclusions of studies included in the review. Statements were discussed with the study team before being included in the Delphi survey and delivered through an online survey platform (www.surveymonkey.com). The Delphi survey was distributed to members of the ISDE, societies associated with the ISDE and via the study networks of OGAA and TENTACLE—Esophagus by email and social media accounts. Surgeons, gastroenterologists and (interventional) radiologists involved in the diagnosis and treatment of patients with AL were invited to complete the Delphi survey. Responses from trainees were excluded. Respondents’ details such as institution type, annual hospital esophagectomy volume (defined as low [<20], middle [20–59], and high [≥60]),^26^ preferred surgical technique, and geographical location were recorded.

Respondents were asked to score each Delphi statement using a 6-point Likert scale ranging from 1 (strongly disagree) to 6 (strongly agree), with the option to abstain from voting if a particular statement was outside of their area of expertise. During the first round, respondents had the opportunity to propose modifications or suggest additional statements, which were then considered by the study team for inclusion in the second Delphi survey round. Statements that reached consensus in the first round were presented, but omitted from voting during the second round.

Responses were analyzed anonymously. Only complete responses were used for analysis and duplicate responses from the same respondent were excluded. Consensus was reached when ≥80% of respondents (strongly) (dis)agreed and near consensus was reached when 70%–80% (strongly) (dis)agreed.^24^ Cronbach’s α was used to evaluate the internal consistency of each Delphi survey and an α of at least 0.80 was deemed acceptable.^24,27^

Stage iii: Guideline development meetings.

During this final stage, three guideline development meetings were conducted to formulate clinical recommendations and a clinical algorithm to guide diagnosis and treatment of AL. An international expert panel of 20 esophageal surgeons and gastroenterologists was formed, based on their expertise in management of AL, experience in high-volume centers, and/or substantial scientific contributions on esophageal surgery. Potential panel members who met these criteria were identified through their active role within the ISDE, involvement in major international studies, or recognized scientific contributions to the field. Particular attention was paid to achieving the broadest possible geographic representation. All panel members are listed in the ‘ISDE Guideline AL Collaborative Group’.

Recommendations were formulated by the expert panel in line with Grading of Recommendations Assessment, Development, and Evaluation principles.^28^ Recommendations were based on the literature review and statements that reached (near) consensus during the Delphi survey rounds. Three meetings were organized with the expert panel using videoconferencing, and were led by the co-chairs of the ISDE Guidelines Committee (EG, BW).

Before each meeting, the panel members were provided with draft recommendations, including results of the first two rounds, and were asked to vote on the strength of the recommendations. These draft recommendations, including proposed strength, were then discussed during the meetings, and any textual and content-related adjustments were made based on the discussions. During the meetings, the findings of the literature review and Delphi survey were summarized per recommendation. For each recommendation, the level of evidence and strength of recommendation were assigned using a grading system adapted from European Society of Cardiology (ESC) (Tables 1 and 2).^29^ The level of evidence was assigned based on the literature review. The strength of recommendation was assigned during the meeting by the expert panel, considering the level of evidence and their expert opinion, while taking into account the international Delphi consensus, as well as the assumed benefits, costs, availability, and associated risks and burdens.

**Table 1:** Levels of evidence adapted from the ESC evidence grading system^29^

**Level of evidence**	**Definition**
A	Data derived from multiple randomized clinical trials or meta analyses
B	Data derived from a single randomized clinical trial or large non-randomized studies
C	Consensus of opinion of the experts and/or small studies, retrospective studies, registries.

**Table 2:** Strength of recommendations adapted from the ESC evidence grading system^29^

**Strength**	**Definition**
I	Evidence and/or general agreement that a given treatment or procedure is beneficial, useful, effective.
II	Conflicting evidence and/or divergence of opinion about the usefulness/efficacy about the given treatment or procedure.
IIa	Weight of evidence/opinion is in favor of usefulness/efficacy.
IIb	Usefulness/efficacy is less well established by evidence/opinion.
III	Evidence or general agreement that the given treatment or procedure is not useful/effective and in some cases may be harmful.

After having formulated recommendations on diagnosis and treatment of AL during the first two meetings, a draft clinical care algorithm was developed by the study team. During the final meeting, the clinical care algorithm and corresponding recommendations were discussed and appropriate adjustments were made based on the discussion in the expert panel.

### Data collection and analysis

Frequencies and corresponding percentages were used for dichotomous data, while continuous data was presented as mean with standard deviation (SD) or median with interquartile range (IQR) where appropriate. All analyses were performed using SPSS version 29.0 (IBM Corporation, Armonk, NY).

## RESULTS

Stage i: Scoping systematic literature review.

In total, 5.843 articles were identified, of which 118 articles were included after full-text review (Supplementary Fig. S1), with no exclusions based on full-text unavailability. Most studies were (retrospective) cohort studies or review articles, and no randomized controlled trials were available (Supplementary Table S1). Of these 118 articles, 54 articles (45.8%) addressed the diagnosis of AL, 48 (40.7%) treatment, and 16 (13.6%) covered both. Statements were formulated based on this available literature, with 59 statements covering diagnostic strategies and 53 addressing treatment strategies for AL.

Stage ii: Two-round Delphi survey.

A total of 106 respondents from 35 countries across 6 continents completed the survey during the first round. Most respondents were from Europe (63.2%), were surgeons (98.1%), and worked in middle-volume hospitals (49.1%) (Supplementary Table S2). Of 112 formulated statements, 17 (15.2%) reached consensus and 19 (17.0%) reached near consensus during the first round (Table 3, Supplementary Table S3). A total of 824 free-text comments from the first round survey were evaluated and used to revise existing statements and create new ones, resulting in 125 statements for voting in the second round, which was completed by 136 respondents from 39 countries (Supplementary Table S2). Of the respondents who completed the first round survey, 64 respondents (60.4%) also completed the second round. During the second round, an additional 18 (14.4%) statements reached consensus (including 6 statements that reached near consensus during the first round), and 14 (11.2%) additional statements reached near consensus (Table 3, Supplementary Table S4). The Cronbach’s α for the first Delphi survey was 0.94 and 0.96 for the second round, indicating good internal reliability. Consensus based on geographical location and hospital volume is available in the supplementary file (Tables S5 and S6).

**Table 3:** Consolidation of the statements that reached consensus or near consensus during the two-round Delphi survey

**Statements**	**Consensus^†^ (%)**
**Consensus**	
***Clinical signs***	
Fever is a useful clinical sign in the diagnostic process of AL after esophagectomy.	85
Respiratory distress is a useful clinical sign in the diagnostic process of AL after esophagectomy.	81
Changed aspect of postoperative drain fluids is a useful clinical sign in the diagnostic process of AL after esophagectomy.	92
Tachycardia, among other signs and biochemical results, is a useful clinical sign in the diagnostic process of AL after esophagectomy.	84
Redness and swelling in the associated neck incision (cervical anastomosis), among other signs and biochemical results, is a useful clinical sign in the diagnostic process of AL after esophagectomy.	87
***Biochemical signs***	
Serum CRP is a useful biochemical test in the diagnostic process of AL after esophagectomy.	90
Monitoring serum CRP on POD 5 is useful in the diagnostic process of AL.	86
The trend in CRP values is useful in the decision for additional diagnostics regarding AL.	89
Serum WBC is a useful biochemical test in the diagnostic process of AL after esophagectomy.	83
***Diagnostic modalities***	
Routine endoscopy in asymptomatic patients and normal biochemical results should not be performed.	84
CT-scan is the next diagnostic assessment if you suspect cervical AL based on clinical sign(s) and/or biochemical test(s) without local signs of infection.	80
It is useful to perform a CT-scan if you confirmed cervical AL by opening the cervical incision and the patient is septic.	89
CT-scan is the first choice diagnostic assessment if you suspect intrathoracic AL based on clinical sign(s) and/or biochemical test(s).	84
When AL is diagnosed on endoscopy, it is useful to perform an additional CT scan.	90
The size of the defect should be measured during endoscopy in order to guide AL treatment.	85
The condition of the gastric conduit should be assessed during endoscopy in order to guide AL treatment.	98
The extent of contamination should be assessed on CT in order to guide AL treatment.	99
***Supportive care treatment***	
Broad spectrum antibiotics should be part of non-invasive management/supportive care of all AL patients.	99
Feeding support should be part of non-invasive management/supportive care of all AL patients.	98
Nil-by-mouth should be part of non-invasive management/supportive care of all AL patients.	84
***Drainage interventions***	
Additional drainage interventions should be performed in patients with a cervical AL with mediastinal fluid collections.	91
Additional drainage interventions should be performed in patients with a cervical AL with pleural fluid collections.	92
Bedside opening of the cervical incision is a useful drainage tool in patients with cervical AL.	81
Additional drainage interventions should be performed in patients with an intrathoracic leak with mediastinal fluid collections.	92
Additional drainage interventions should be performed in patients with an intrathoracic leak with pleural fluid collections.	96
Radiological drainage tube is a useful drainage tool in patients with an intrathoracic leak.	81
EVT is a useful drainage tool in patients with an intrathoracic leak.	82
When the patient with AL shows signs of uncontrolled sepsis is that reason for surgical reintervention.	85
When the patient with AL shows signs of uncontrolled sepsis, surgical washout and better drainage is indicated.	84
Step-up after failure of initiated treatment of AL is reason for surgical washout and better drainage.	82
***Leak closure interventions***	
Leak closure interventions in addition to supportive care and drainage interventions, should be performed in septic patients with intrathoracic AL with mediastinal fluid collections.	83
Leak closure interventions in addition to supportive care and drainage interventions, should be performed in septic patients with intrathoracic AL with pleural fluid collections.	83
EVT is a useful leak closure intervention in patients with intrathoracic AL.	82
***Conduit ischemia/necrosis***	
Continuity-preserving treatment should be the first choice treatment for patients with AL and mild symptoms, and limited ischemia/necrosis of the top.	84
Primary esophageal diversion should be the first choice treatment for septic patients with AL and overall ischemia/necrosis of the conduit.	89
**Near consensus**	
***Biochemical signs***	
Drain amylase is a useful biochemical test in the diagnostic process of AL after esophagectomy.	74
Monitoring serum CRP on POD 6 is useful in the diagnostic process of AL.	76
Monitoring serum CRP on POD 7 is useful in the diagnostic process of AL.	72
Monitoring serum CRP should start on POD 3, if routine monitoring is performed.	74
***Diagnostic modalities***	
Routine CT-scan in asymptomatic patients and normal biochemical results should not be performed.	71
When you suspect cervical AL based on clinical signs and/or biochemical tests, bedside opening of the cervical incision is indicated if there are local signs of infection.	74
When you suspect cervical AL based on clinical signs and/or biochemical tests, bedside opening of the cervical incision is indicated if the patient is septic.	76
Bedside opening of the cervical incision is the next diagnostic assessment if you suspect cervical AL based on clinical sign(s) and/or biochemical test(s) with local signs of infection.	72
CT-scan is the next diagnostic assessment if you suspect cervical AL based on clinical sign(s) and/or biochemical test(s) with local signs of infection.	77
CT-scan and endoscopy is the first choice diagnostic assessment if you suspect intrathoracic AL based on clinical sign(s) and/or biochemical test(s).	71
When AL is diagnosed on CT scan, it is useful to perform an additional endoscopy.	77
***Supportive care treatment***	
Antifungal therapy should be part of non-invasive management/supportive care of all AL patients.	74
PPI should be part of non-invasive management/supportive care of all AL patients.	79
Nasogastric drainage tube is a useful drainage tool in patients with an intrathoracic leak.	76
***Drainage interventions***	
Surgical drainage tube is a useful drainage tool in patients with an intrathoracic leak.	79
Step-up after failure of initiated treatment of AL is reason for surgical reintervention.	77
Uncontained intrathoracic AL is reason for surgical washout and better drainage.	74
***Leak closure interventions***	
Additional leak closure interventions should be performed in patients with an intrathoracic leak with pleural fluid collections.	74
Leak closure interventions in addition to supportive care and drainage interventions, should be performed in septic patients with cervical AL with mediastinal fluid collections.	70
Endoscopic suturing is not a useful leak closure intervention in patients with cervical AL.	76
Leak closure interventions in addition to supportive care and drainage interventions, should be performed in patients with mild symptoms and intrathoracic AL with pleural fluid collections.	77
Stent placement is a useful leak closure modality in patients with an intrathoracic leak.	71
EVT combined with stent is a useful leak closure modality in patients with an intrathoracic leak.	77
***Conduit ischemia/necrosis***	
Continuity-preserving treatment should not be the first choice treatment for septic patients with AL and overall ischemia/necrosis of the conduit.	75
Primary esophageal diversion should be the first choice treatment for patients with AL and mild symptoms and overall ischemia/necrosis of the conduit.	75

AL, anastomotic leak; POD, postoperative day; CRP, C-reactive protein; WBC, white blood count; CT, computed tomography; EVT, endoscopic vacuum therapy; Consensus = ≥80% of respondents (strongly) (dis)agreed; near consensus = 70–80% (strongly) (dis)agreed. ^†^This table shows the highest result achieved in both rounds*.*

**Table 4:** Recommendations regarding the diagnosis of AL after esophagectomy

**Recommendations**	**Level** ^**†**^	**Strength** ^**‡**^
**Clinical signs**		
1A. Changed aspect of postoperative drain fluids is suggestive for an AL.	C	I
1B. Any of the following clinical signs can be taken into account for the diagnosis of an AL: fever, respiratory distress, tachycardia.	C	IIa
2. Redness and swelling of the neck incision in patients with a cervical anastomosis are suggestive for an AL.	C	I
**Biochemical signs**		
3. Serum CRP can be useful in the diagnostic process for AL, and monitoring the trend from postoperative day 3 to 7 after esophagectomy can be considered.	C	IIa
4. If drains are placed during esophagectomy, drain amylase levels might be useful in the diagnostic process for AL.	C	IIb
**Diagnostic modalities**		
5. For patients with a suspected intrathoracic AL, CT with oral contrast should be performed as the first diagnostic modality.	C	I
6. For patients with suspected cervical leak without local signs of infection, a CT with oral contrast can be performed as a diagnostic of first choice.	C	IIa
7. For patients with suspected cervical leak with local signs of infection, CT with oral contrast or bedside opening of the incision can be used as a diagnostic of first choice.	C	IIa
8. For septic patients with an AL confirmed by bedside opening of the cervical incision, an additional CT scan should be performed.	C	I
9. If AL is confirmed by endoscopy, an additional CT scan with oral contrast should be performed to assess the extent of contamination.	C	I
10. If AL is confirmed by CT scan, an additional endoscopy can be performed to assess the size of the defect and condition of the gastric conduit.	C	IIa
11. For patients with a persistent suspicion of AL, despite a negative or inconclusive CT scan, an additional endoscopy should be performed.	C	I
12. Routine screening for AL in asymptomatic postoperative patients is not considered useful.	C	III

^†^Level of evidence

^‡^Strength of recommendation.

Stage iii: Guideline development meetings.

**Fig. 2:**
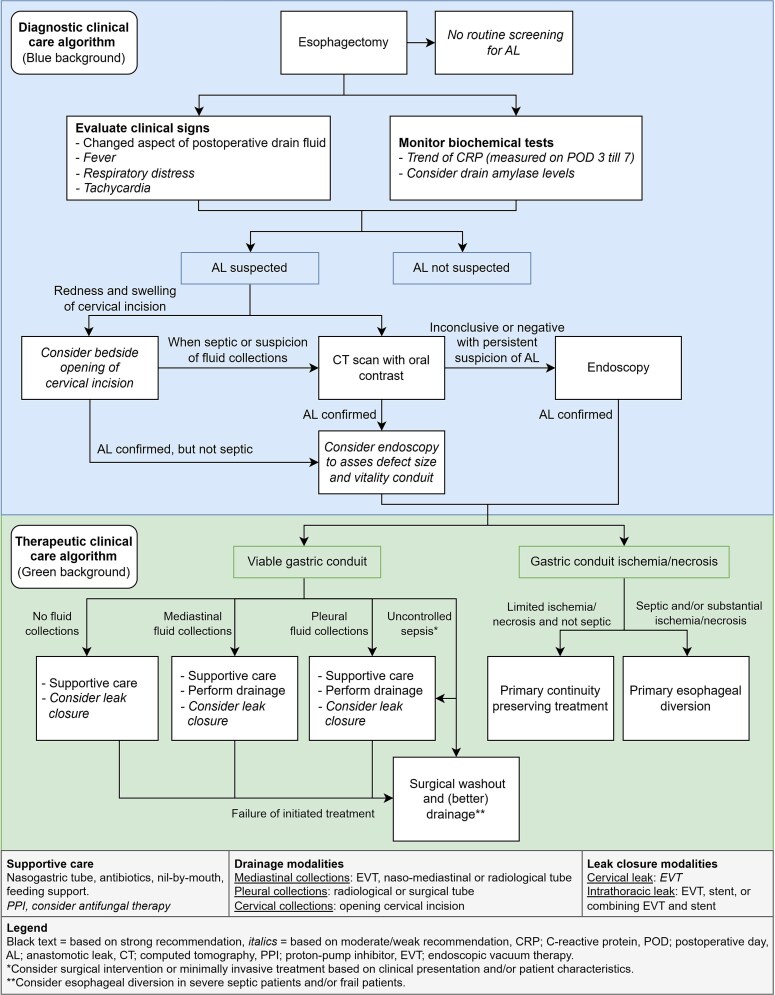
Clinical care algorithm on the diagnosis and treatment of AL after esophagectomy.

During the three guideline meetings, the expert panel formulated 12 recommendations regarding diagnosis of AL after esophagectomy and 11 recommendations regarding treatment, each based on (a combination of) the 56 Delphi statements that reached (near) consensus. The number of panel members (excluding the study team members) who contributed to the three meetings through pre-meeting voting and/or attendance was 17 (85%), 15 (75%), and 10 (50%), respectively. All recommendations were based on a low level of evidence and a strength was assigned during the development meetings (Tables 4 and 5). See below for an overview of the formulated recommendations. Detailed descriptions and additional insights from the expert panel are available in the supplementary file (Supplementary file Stage 3). An algorithm for clinical practice was developed based on the formulated recommendations, discussed by the expert panel, and consensus was reached (Fig.  2). The algorithm is intended for use regardless of surgical approach, as clinical signs, biochemical tests, and treatment principles are similar. For cervical anastomosis, cervical wound inflammation raises suspicion of AL, and may indicate cervical wound opening. For treatment, variations in leak closure modalities based on anastomotic location are addressed in the figure legend.

**Table 5:** Recommendations regarding the treatment of AL after esophagectomy

**Recommendations**	**Level** ^**†**^	**Strength** ^**‡**^
**Supportive care treatment**		
13A. The initial supportive care treatment for AL, should include broad-spectrum antibiotics, feeding support, and nil-by-mouth.	C	I
13B. PPI can be used or continued as supportive care treatment for AL.	C	IIa
13C. Antifungal therapy may be considered as supportive care treatment for AL.	C	IIb
14. Nasogastric tube drainage, for drainage and decompression of the gastric conduit, should be considered during the initial treatment of all leak patients.	C	I
15. For patients with a confirmed leak without fluid collections, and only mild clinical signs, non-invasive management with supportive care may be considered as primary treatment.	C	IIb
**Drainage interventions**		
16. For leak patients with mediastinal and/or pleural fluid collections, drainage should be performed in addition to supportive care.	C	I
17. Drainage modalities to consider for mediastinal fluid collections are EVT, naso-mediastinal tube drainage, and/or radiological tube drainage. For pleural fluid collections, radiological tube drainage and/or surgical tube drainage should be considered. For a cervical leak, bedside opening of the cervical incision should be considered as a drainage modality.	C	I
18. For leak patients with signs of uncontrolled sepsis and/or failure of primary treatment, surgical washout and drainage should be performed.	C	I
**Leak closure interventions**		
19. For patients with an intrathoracic or cervical leak with mediastinal and/or pleural fluid collections, endoscopic leak closure interventions can be performed in addition to drainage and supportive care.	C	IIa
20. If a leak closure intervention is performed in patients with an intrathoracic leak, EVT, stent, and/or EVT combined with stent should be considered as useful closure modalities.	C	I
21. If surgical washout and drainage is performed in patients, additional surgical closure of the defect using sutures or tissue may be considered.	C	IIb
**Conduit ischemia/necrosis**		
22. For non-septic leak patients with limited ischemia/necrosis of the top of the gastric conduit, continuity-preserving treatment should be considered as primary treatment.	C	I
23. For leak patients with substantial ischemia/necrosis of the gastric conduit, primary esophageal diversion should be performed.	C	I

^†^Level of evidence; ^‡^Strength of recommendation.

### Recommendations regarding diagnosis of AL

Clinical signs are essential for making a judgment on the likelihood of an AL. Consensus was reached that changes in aspect of drain fluid (if a drain is in situ), and redness and/or swelling of the cervical incision are suggestive of AL. Although not specific to AL, fever, respiratory distress, and tachycardia can also be considered signs of AL.

Biochemical abnormalities indicative of AL are better described in the literature, with C-reactive protein (CRP) being the most frequently used marker. Serial CRP measurements can be used to monitor the trend, mostly useful as a negative predictor, since elevation can also reflect the surgical trauma or other infectious complications. There was no consensus supporting a specific cut-off of CRP prompting further diagnostic imaging. If drains are placed during esophagectomy, drain amylase might also be useful. However, a low level does not exclude AL and can be elevated early in the postoperative course due to leakage of gastric contents during the creation of the anastomosis.

Modalities to diagnose AL, as identified by literature and consensus, are CT with oral contrast, bedside opening of the cervical incision, and endoscopy. CT with oral contrast is the preferred diagnostic modality when AL is suspected, as it provides immediate information on the extent of the leakage, including the degree of mediastinal and/or pleural contamination, and may also diagnose other complications. When a cervical leak is suspected based on local signs of infection, bedside opening of the cervical incision can be considered as the first choice, but CT should be performed as well in septic patients to diagnose pleural or mediastinal involvement. An additional endoscopy can be performed to assess the size of the defect and the perfusion of the gastric conduit, or if the CT scan is negative or inconclusive. Routine screening for AL in asymptomatic patients is not considered useful.

### Recommendations regarding treatment of AL

Initial supportive care treatment for AL should include broad-spectrum antibiotics, enteral feeding, and nil-by-mouth. Proton-pump inhibitors (PPI) can be considered, and antifungal therapy may also be used, with initiation postponed until a later time period, guided by cultures and supervised by a medical microbiologist specialist. Additionally, nasogastric tube drainage should be considered to decompress the gastric conduit and limit further contamination.

Drainage interventions should be performed in AL patients with mediastinal and/or pleural fluid collections. Various drainage modalities are available, including endoscopic vacuum therapy (EVT), naso-mediastinal tube drainage, surgical tube drainage, radiological tube drainage and/or bedside opening of the cervical incision. There was no literature or consensus to determine a preferred drainage method. A modality may be chosen based on location of fluid collection, local availability and expertise. Surgical washout and drainage should be considered in cases of uncontrolled sepsis and/or failure of primary treatment.

Leak closure interventions can be considered in addition to supportive care and drainage interventions to provide better source control and enable oral feeding. Modalities to consider include EVT, stent, and/or a combination of both.

Substantial conduit ischemia/necrosis in patients with AL should be treated with primary esophageal diversion. However, in patients with limited ischemia/necrosis of the top of the gastric conduit who have no signs of severe sepsis, continuity-preserving treatment should be considered with close and careful monitoring. This includes (combinations of) surgical or endoscopic drainage and/or defect closure, without performing esophageal diversion.

## DISCUSSION

This study aimed to develop an international clinical consensus statement and algorithm for physicians involved in the care of patients undergoing esophagectomy. While previous studies have also suggested algorithms for managing AL, this study is the first to integrate a systematic literature review with international consensus supported by a multidisciplinary expert panel and represent the best available scientific evidence so far.^10,14,15,30–32^

Diagnosis of AL is challenging due to variation in clinical presentation and low accuracy of diagnostic tests.^15^ The scoping systematic literature review revealed only two studies on clinical signs of AL.^33,34^ Nonetheless, the Delphi process allowed for the development of consensus-based recommendations. Biochemical tests, specifically CRP,^35–46^ white blood count (WBC),^38,41,47^ and drain amylase,^36,39,45,48–53^ have been frequently assessed. CRP showed the highest accuracy among the three, which is supported by its broad clinical use as indicated by the Delphi consensus (90%). Although WBC also reached consensus in the second Delphi round, it was not included in the recommendations by the expert panel, due to its lower diagnostic accuracy and minimal additional benefit compared to CRP.^54^ Also not recommended are routine oral contrast studies before starting oral intake, as a meta-analysis of small cohort studies found that routine use lacks adequate sensitivity and positive predictive value to be considered an effective screening tool.^55^ Instead, CT scan with oral contrast is preferred as the first choice diagnostic modality to diagnose AL. CT has a higher sensitivity, can show the extent of contamination, has the potential to diagnose other complications, and is widely available in centers that perform esophagectomy worldwide.^15,56^ CT-scoring systems have recently been developed, and may increase diagnostic accuracy even further.^57,58^ Despite the fact that there is currently no gold standard diagnostic tool for AL, as a result of our joint effort a clinical diagnostic guideline was developed by combining clinical signs, biochemical tests, and (imaging) modalities, to improve early detection of AL.

The treatment of AL may be even more complex, with a larger variety of treatment options.^18^ Ubels *et al*.^7^ categorized treatment modalities into four main principles based on a mixed-methods study including expert discussions: supportive care; drainage; defect closure; and esophageal diversion. It is recommended that the initial treatment for all patients with AL includes supportive care. This should at least include feeding support, antibiotics, nil-by-mouth, and nasogastric drainage, based on Delphi consensus, expert discussions, and the association shown in literature between postoperative enteral nutrition and a higher success rate of primary AL treatment.^2^ The second treatment principle, drainage, reached consensus during the first Delphi survey round, recommending the use of drainage interventions in case of pleural and/or mediastinal fluid collections. Drainage was identified by the expert panel as the most important aspect of AL treatment. However, defining which drainage modality to use proved more challenging. The general consensus from expert discussions was to use the modality with the most expertise of the treating center. In the past decade, there has been a growing interest in defect closure, particularly using endoscopic modalities like stents, EVT and even recently a combination of the two. Recent small cohort studies show a trend of favoring EVT over stents, due to a lower complication and mortality rate, with the lack of support from randomized data due to the novelty of these procedures.^59–61^ Only consensus was reached for attempting endoscopic leak closure interventions for septic patients with thoracic AL, but consensus was lacking for other clinical presentations. The expert discussions showed substantial variation among experts on the use of leak closure interventions and their indications, demonstrating the knowledge gap regarding the indications, benefits, timing, and selection of the leak closure interventions. Additionally, the availability of these interventions must be considered. Access to endoscopic stenting was available in over 92% of Delphi respondents, while this was only 79% for EVT and 51% for EVT combined with stent, as shown in Supplementary Table S2. However, this does show an increase in availability compared to an international survey from 2021, where EVT availability was 64%.^7^

Based on the recommendations, a diagnostic and therapeutic clinical care algorithm was developed. This algorithm may support physicians in clinical decision-making and could lead to better standardization of clinical practice. Furthermore, national guidelines and institutional protocols can be developed or adjusted based on the recommendations from this study. By following a standardized postoperative protocol focused on the early detection of AL, timely treatment can be ensured. This may lead to better outcomes by preventing patient deterioration, which in turn reduces organ failure, intensive care readmissions, and mortality.^22^ A deliberate choice was made to focus on treatment principles rather than specific modalities, due to the large variation, relatively new developments, and limited evidence supporting individual modalities. This also has the advantage that the recommendations can be applied more universally, even in centers with more limited resources, while also allowing for consideration of local experience. However, careful consideration prior to implementation is needed because the algorithm is mostly consensus-based due to limited evidence. Clinicians may exercise caution while using the clinical care algorithm and deviate from it if felt necessary at their discretion, for example when more evidence becomes available in the future.

A limitation of this study is the limited input from gastroenterologists and (interventional) radiologists in the Delphi survey rounds, as in some countries they are involved in the endoscopic diagnosis and/or treatment of AL, guided by the surgeon. Furthermore, there was an uneven geographical distribution of respondents, with an overrepresentation of participants from Europe and underrepresentation from low-income countries. Hence, input from physicians who may face limited resources is also limited, which could affect the applicability of the recommendations. It remains uncertain whether potential respondents from low-income countries could not be reached through the well-established networks used or if they were scarcely present due to low incidence of esophageal carcinoma.^62^ The latter could suggest that low incidence countries might benefit from the experience of overrepresented countries with more experience in esophagectomies and their complications, by implementing this clinical care algorithm. The algorithm remains applicable even in setting without access to novel (endoscopic) modalities such as EVT, by using alternative drainage techniques like mediastinal drain placement through the defect. There was also an underrepresentation of respondents from Asia and North America. To still achieve a universally applicable and multidisciplinary consensus guideline, 4 gastroenterologists and 16 surgeons from 14 different countries and 4 continents participated in the expert panel.

The previous Esophagectomy Complications Consensus Group (ECCG) established a standardized definition for AL in 2015, our work builds upon this foundation by addressing clinical decision-making and management strategies.^63^ However, the development of robust recommendations was hindered by the lack of reliable studies on the various diagnostic and (endoscopic) treatment modalities, with no available data on long-term or patient-reported outcomes. This highlights the need for research comparing these different techniques, which can facilitate future guideline improvements. Additionally, research should not only focus on evaluating individual modalities but also explore whether implementing a clinical care algorithm that integrates various clinical, biochemical, and techniques can mitigate the severity of AL outcomes in clinical practice. The RESCUE trial, a Dutch nationwide stepped-wedge trial led by our research team, aims to evaluate the effects of implementing a clinical care algorithm after esophagectomy in clinical practice.^64^ While it may not reduce the incidence of AL, early detection could enable timely treatment, resulting in less severe consequences of AL.

## CONCLUSION

This study has developed a consensus statement for the diagnosis and treatment of AL after esophagectomy. The developed recommendations and clinical care algorithm may guide clinicians in the diagnosis and management of AL and provide a tool for standardizing clinical practice with the aim to improve patient outcomes. Future research should focus on evaluating the algorithm’s impact on improving clinical outcomes and compare individual modalities.

## Collaborators

ISDE Guideline AL Collaborative Group.

James Bond (Surrey Memorial Hospital, Surrey, Canada); Magnus Nilsson (Karolinska University Hospital, Stockholm, Sweden); Riccardo Rosati (Vita-Salute San Raffaele University, Milan, Italy); Robert E. Merritt (Ohio State University Wexner Medical Center, Ohio, United States); B Mark Smithers (Princess Alexandra Hospital, University of Queensland, Brisbane, Australia); Simon Law (Queen Mary Hospital, The University of Hong Kong, Hong Kong Special Administrative Region, China); Peter P. Grimminger (University Medical Center Mainz, Mainz, Germany); Seong Yong Park (Samsung Medical Center, Sungkyunkwan University School of Medicine, Seoul, Republic of Korea); George B. Hanna (Imperial College London, London, United Kingdom); Michal Hubka (Virginia Mason Medical Center, Seattle, United States); Yaxing Shen (Zhongshan Hospital, Fudan University, Shanghai, China); Wietse J. Eshuis, Roos E. Pouw (Amsterdam University Medical Center, Amsterdam, The Netherlands); Elke van Daele (Ghent University Hospital, Ghent, Belgium); Carmen L. Mueller (McGill University Health Center, Montreal General Hospital, Montreal, Canada); John V. Reynolds (St James’s Hospital Trinity College, Dublin, Ireland); Ivo Boškoski (Fondazione Policlinico Universitario Agostino Gemelli IRCCS, Rome, Italy); Stefan Seewald (Klinik Hirslanden, Zurich, Switzerland); Arnaud Lemmers (CUB Erasme Hospital, Université Libre de Bruxelles, Brussels, Belgium); Hitoshi Fujiwara (Kyoto Prefectural University of Medicine, Kyoto, Japan).

## Supplementary Material

doag006_Supplementary_file_ISDE_Consensus_Statement_Management_of_AL

## Notes

Specific author contributions: Sanne K. Stuart (Conceptualization, Methodology, Validation, Formal analysis, Investigation, Writing—Original Draft, Writing—Review & Editing, Visualization, Project administration), Jobbe M. G. Lemmens (Conceptualization, Methodology, Validation, Formal analysis, Writing—Original Draft, Writing—Review & Editing), Grard A. P. Nieuwenhuijzen, Richard P. T. Evans, Sivesh K. Kamarajah, Ian Y. H. Wong (Conceptualization, Methodology, Writing—Review & Editing), Bas P. L. Wijnhoven, Ewen A. Griffiths (Conceptualization, Methodology, Writing—review & editing, Supervision), Sander Ubels, Bastiaan R. Klarenbeek, Camiel Rosman (Conceptualization, Methodology, Writing—review & editing, Supervision, Project administration).

Financial support: No funding for current study.

## References

[1] Oesophago-Gastric Anastomosis Study Group . Comparison of short-term outcomes from the international Oesophago-gastric anastomosis audit (OGAA), the esophagectomy complications consensus group (ECCG), and the Dutch upper gastrointestinal cancer audit (DUCA). BJS Open 2021; 5(3): zrab010. 10.1093/bjsopen/zrab010 PMC8140199 35179183

[2] Oesophago-gastric anastomosis study group on behalf of the west midlands research collaborative. Rates of anastomotic complications and their management following esophagectomy: results of the Oesophago-gastric anastomosis audit (OGAA). Ann Surg 2022; 275(2): e382–91. 10.1097/SLA.0000000000004649. 33630459

[3] van Workum F, Verstegen M H P, Klarenbeek B R et al. Intrathoracic vs cervical anastomosis after totally or hybrid minimally invasive esophagectomy for Esophageal cancer: a randomized clinical trial. JAMA Surg 2021; 156(7): 601–10. 10.1001/jamasurg.2021.1555. 33978698 PMC8117060

[4] Goense L, Meziani J, Ruurda J P, van Hillegersberg R. Impact of postoperative complications on outcomes after oesophagectomy for cancer. Br J Surg 2019; 106(1): 111–9. 10.1002/bjs.11000. 30370938

[5] Jezerskyte E, van Berge Henegouwen M I, van Laarhoven H W M et al. Postoperative complications and long-term quality of life after multimodality treatment for Esophageal cancer: an analysis of the prospective observational cohort study of Esophageal-gastric cancer patients (POCOP). Ann Surg Oncol 2021; 28(12): 7259–76. 10.1245/s10434-021-10144-5. 34036429 PMC8519926

[6] Derogar M, Orsini N, Sadr-Azodi O, Lagergren P. Influence of major postoperative complications on health-related quality of life among long-term survivors of esophageal cancer surgery. J Clin Oncol 2012; 30(14): 1615–9. 10.1200/JCO.2011.40.3568. 22473157

[7] Ubels S, Lubbers M, Verstegen M H P et al. Treatment of anastomotic leak after esophagectomy: insights of an international case vignette survey and expert discussions. Dis Esophagus 2022; 35(12): doac020. 10.1093/dote/doac020. PMC9753084 35411928

[8] International variation in surgical practices in units performing Oesophagectomy for oesophageal cancer: a unit survey from the Oesophago-gastric anastomosis audit (OGAA). World J Surg 2019; 43(11): 2874–84. 31332491 10.1007/s00268-019-05080-1

[9] Hagens E R C, Anderegg M C J, van Berge Henegouwen M I, Gisbertz S S. International survey on the Management of Anastomotic Leakage after Esophageal resection. Ann Thorac Surg 2018; 106(6): 1702–8. 10.1016/j.athoracsur.2018.05.009. 29883644

[10] Grimminger P P, Goense L, Gockel I et al. Diagnosis, assessment, and management of surgical complications following esophagectomy. Ann N Y Acad Sci 2018; 1434(1): 254–73. 10.1111/nyas.13920. 29984413

[11] Mortality from esophagectomy for esophageal cancer across low, middle, and high-income countries: an international cohort study. Eur J Surg Oncol 2021; 47(6): 1481–8. 33451919 10.1016/j.ejso.2020.12.006

[12] Moon S W, Kim J J, Cho D G, Park J K. Early detection of complications: anastomotic leakage. J Thorac Dis 2019; 11(Suppl 5): S805–s811. 10.21037/jtd.2018.11.55. 31080662 PMC6503265

[13] Dent B, Griffin S M, Jones R, Wahed S, Immanuel A, Hayes N. Management and outcomes of anastomotic leaks after oesophagectomy. Br J Surg 2016; 103(8): 1033–8. 27146631 10.1002/bjs.10175

[14] Lemmens J, Klarenbeek B, Verstegen M et al. Performance of a consensus-based algorithm for diagnosing anastomotic leak after minimally invasive esophagectomy for esophageal cancer. Dis Esophagus 2023; 36(10): doad016. 10.1093/dote/doad016. PMC10543373 36988007

[15] Barbaro A, Eldredge T A, Shenfine J. Diagnosing anastomotic leak post-esophagectomy: a systematic review. Dis Esophagus 2021; 34(2): doaa076. 10.1093/dote/doaa076. 33565590

[16] Fabbi M, Hagens E R C, van Berge Henegouwen M I, Gisbertz S S. Anastomotic leakage after esophagectomy for esophageal cancer: definitions, diagnostics, and treatment. Dis Esophagus 2021; 34(1): doaa039. 10.1093/dote/doaa039. PMC7801633 32476017

[17] Hua F, Sun D, Zhao X, Song X, Yang W. Update on therapeutic strategy for esophageal anastomotic leak: a systematic literature review. Thorac Cancer 2023; 14(4): 339–47. 36524684 10.1111/1759-7714.14734 PMC9891862

[18] Verstegen M H P, Bouwense S A W, van Workum F et al. Management of intrathoracic and cervical anastomotic leakage after esophagectomy for esophageal cancer: a systematic review. World J Emerg Surg 2019; 14: 17. 30988695 10.1186/s13017-019-0235-4 PMC6449949

[19] Ubels S, Verstegen M H P, Klarenbeek B R et al. Treatment of anastomotic leak after oesophagectomy for oesophageal cancer: large, collaborative, observational TENTACLE cohort study. Br J Surg 2023; 110(7): 852–63. 10.1093/bjs/znad123. 37196149 PMC10364505

[20] Scognamiglio P, Reeh M, Karstens K et al. Endoscopic vacuum therapy versus stenting for postoperative esophago-enteric anastomotic leakage: systematic review and meta-analysis. Endoscopy 2020; 52(8): 632–42. 32316043 10.1055/a-1149-1741

[21] Boulkedid R, Abdoul H, Loustau M, Sibony O, Alberti C. Using and reporting the Delphi method for selecting healthcare quality indicators: a systematic review. PloS One 2011; 6(6): e20476. 10.1371/journal.pone.0020476. 21694759 PMC3111406

[22] Smits F J, Henry A C, Besselink M G et al. Algorithm-based care versus usual care for the early recognition and management of complications after pancreatic resection in the Netherlands: an open-label, nationwide, stepped-wedge cluster-randomised trial. Lancet 2022; 399(10338): 1867–75. 10.1016/S0140-6736(22)00182-9. 35490691

[23] Rosenfeld R M, Nnacheta L C, Corrigan M D. Clinical consensus statement development manual. Otolaryngol Head Neck Surg 2015; 153(2 Suppl): S1–S14. 10.1177/0194599815601394. 26527615

[24] Kamarajah S K, Siddaiah-Subramanya M, Parente A et al. Risk factors, diagnosis and Management of Chyle Leak Following Esophagectomy for cancers: an international consensus statement. Ann Surg Open 2022; 3(3): e192. 10.1097/AS9.0000000000000192. 36199483 PMC9508983

[25] Wells G A, Wells G, Shea B et al. The Newcastle-Ottawa Scale (NOS) for Assessing the Quality of Nonrandomised Studies in Meta-Analyses. Ottawa Hospital Research Institute 2014. Available at: https://ohri.ca/en/who-we-are/core-facilities-and-platforms/ottawa-methods-centre/newcastle-ottawa-scale.

[26] Ubels S, Matthée E, Verstegen M et al. Practice variation in anastomotic leak after esophagectomy: unravelling differences in failure to rescue. Eur J Surg Oncol 2023; 49(5): 974–82. 10.1016/j.ejso.2023.01.010. 36732207

[27] Bland J M, Altman D G. Cronbach's alpha. Bmj 1997; 314(7080): 572. 9055718 10.1136/bmj.314.7080.572 PMC2126061

[28] Guyatt G H, Oxman A D, Vist G E et al. GRADE: an emerging consensus on rating quality of evidence and strength of recommendations. Bmj 2008; 336(7650): 924–6. 10.1136/bmj.39489.470347.AD. 18436948 PMC2335261

[29] Wanhainen A, Van Herzeele I, Bastos Goncalves F et al. Editor's choice -- European Society for Vascular Surgery (ESVS) 2024 clinical practice guidelines on the Management of Abdominal Aorto-Iliac Artery Aneurysms. Eur J Vasc Endovasc Surg 2024; 67(2): 192–331. 38307694 10.1016/j.ejvs.2023.11.002

[30] Griffin S M, Lamb P J, Dresner S M, Richardson D L, Hayes N. Diagnosis and management of a mediastinal leak following radical oesophagectomy. Br J Surg 2001; 88(10): 1346–51. 11578290 10.1046/j.0007-1323.2001.01918.x

[31] Low D E . Diagnosis and management of anastomotic leaks after esophagectomy. J Gastrointest Surg 2011; 15(8): 1319–22. 10.1007/s11605-011-1511-0. 21557015

[32] Martin L W, Hofstetter W, Swisher S G, Roth J A. Management of intrathoracic leaks following esophagectomy. Adv Surg 2006; 40: 173–90. 17163101 10.1016/j.yasu.2006.05.010

[33] Murthy S C, Law S, Whooley B P, Alexandrou A, Chu K M, Wong J. Atrial fibrillation after esophagectomy is a marker for postoperative morbidity and mortality. J Thorac Cardiovasc Surg 2003; 126(4): 1162–7. 14566263 10.1016/s0022-5223(03)00974-7

[34] Tsujimoto H, Ono S, Takahata R et al. Systemic inflammatory response syndrome as a predictor of anastomotic leakage after esophagectomy. Surg Today 2012; 42(2): 141–6. 10.1007/s00595-011-0049-9. 22094435

[35] Aiolfi A, Asti E, Rausa E, Bonavina G, Bonitta G, Bonavina L. Use of C-reactive protein for the early prediction of anastomotic leak after esophagectomy: systematic review and Bayesian meta-analysis. PloS One 2018; 13(12): e0209272. 10.1371/journal.pone.0209272. 30557392 PMC6296520

[36] Andreatta E, Buogo A, Asti E, Boveri S, Bonavina L. Comparison of pleural drain amylase and serum C-reactive protein for early detection of intrathoracic esophago-gastric anastomotic leaks. Langenbecks Arch Surg 2022; 407(7): 2715–24. 35581392 10.1007/s00423-022-02550-4 PMC9640430

[37] Asti E, Bonitta G, Melloni M et al. Utility of C-reactive protein as predictive biomarker of anastomotic leak after minimally invasive esophagectomy. Langenbecks Arch Surg 2018; 403(2): 235–44. 10.1007/s00423-018-1663-4. 29516256

[38] Azer M, Miftode S, Bockhorn M, El-Sourani N. Evaluation of the use of inflammatory biomarkers in the early detection of anastomotic leakage after esophagectomy: a retrospective analysis. Surg Open Sci 2022; 10: 12–8. 35800711 10.1016/j.sopen.2022.05.007 PMC9253454

[39] Giulini L, Dubecz A, Solymosi N et al. Prognostic value of chest-tube amylase versus C-reactive protein as screening tool for detection of early anastomotic leaks after Ivor Lewis esophagectomy. J Laparoendosc Adv Surg Tech A 2019; 29(2): 192–7. 30592690 10.1089/lap.2018.0656

[40] McAnena P, Neary C, Doyle C, Kerin M J, McAnena O J, Collins C. Serial CRP levels following oesophagectomy: a marker for anastomotic dehiscence. Ir J Med Sci 2020; 189(1): 277–82. 10.1007/s11845-019-02072-x. 31372815

[41] Noble F, Curtis N, Harris S et al. Risk assessment using a novel score to predict anastomotic leak and major complications after oesophageal resection. J Gastrointest Surg 2012; 16(6): 1083–95. 10.1007/s11605-012-1867-9. 22419007

[42] Park J K, Kim J J, Moon S W. C-reactive protein for the early prediction of anastomotic leak after esophagectomy in both neoadjuvant and non-neoadjuvant therapy case: a propensity score matching analysis. J Thorac Dis 2017; 9(10): 3693–702. 10.21037/jtd.2017.08.125. 29268376 PMC5723863

[43] Prochazka V, Marek F, Kunovsky L et al. C-reactive protein as predictor of anastomotic complications after minimally invasive oesophagectomy. J Minim Access Surg 2019; 15(1): 46–50. 10.4103/jmas.JMAS_254_17. 29595182 PMC6293671

[44] Rat P, Piessen G, Vanderbeken M et al. C-reactive protein identifies patients at low risk of anastomotic leak after esophagectomy. Langenbecks Arch Surg 2022; 407(8): 3377–86. 10.1007/s00423-022-02703-5. 36207546

[45] Stuart S K, Kuypers T J L, Martijnse I S, Heisterkamp J, Matthijsen R A. C-reactive protein and drain amylase: their utility in ruling out anastomotic leakage after minimally invasive Ivor-Lewis esophagectomy. Scand J Gastroenterol 2023; 58(5): 448–52. 10.1080/00365521.2022.2141076. 36346047

[46] Zhang C, Li X K, Hu L W et al. Predictive value of postoperative C-reactive protein-to-albumin ratio in anastomotic leakage after esophagectomy. J Cardiothorac Surg 2021; 16(1): 133. 10.1186/s13019-021-01515-w. 34001160 PMC8130324

[47] Honing J, Pultrum B B, van der Jagt E J, Groen H, Plukker J T. Routine or on demand radiological contrast examination in the diagnosis of anastomotic leakage after esophagectomy. J Surg Oncol 2009; 100(8): 699–702. 19731246 10.1002/jso.21401

[48] Baker E H, Hill J S, Reames M K, Symanowski J, Hurley S C, Salo J C. Drain amylase aids detection of anastomotic leak after esophagectomy. J Gastrointest Oncol 2016; 7(2): 181–8. 10.3978/j.issn.2078-6891.2015.074. 27034784 PMC4783739

[49] Berkelmans G H, Kouwenhoven E A, Smeets B J et al. Diagnostic value of drain amylase for detecting intrathoracic leakage after esophagectomy. World J Gastroenterol 2015; 21(30): 9118–25. 10.3748/wjg.v21.i30.9118. 26290638 PMC4533043

[50] Gao C, Xu G, Wang C, Wang D. Evaluation of preoperative risk factors and postoperative indicators for anastomotic leak of minimally invasive McKeown esophagectomy: a single-center retrospective analysis. J Cardiothorac Surg 2019; 14(1): 46. 10.1186/s13019-019-0864-4. 30819240 PMC6394086

[51] Matsumoto T, Kikuchi H, Haneda R et al. Early detection of anastomotic leakage after esophagectomy using drain amylase levels. Esophagus 2021; 18(3): 522–8. 10.1007/s10388-021-00827-z. 33641017

[52] Perry Y, Towe C W, Kwong J, Ho V P, Linden P A. Serial drain amylase can accurately detect anastomotic leak after esophagectomy and may facilitate early discharge. Ann Thorac Surg 2015; 100(6) discussion 2046-7: 2041–7. 10.1016/j.athoracsur.2015.05.092. 26319485

[53] Yu W S, Jung J, Shin H et al. Amylase level in cervical drain fluid and anastomotic leakage after cervical oesophagogastrostomy. Eur J Cardiothorac Surg 2019; 56(2): 301–6. 10.1093/ejcts/ezz008 30715298

[54] Van Daele E, Vanommeslaeghe H, Peirsman L, Van Nieuwenhove Y, Ceelen W, Pattyn P. Early postoperative systemic inflammatory response as predictor of anastomotic leakage after esophagectomy: a systematic review and meta-analysis. J Gastrointest Surg 2024; 28(5): 757–65. 38704210 10.1016/j.gassur.2024.02.003

[55] Yonis G, Cabalag C S, Link E, Duong C P. Utility of routine oral contrast study for detecting postesophagectomy anastomotic leak - a systematic review and meta-analysis. Dis Esophagus 2019; 32(7): doz011. 10.1093/dote/doz011. 30855088

[56] Murray T E, Morrin M. Comparative diagnostic test accuracy of post-esophagectomy water-soluble computed tomography and fluoroscopic swallow studies: a meta-analysis. Indian J Radiol Imaging 2018; 28(1): 55–60. 10.4103/ijri.IJRI_262_17. 29692528 PMC5894320

[57] Goense L, Stassen P M C, Wessels F J et al. Diagnostic performance of a CT-based scoring system for diagnosis of anastomotic leakage after esophagectomy: comparison with subjective CT assessment. Eur Radiol 2017; 27(10): 4426–34. 10.1007/s00330-017-4802-3. 28357496 PMC5579173

[58] Plat V D, Bootsma B T, Straatman J et al. The clinical suspicion of a leaking intrathoracic esophagogastric anastomosis: the role of CT imaging. J Thorac Dis 2020; 12(12): 7182–92. 10.21037/jtd-20-954. 33447407 PMC7797855

[59] El-Sourani N, Miftode S, Bockhorn M, Arlt A, Meinhardt C. Endoscopic Management of Anastomotic Leakage after Esophageal surgery: ten year analysis in a tertiary university Center. Clin Endosc 2022; 55(1): 58–66. 10.5946/ce.2021.099. 34645084 PMC8831416

[60] Tavares G, Tustumi F, Tristão L S, Bernardo W M. Endoscopic vacuum therapy for anastomotic leak in esophagectomy and total gastrectomy: a systematic review and meta-analysis. Dis Esophagus 2021; 34(5): doaa132. 10.1093/dote/doaa132. 33479749

[61] Murray W, Davey M G, Robb W, Donlon N E. Management of esophageal anastomotic leaks, a systematic review and network meta-analysis. Dis Esophagus 2024; 37(7): doae019. 10.1093/dote/doae019. 38525940

[62] Liu C Q, Ma Y L, Qin Q et al. Epidemiology of esophageal cancer in 2020 and projections to 2030 and 2040. Thorac Cancer 2023; 14(1): 3–11. 36482832 10.1111/1759-7714.14745 PMC9807450

[63] Low D E, Alderson D, Cecconello I et al. International consensus on standardization of data collection for complications associated with esophagectomy: esophagectomy complications consensus group (ECCG). Ann Surg 2015; 262(2): 286–94. 25607756 10.1097/SLA.0000000000001098

[64] Van Dongen G J, Lemmens J M G. ImpRovEd care after eSophageCtomy using an algorithm for postoperative complications - RESCUE trial (RESCUE). ClinicalTrials.gov identifier: NCT06762652. Updated January 1. 2025; Accessed June 17, 2025. https://clinicaltrials.gov/study/NCT06762652.

