# Supplementary material for: International Society for Diseases of the Esophagus consensus on the diagnosis and treatment of anastomotic leak after esophagectomy
Source: Dis Esophagus. 2026 Feb 5;39(1):doag006. doi: 10.1093/dote/doag006 (PMC13078160; doi:10.1093/dote/doag006)
Supplement: doag006_Supplementary_file_ISDE_Consensus_Statement_Management_of_AL [file doag006_supplementary_file_isde_consensus_statement_management_of_al.docx]

**Supplementary file** for **‘***International Society for Diseases of the Esophagus consensus on the diagnosis and treatment of anastomotic leak after esophagectomy’.*

**Content**

[**Supplementary Fig. S1** PRISMA diagram of included studies into the scoping systematic literature review. 2](#_Toc170074179)

[**Supplementary Table S1** Overview of all included articles from scoping systematic literature review 3](#_Toc170074180)

[**Supplementary Table S2** Respondents characteristics from both Delphi survey rounds 8](#_Toc170074181)

[**Supplementary Table S3** First-round Delphi survey results and corresponding consensus 10](#_Toc170074182)

[**Supplementary Table S4** Second-round Delphi survey results and corresponding consensus 18](#_Toc170074183)

[**Supplementary Table S5** First-round Delphi survey consensus stratified by hospital volume and continent 28](#_Toc170074184)

[**Supplementary Table S6** Second-round Delphi survey consensus stratified by hospital volume and continent 36](#_Toc170074185)

[**Stage 3:** Guideline development meetings – Overview of the recommendations regarding the diagnosis and treatment of anastomotic leak after esophagectomy, including the level of evidence, relevant articles, the strength of recommendation, corresponding results from the two-round Delphi survey and additional remarks and considerations of the expert panel. 45](#_Toc170074186)

# **Supplementary Fig. S1** PRISMA diagram of included studies into the scoping systematic literature review.

Identification

Screening

Eligibility

Included

Records identified through database searching.

Pubmed (n=2.163)

Embase (n=3.524)

Cochrane (n=154)

Additional records identified through other sources

(n=2)

Records identified

(n=5.843)

Duplicates removed

(n=2.079]

)

Records screened

(n=3.764)

Records excluded

(n=3.481)

Full-text articles assessed for eligibility

(n= 283)

Full-text articles excluded, with reasons

(n=165)

- Mixed surgical procedures (n=45)

- Mixed surgical indications (n=34)

- Full (English) text unavailable (n=17)

- Out of scope (n=69)

Studies included in qualitative synthesis

(n= 118)

| **Supplementary Table S1** Overview of all included articles from scoping systematic literature review | | | | | | | |
| --- | --- | --- | --- | --- | --- | --- | --- |
| **First author** | **Year** | **Study design** | **Study domain** | **Modality studied** | **Patients, n** | **AL, n (%)** | **NOS-score** |
| *Review studies* | | | | | | | |
| Aiolfi | 2018 | Systematic review and meta-analysis | Diagnosis | CRP | 850 | 98 (11.5) | - |
| Barbaro | 2021 | Systematic review | Diagnosis | Diagnosis in general | - | - | - |
| De Mooij | 2019 | Systematic review | Diagnosis | Biomarkers | - | - | - |
| Jones | 2015 | Review | Diagnosis | Routine oral contrast study (cAL) | - | - | - |
| Jones | 2015 | Review | Diagnosis | Routine oral contrast study (iAL) | - | - | - |
| sMoon | 2019 | Review | Diagnosis | Early detection of complications | - | - | - |
| Murray | 2018 | Systematic review and meta-analysis | Diagnosis | CT, oral contrast study | 187 | 25 (13.5) | - |
| Veziant | 2022 | Review | Diagnosis | Imaging of complications | - | - | - |
| Yonis | 2019 | Systematic review and meta-analysis | Diagnosis | Routine oral contrast study | 3583 | 315 (8.8) | - |
| Chevallay | 2020 | Review | Treatment | Management in general | - | - | - |
| Fabian | 2021 | Review | Treatment | Management in general | - | - | - |
| Famiglietti | 2020 | Review | Treatment | Management in general | - | - | - |
| Hua | 2022 | Systematic review | Treatment | Treatment in general | - | - | - |
| Hummel | 2017 | Review | Treatment | Surgical treatment | - | - | - |
| Lee | 2020 | Review | Treatment | Endoscopic vacuum therapy | - | - | - |
| Martin | 2006 | Review | Treatment | Management in general (iAL) | - | - | - |
| Plat | 2017 | Systematic review | Treatment | Tissue adhesives | - | - | - |
| Schaheen | 2014 | Systematic review | Treatment | Management in general (iAL) | - | - | - |
| Tavares | 2021 | Systematic review and meta-analysis | Treatment | Endoscopic vacuum therapy | - | 559 | - |
| Urschel | 1995 | Review | Treatment | Treatment in general | - | - | - |
| Verstegen | 2019 | Systematic review | Treatment | Management in general | - | - | - |
| Wichmann | 2022 | Review | Treatment | Endoscopic management | - | - | - |
| Yeung | 2020 | Review | Treatment | Management in general | - | - | - |
| Birla | 2019 | Systematic review | Both | General overview | - | - | - |
| Cassivi | 2004 | Review | Both | General overview | - | - | - |
| Fabbi | 2021 | Review | Both | General overview | - | - | - |
| Grimminger | 2018 | Review | Both | General overview | - | - | - |
| Jones | 2015 | Review | Both | General overview | - | - | - |
| Lebedis | 2013 | Review | Both | General overview | - | - | - |
| Low | 2011 | Review | Both | General overview | - | - | - |
| Mardin | 2012 | Review | Both | General overview | - | - | - |
| Mitchell | 2006 | Review | Both | General overview | - | - | - |
| Nguyen | 2007 | Review | Both | General overview (iAL) | - | - | - |
| Paul | 2003 | Review | Both | General overview | - | - | - |
| Raju | 2006 | Review | Both | Endoscopic management | - | - | - |
| Schuchert | 2004 | Review | Both | General overview | - | - | - |
| *Prospective cohort studies* | | | | | | | |
| Ip | 2017 | Prospective cohort | Diagnosis | Serum lactate | 136 | 18 (13.2) | 7 |
| Lemmens | 2023 | Prospective cohort | Diagnosis | Performance of diagnostic algorithm | 245 | 57 (23.3) | 7 |
| Plat | 2019 | Prospective cohort | Diagnosis | Urinary volatile organic compounds | 31 | 9 (29.0) | 6 |
| Song | 2020 | Prospective cohort | Diagnosis | Ultrathin endoscopy, CT | 71 | 14 (19.7) | 7 |
| Strauss | 2010 | Prospective cohort | Diagnosis | CT, oral contrast study | 97 | 13 (13.4) | 7 |
| Upponi | 2008 | Prospective cohort | Diagnosis | CT, oral contrast study | 52 | 15 (28.8) | 5 |
| Griffiths | 2022 | Prospective cohort | Treatment | Management in general | 2247 | 319 (14.2) | 7 |
| *Retrospective cohort studies* | | | | | | | |
| Andreatta | 2022 | Retrospective cohort | Diagnosis | Drain amylase, CRP | 121 | 12 (9.9) | 7 |
| Asti | 2018 | Retrospective cohort | Diagnosis | CRP | 243 | 29 (11.9) | 7 |
| Azer | 2022 | Retrospective cohort | Diagnosis | CRP, WBC | 147 | 28 (19.0) | 7 |
| Baker | 2016 | Retrospective cohort | Diagnosis | Drain amylase | 100 | 13 (13.0) | 7 |
| Berkelmans | 2015 | Retrospective cohort | Diagnosis | Drain amylase | 89 | 15 (16.9) | 7 |
| Boone | 2008 | Retrospective cohort | Diagnosis | Oral contrast study | 252 | 36 (14.3) | 7 |
| Cools-Lartique | 2014 | Retrospective cohort | Diagnosis | Oral contrast study | 221 | 30 (13.6) | 7 |
| El-Sourani | 2022 | Retrospective cohort | Diagnosis | CT, endoscopy | 185 | 33 (17.8) | 7 |
| Gao | 2019 | Retrospective cohort | Diagnosis | Serum prealbumin, drain amylase | 96 | 12 (12.5) | 7 |
| Giulini | 2019 | Retrospective cohort | Diagnosis | CRP, drain amylase | 80 | 6 (7.5) | 7 |
| Goense | 2017 | Retrospective cohort | Diagnosis | CT-scoring system | 405 | 54 (13.3) | 7 |
| Honing | 2009 | Retrospective cohort | Diagnosis | Fever, WBC, sepsis | 211 | 35 (16.6) | 7 |
| Huang | 2022 | Retrospective cohort | Diagnosis | Serum prealbumin | 255 | 18 (7.1) | 7 |
| Kang | 2022 | Retrospective cohort | Diagnosis | CT, oral contrast study | 76 | 19 (25.0) | 7 |
| Lantos | 2013 | Retrospective cohort | Diagnosis | CT, oral contrast study | 29 | 14 (48.3) | 7 |
| Li | 2019 | Retrospective cohort | Diagnosis | Coeffiecient of procalciton and blood G | 71 | 47 (66.2) | 7 |
| Lin | 2017 | Retrospective cohort | Diagnosis | Endoscopy | 65 | 15 (23.1) | 7 |
| Matsumoto | 2021 | Retrospective cohort | Diagnosis | Drain amylase | 134 | 16 (11.9) | 7 |
| Murthy | 2003 | Retrospective cohort | Diagnosis | Atrial fibrillation | 921 | 198 (21.5) | 9 |
| McAnena | 2020 | Retrospective cohort | Diagnosis | CRP | 102 | 5 (4.9) | 7 |
| Nederlof | 2017 | Retrospective cohort | Diagnosis | Oral contrast study, endoscopy | 308 | 70 (22.7) | 7 |
| Noble | 2012 | Retrospective cohort | Diagnosis | Albumin, CRP, WBC | 258 | 26 (10.1) | 7 |
| Page | 2013 | Retrospective cohort | Diagnosis | Routine endoscopy | 100 | 6 (6.0) | 5 |
| Park | 2017 | Retrospective cohort | Diagnosis | CRP | 201 | 23 (11.4) | 7 |
| Perry | 2015 | Retrospective cohort | Diagnosis | Drain amylase | 146 | 35 (24.0) | 7 |
| Plat | 2020 | Retrospective cohort | Diagnosis | CT | 167 | 25 (15.0) | 7 |
| Prochazka | 2018 | Retrospective cohort | Diagnosis | CRP | 40 | 11 (27.5) | 5 |
| Rat | 2022 | Retrospective cohort | Diagnosis | CRP | 585 | 69 (11.8) | 7 |
| Scheufele | 2022 | Retrospective cohort | Diagnosis | aPTT, serum lactate | 138 | 33 (23.9) | 7 |
| Shao | 2019 | Retrospective cohort | Diagnosis | CRP-to-albumin ratio | 450 | 55 (12.2) | 7 |
| Shoji | 2018 | Retrospective cohort | Diagnosis | Air bubble sign, CT, oral contrast study | 142 | 22 (15.5) | 7 |
| Sohda | 2020 | Retrospective cohort | Diagnosis | Monitoring hemodynamics | 39 | 8 (20.5) | 7 |
| Song | 2017 | Retrospective cohort | Diagnosis | Plasma cytokines | 183 | 16 (8.7) | 7 |
| Stuart | 2022 | Retrospective cohort | Diagnosis | CRP, drain amylase | 193 | 30 (15.5) | 7 |
| Takesue | 2015 | Retrospective cohort | Diagnosis | Hyperbilirubinemia | 200 | 35 (17.5) | 7 |
| Tsujimoto | 2012 | Retrospective cohort | Diagnosis | Systemic Inflammatory response syndrome | 61 | 9 (14.8) | 7 |
| Wu | 2021 | Retrospective cohort | Diagnosis | Neutrophil count, neutrophil-to-lymphocyte ratio, platelet-to-lymphocyte ratio | 198 | 25 (12.6) | 7 |
| Yu | 2019 | Retrospective cohort | Diagnosis | Drain amylase | 99 | 10 (10.1) | 7 |
| Zhang | 2021 | Retrospective cohort | Diagnosis | Albumin, CRP, CRP-to-albumin ratio | 273 | 34 (12.5) | 7 |
| Al-issa | 2014 | Retrospective cohort | Treatment | Stent | 209 | 20 (9.6) | 7 |
| Bhasin | 2000 | Retrospective cohort | Treatment | Endoscopic dilation | 46 | 8 (17.4) | 5 |
| Chen | 2020 | Retrospective cohort | Treatment | Fibrin sealant | 179 | 7 (3.9) | 7 |
| Chon | 2022 | Retrospective cohort | Treatment | Endoscopic vacuum therapy | 157 | 21 (13.4) | 7 |
| El-sourani | 2022 | Retrospective cohort | Treatment | Endoscopic management | 141 | 28 (19.9) | 7 |
| El-sourani | 2023 | Retrospective cohort | Treatment | Endoscopic vacuum therapy | - | 17 | 7 |
| Guo | 2019 | Retrospective cohort | Treatment | Naso-mediastinal drainage | 971 | 51 (5.3) | 5 |
| Hünerbein | 2004 | Retrospective cohort | Treatment | Stent | 204 | 19 (9.3) | 7 |
| Jiang | 2011 | Retrospective cohort | Treatment | Naso-mediastinal drainage | 2954 | 38 (1.3) | 5 |
| Kauer | 2008 | Retrospective cohort | Treatment | Stent | 269 | 12 (4.5) | 5 |
| Ma | 2018 | Retrospective cohort | Treatment | Endoscopic interventions | 2596 | 263 (10.1) | 7 |
| Min | 2019 | Retrospective cohort | Treatment | Endoscopic vacuum therapy | - | 20 | 6 |
| Page | 2005 | Retrospective cohort | Treatment | Surgical treatment | 389 | 23 (5.9) | 7 |
| Plum | 2019 | Retrospective cohort | Treatment | Stent | - | 70 | 7 |
| Ruiz de Angelo | 2018 | Retrospective cohort | Treatment | Stent | 85 | 10 (11.8) | 4 |
| Schweigert | 2014 | Retrospective cohort | Treatment | Stent | 356 | 49 (13.8) | 7 |
| Shuto | 2017 | Retrospective cohort | Treatment | Naso-mediastinal drainage | 365 | 50 (13.7) | 7 |
| Smith | 2020 | Retrospective cohort | Treatment | Stent | 111 | 11 (9.9) | 7 |
| Ubels | 2023 | Retrospective cohort | Treatment | Treatment principles | - | 1508 | 9 |
| Xu | 2016 | Retrospective cohort | Treatment | Conventional versus complex treatment | 1952 | 67 (3.4) | 7 |
| Yin | 2012 | Retrospective cohort | Treatment | Naso-mediastinal drainage | - | 28 | 5 |
| Yin | 2021 | Retrospective cohort | Treatment | Three-tube insertion | 875 | 43 (4.9) | 7 |
| Zhang | 2017 | Retrospective cohort | Treatment | Chest drainage, naso-mediastinal drainage | 1527 | 67 (4.4) | 5 |
| Zhang | 2021 | Retrospective cohort | Treatment | Endoscopic vacuum therapy | 259 | 72 (27.8) | 7 |
| Zhong | 2021 | Retrospective cohort | Treatment | Endoscopic clip and tissue sealant | 3919 | 203 (5.2) | 7 |
| Griffin | 2001 | Retrospective cohort | Both | Oral contrast study, endoscopy and conservative versus surgical management | 291 | 19 (6.5) | 7 |
| Turkyilmaz | 2009 | Retrospective cohort | Both | Developing an algorithm | 354 | 25 (7.1) | 7 |
| Ubels | 2022 | Retrospective cohort | Both | Grading leak severity | - | 1509 | 7 |
| *Case series* | | | | | | | |
| Bi | 2019 | Case series | Treatment | Three-tube method | - | 23 | - |
| Hua | 2019 | Case series | Treatment | Bovine pericardium patch repair | - | 7 | - |
| Nakajima | 2014 | Case series | Treatment | Sternocleidomastoid flap repair | - | 8 | - |
| Qin | 2010 | Case series | Treatment | Drain through fistula | - | 5 | - |
| Tashiro | 2021 | Case series | Treatment | Polyglycolic acid and fibrin glue | - | 10 | - |
| Wu | 2017 | Case series | Treatment | Novel stent | - | 27 | - |
| *Other* | | | | | | | |
| Ubels | 2022 | Mixed-methods | Treatment | Various treatment strategies | - | - | - |
| Hagens | 2018 | Questionnaire study | Both | Various opinions on AL detection and management | - | - | - |
| *NOS; Newcastle-Ottawa scale. AL; anastomotic leak. cAL; cervical AL. iAL; intrathoracic AL. CT; computed tomography. WBC; white blood count. CRP; C-reactive protein. aPTT; activated partial thromboplastin time.* | | | | | | | |

| **Supplementary Table S2** Respondents characteristics from both Delphi survey rounds | | |
| --- | --- | --- |
|  | **Round 1** | **Round 2** |
| Respondents (n) | 106 | 136 |
| Number of countries (n) | 35 | 39 |
| Continental distribution (n (%)) |  |  |
| Asia | 17 (16.0) | 14 (10.3) |
| Africa | 4 (3.8) | 5 (3.7) |
| North America | 7 (6.6) | 10 (7.4) |
| South America | 6 (5.7) | 8 (5.9) |
| Europe | 67 (63.2) | 84 (61.8) |
| Oceania | 5 (4.7) | 14 (10.3) |
| Medical specialty (n (%)) |  |  |
| Surgeon | 99 (93.4) | 129 (94.9) |
| Surgical fellow | 5 (4.7) | 7 (5.1) |
| Gastro-enterologist | 2 (1.9) | 0 |
| Years of experience with diagnosing and/or treating AL (median [IQR]) | 15 [10 - 22] | 15 [7 - 22] |
| Annual hospital volume (n (%)) |  |  |
| Low (< 20) | 7 (6.6) | 22 (16.2) |
| Middle (20 – 59) | 52 (49.1) | 64 (47.1) |
| High (≥ 60) | 47 (44.3) | 50 (36.8) |
| Frequency of surgical approach (median [IQR]) |  |  |
| Transhiatal | 5 [0 - 10] | 5 [0 - 10] |
| Transthoracic | 95 [85 - 100] | 87.5 [70 - 95] |
| Transcervical | *-* | 0 [0 - 10] |
| Frequency of surgical technique (median [IQR]) |  |  |
| MIE | 27.5 [5 - 74] | 35 [0 - 70] |
| Hybrid | 5 [0 - 24] | 10 [0 - 30] |
| Robotic | 0 [0 - 28] | 0 [0 - 20] |
| Open | 7.5 [5 - 50] | 10 [5 - 44] |
| Frequency of anastomosis location (median [IQR]) |  |  |
| Cervical | 25 [10 - 50] | 20 [10 - 50] |
| Intrathoracic | 75 [50 - 90] | 80 [50 - 90] |
| Preferred postoperative feeding method (n (%)) |  |  |
| Direct (oral) feeding | 24 (23.1) | 30 (22.1) |
| Enteral feeding via jejunostomy | 48 (46.2) | 57 (41.9) |
| Enteral feeding via nasojejunal tube | 11 (10.6) | 17 (12.5) |
| Total parenteral nutrition | 15 (14.4) | 25 (18.4) |
| Other | 6 (5.8) | 7 (5.1) |
| Presence of standardized and written protocol for diagnosing AL (yes, n (%)) | 46 (43.4) | 55 (40.4) |
| Presence of standardized and written protocol for treating AL (yes, n (%)) | 28 (26.4) | 31 (22.8) |
| Frequency of availability of therapeutic modalities (yes, n (%)) |  |  |
| Radiological drainage tube (ultrasound or CT-scan guided) | 101 (95.3) | 129 (94.9) |
| Radiological stent placement | 49 (46.2) | 66 (48.5) |
| Endoscopic drainage | 90 (84.9) | 120 (88.2) |
| Endoscopic clipping or suturing | 90 (84.9) | 119 (87.5) |
| Endoscopic tissue adhesives | 51 (48.1) | 78 (57.4) |
| Endoscopic stent placement | 98 (92.5) | 129 (94.9) |
| Endoscopic vacuum therapy | 92 (86.8) | 108 (79.4) |
| Endoscopic vacuum therapy combined with stent | 39 (36.8) | 69 (50.7) |
| *AL; anastomotic leak. IQR; interquartile range. MIE; minimally invasive esophagectomy. CT; computed tomography.* | | |

| **Supplementary Table S3** First-round Delphi survey results and corresponding consensus | | | | | | | | |  |  |  |  |  |  |  |  |
| --- | --- | --- | --- | --- | --- | --- | --- | --- | --- | --- | --- | --- | --- | --- | --- | --- |
| **Statement** | **Strongly disagree (n)** | **Disagree (n)** | **Somewhat disagree (n)** | **Somewhat agree**  **(n)** | **Agree**  **(n)** | **Strongly agree**  **(n)** | **Consensus^†^ (%)** | **No comment (n)** |  |  |  |  |  |  |  |  |
| *Diagnosis* | | | | | | | | |  |  |  |  |  |  |  |  |
| Indicate for each clinical sign if you agree that that clinical sign is useful in the diagnostic process of anastomotic leak after esophagectomy. | | | | | | | | |  |  |  |  |  |  |  |  |
| Tachycardia | 1 | 2 | 0 | 20 | 46 | 37 | 78 | 0 |  |  |  |  |  |  |  |  |
| Cardiac arrhythmia (e.g. atrial fibrillation) | 1 | 4 | 2 | 33 | 30 | 35 | 62 | 1 |  |  |  |  |  |  |  |  |
| Fever (>38.5◦C) | 1 | 1 | 1 | 13 | 40 | 50 | 85 | 0 |  |  |  |  |  |  |  |  |
| Respiratory distress (e.g. tachypnea, oxygen delivery increase) | 0 | 2 | 2 | 16 | 40 | 45 | 81 | 1 |  |  |  |  |  |  |  |  |
| Subcutaneous emphysema | 2 | 10 | 9 | 33 | 20 | 30 | 48 | 2 |  |  |  |  |  |  |  |  |
| Chest pain | 2 | 11 | 10 | 36 | 33 | 12 | 43 | 2 |  |  |  |  |  |  |  |  |
| Redness and swelling in the associated neck and chest incisions | 1 | 2 | 4 | 16 | 32 | 46 | 77 | 5 |  |  |  |  |  |  |  |  |
| Changed aspect of postoperative drain fluids | 0 | 1 | 1 | 6 | 24 | 70 | 92 | 4 |  |  |  |  |  |  |  |  |
| Increased output of postoperative drain | 1 | 5 | 13 | 34 | 33 | 19 | 50 | 1 |  |  |  |  |  |  |  |  |
| Indicate for each biochemical test if you agree that that biochemical test is useful in the diagnostic process of anastomotic leak after esophagectomy. | | | | | | | | |  |  |  |  |  |  |  |  |
| Serum C-reactive protein (CRP) | 0 | 1 | 1 | 9 | 28 | 66 | 90 | 1 |  |  |  |  |  |  |  |  |
| Drain amylase | 0 | 7 | 1 | 17 | 31 | 39 | 74 | 11 |  |  |  |  |  |  |  |  |
| Serum white blood count (WBC) | 0 | 2 | 4 | 21 | 46 | 33 | 75 | 0 |  |  |  |  |  |  |  |  |
| Serum CRP-to-albumin ratio | 0 | 10 | 9 | 26 | 17 | 6 | 34 | 38 |  |  |  |  |  |  |  |  |
| Serum procalcitonin | 1 | 8 | 11 | 19 | 28 | 14 | 52 | 25 |  |  |  |  |  |  |  |  |
| Serum prealbumin | 5 | 15 | 13 | 20 | 8 | 1 | 15 | 44 |  |  |  |  |  |  |  |  |
| Plasma cytokines | 2 | 11 | 11 | 15 | 6 | 0 | 13 | 61 |  |  |  |  |  |  |  |  |
| Serum neutrophil count | 0 | 6 | 14 | 23 | 30 | 12 | 49 | 21 |  |  |  |  |  |  |  |  |
| Serum platelet-to-lymphocyte ratio | 2 | 9 | 17 | 13 | 12 | 1 | 24 | 52 |  |  |  |  |  |  |  |  |
| Neutrophil-to-lymphocyte ratio | 2 | 12 | 16 | 13 | 14 | 2 | 27 | 47 |  |  |  |  |  |  |  |  |
| Serum bilirubin | 9 | 31 | 25 | 15 | 2 | 0 | 2 | 24 |  |  |  |  |  |  |  |  |
| **Statement** | **Strongly disagree (n)** | **Disagree (n)** | **Somewhat disagree (n)** | **Somewhat agree**  **(n)** | **Agree**  **(n)** | **Strongly agree**  **(n)** | **Consensus (%)** | **No comment (n)** |  |  |  |  |  |  |  |  |
| The coefficient of serum procalcitonin and blood G | 4 | 12 | 16 | 14 | 2 | 1 | 6 | 57 |  |  |  |  |  |  |  |  |
| Serum lactate | 3 | 15 | 13 | 29 | 21 | 12 | 36 | 13 |  |  |  |  |  |  |  |  |
| Indicate for each postoperative day (POD) if you agree that monitoring serum C-reactive protein (CRP) on that day is useful in the diagnostic process of anastomotic leak. | | | | | | | | |  |  |  |  |  |  |  |  |
| POD 1 | 12 | 27 | 15 | 10 | 20 | 10 | 32 | 12 |  |  |  |  |  |  |  |  |
| POD 2 | 12 | 23 | 9 | 16 | 16 | 13 | 33 | 17 |  |  |  |  |  |  |  |  |
| POD 3 | 4 | 8 | 7 | 16 | 27 | 35 | 64 | 9 |  |  |  |  |  |  |  |  |
| POD 4 | 3 | 6 | 2 | 18 | 28 | 34 | 68 | 15 |  |  |  |  |  |  |  |  |
| POD 5 | 0 | 2 | 2 | 10 | 32 | 53 | 86 | 7 |  |  |  |  |  |  |  |  |
| POD 6 | 1 | 4 | 2 | 14 | 35 | 33 | 76 | 17 |  |  |  |  |  |  |  |  |
| POD 7 | 2 | 3 | 4 | 17 | 33 | 35 | 72 | 12 |  |  |  |  |  |  |  |  |
| POD 8 | 2 | 8 | 8 | 20 | 27 | 20 | 55 | 21 |  |  |  |  |  |  |  |  |
| POD 9 or longer | 3 | 12 | 10 | 22 | 22 | 15 | 44 | 22 |  |  |  |  |  |  |  |  |
| The trend in CRP values is useful in the decision for additional diagnostics regarding anastomotic leak. | 2 | 2 | 2 | 5 | 29 | 57 | 89 | 9 |  |  |  |  |  |  |  |  |
| Indicate for each postoperative day (POD) if you agree that monitoring drain amylase on that day is useful in the diagnostic process of anastomotic leak. | | | | | | | | |  |  |  |  |  |  |  |  |
| POD 1 | 7 | 30 | 10 | 10 | 7 | 7 | 20 | 35 |  |  |  |  |  |  |  |  |
| POD 2 | 5 | 25 | 8 | 14 | 13 | 8 | 29 | 33 |  |  |  |  |  |  |  |  |
| POD 3 | 3 | 15 | 4 | 16 | 23 | 13 | 49 | 32 |  |  |  |  |  |  |  |  |
| POD 4 | 2 | 13 | 3 | 12 | 24 | 20 | 60 | 32 |  |  |  |  |  |  |  |  |
| POD 5 | 2 | 13 | 2 | 9 | 21 | 27 | 65 | 32 |  |  |  |  |  |  |  |  |
| POD 6 | 2 | 15 | 3 | 9 | 19 | 21 | 58 | 37 |  |  |  |  |  |  |  |  |
| POD 7 | 2 | 16 | 5 | 8 | 20 | 20 | 56 | 35 |  |  |  |  |  |  |  |  |
| POD 8 | 2 | 18 | 5 | 8 | 17 | 15 | 49 | 41 |  |  |  |  |  |  |  |  |
| POD 9 or longer | 2 | 19 | 4 | 10 | 15 | 14 | 45 | 42 |  |  |  |  |  |  |  |  |
| **Statement** | **Strongly disagree (n)** | **Disagree (n)** | **Somewhat disagree (n)** | **Somewhat agree**  **(n)** | **Agree**  **(n)** | **Strongly agree**  **(n)** | **Consensus (%)** | **No comment (n)** |  |  |  |  |  |  |  |  |
| The trend in drain amylase values is useful in the decision for additional diagnostics regarding anastomotic leak. | 7 | 13 | 7 | 13 | 21 | 15 | 47 | 30 |  |  |  |  |  |  |  |  |
| Screening for anastomotic leak in asymptomatic patients should be performed by …. | | | | | | | | |  |  |  |  |  |  |  |  |
| Routine contrast radiography | 16 | 36 | 6 | 7 | 27 | 9 | 36 | 5 |  |  |  |  |  |  |  |  |
| Routine computed tomography (CT) scan | 20 | 37 | 11 | 11 | 9 | 12 | 21 | 6 |  |  |  |  |  |  |  |  |
| Routine endoscopy | 24 | 41 | 13 | 13 | 4 | 5 | 9 | 6 |  |  |  |  |  |  |  |  |
| Screening in asymptomatic patients should not be performed | 7 | 15 | 15 | 14 | 24 | 27 | 50 | 4 |  |  |  |  |  |  |  |  |
| If you suspect cervical anastomotic leak based on clinical sign(s) and/or biochemical test(s), indicate for the following diagnostic assessments if you agree that it should be the first choice. | | | | | | | | |  |  |  |  |  |  |  |  |
| Bedside opening of the cervical incision | 3 | 12 | 9 | 16 | 30 | 33 | 61 | 3 |  |  |  |  |  |  |  |  |
| Contrast radiography | 6 | 19 | 9 | 29 | 21 | 15 | 36 | 7 |  |  |  |  |  |  |  |  |
| Computed tomography (CT) scan | 2 | 4 | 2 | 16 | 33 | 43 | 76 | 6 |  |  |  |  |  |  |  |  |
| Endoscopy | 5 | 13 | 13 | 25 | 32 | 10 | 43 | 8 |  |  |  |  |  |  |  |  |
| CT scan and endoscopy | 5 | 13 | 11 | 22 | 24 | 25 | 49 | 6 |  |  |  |  |  |  |  |  |
| If you suspect intrathoracic anastomotic leak based on clinical sign(s) and/or biochemical test(s), indicate for the following diagnostic assessments if you agree that it should be the first choice. | | | | | | | | |  |  |  |  |  |  |  |  |
| Contrast radiography | 10 | 19 | 12 | 23 | 20 | 19 | 38 | 3 |  |  |  |  |  |  |  |  |
| Computed tomography (CT) scan | 0 | 4 | 1 | 11 | 21 | 64 | 84 | 5 |  |  |  |  |  |  |  |  |
| Endoscopy | 1 | 9 | 5 | 24 | 41 | 20 | 61 | 6 |  |  |  |  |  |  |  |  |
| CT scan and endoscopy | 1 | 6 | 5 | 18 | 32 | 42 | 71 | 2 |  |  |  |  |  |  |  |  |
| When an anastomotic leak is diagnosed on CT scan, it is useful to perform additional endoscopy. | 0 | 5 | 5 | 14 | 27 | 54 | 77 | 1 |  |  |  |  |  |  |  |  |
| When an anastomotic leak is diagnosed on endoscopy, it is useful to perform additional CT scan. | 0 | 4 | 0 | 7 | 27 | 67 | 90 | 1 |  |  |  |  |  |  |  |  |
| **Statement** | **Strongly disagree (n)** | **Disagree (n)** | **Somewhat disagree (n)** | **Somewhat agree**  **(n)** | **Agree**  **(n)** | **Strongly agree**  **(n)** | **Consensus (%)** | **No comment (n)** |  |  |  |  |  |  |  |  |
|  |  |  |  |  |  |  |  |  |  |  |  |  |  |  |  |  |
| The size of the defect should be measured during endoscopy in order to guide anastomotic leak treatment. | 0 | 3 | 6 | 14 | 35 | 47 | 78 | 1 |  |  |  |  |  |  |  |  |
| A ‘leak severity score’ predicting the risk of 90-day mortality is useful to guide anastomotic leak treatment. | 0 | 10 | 12 | 24 | 28 | 18 | 50 | 14 |  |  |  |  |  |  |  |  |
| *Treatment* | | | | | | | | |  |  |  |  |  |  |  |  |
| Indicate for each of the following treatment options if you agree that it should be part of non-invasive management/supportive care of all anastomotic leak patients. | | | | | | | | |  |  |  |  |  |  |  |  |
| Broad spectrum antibiotics (according to local protocol) | 0 | 1 | 0 | 0 | 25 | 80 | 99 | 0 |  |  |  |  |  |  |  |  |
| Antifungal therapy (according to local protocol) | 0 | 3 | 9 | 21 | 34 | 38 | 69 | 1 |  |  |  |  |  |  |  |  |
| Nil-by-mouth | 0 | 3 | 10 | 9 | 30 | 54 | 79 | 0 |  |  |  |  |  |  |  |  |
| Clear water diet | 10 | 25 | 16 | 28 | 19 | 6 | 24 | 2 |  |  |  |  |  |  |  |  |
| Feeding support (e.g. enteral feeding using jejunal tube placement, jejunostomy or parenteral feeding) | 0 | 0 | 0 | 2 | 19 | 84 | 98 | 1 |  |  |  |  |  |  |  |  |
| Antacids | 1 | 7 | 8 | 19 | 34 | 32 | 65 | 5 |  |  |  |  |  |  |  |  |
| The next statements are formulated regarding patients with cervical leak with only local manifestations and no fluid collections. Indicate for each of the following statements if you agree with that statement. | | | | | | | | |  |  |  |  |  |  |  |  |
| Only non-invasive management/supportive care should be performed | 1 | 10 | 20 | 20 | 28 | 25 | 51 | 2 |  |  |  |  |  |  |  |  |
| Additional drainage interventions should be performed (e.g. opening cervical incision, EVT, drainage tube) | 4 | 8 | 8 | 19 | 34 | 31 | 63 | 2 |  |  |  |  |  |  |  |  |
| Additional leak closure interventions should be performed (e.g. stent, endoscopic tissue adhesives, surgical closure) | 15 | 35 | 23 | 17 | 12 | 1 | 13 | 3 |  |  |  |  |  |  |  |  |
| **Statement** | **Strongly disagree (n)** | **Disagree (n)** | **Somewhat disagree (n)** | **Somewhat agree**  **(n)** | **Agree**  **(n)** | **Strongly agree**  **(n)** | **Consensus (%)** | **No comment (n)** |  |  |  |  |  |  |  |  |
| The next statements are formulated regarding patients with cervical leak with mediastinal fluid collections. Indicate for each of the following statements if you agree with that statement. | | | | | | | | |  |  |  |  |  |  |  |  |
| Only non-invasive management/supportive care should be performed | 18 | 41 | 19 | 15 | 5 | 2 | 7 | 6 |  |  |  |  |  |  |  |  |
| Additional drainage interventions should be performed (e.g. opening cervical incision, EVT, drainage tube) | 0 | 0 | 1 | 8 | 31 | 62 | 91 | 4 |  |  |  |  |  |  |  |  |
| Additional leak closure interventions should be performed (e.g. stent, endoscopic tissue adhesives, surgical closure) | 4 | 12 | 21 | 18 | 27 | 18 | 45 | 6 |  |  |  |  |  |  |  |  |
| The next statements are formulated regarding patients with cervical leak with pleural fluid collections. Indicate for each of the following statements if you agree with that statement. | | | | | | | | |  |  |  |  |  |  |  |  |
| Only non-invasive management/supportive care should be performed | 31 | 33 | 19 | 11 | 4 | 4 | 8 | 4 |  |  |  |  |  |  |  |  |
| Additional drainage interventions should be performed (e.g. opening cervical incision, EVT, drainage tube) | 1 | 0 | 0 | 7 | 28 | 67 | 92 | 3 |  |  |  |  |  |  |  |  |
| Additional leak closure interventions should be performed (e.g. stent, endoscopic tissue adhesives, surgical closure) | 4 | 11 | 18 | 8 | 33 | 26 | 59 | 6 |  |  |  |  |  |  |  |  |
| The next statements are formulated regarding patients with intrathoracic leak with only local manifestations and no fluid collections. Indicate for each of the following statements if you agree with that statement. | | | | | | | | |  |  |  |  |  |  |  |  |
| Only non-invasive management/supportive care should be performed | 5 | 19 | 7 | 18 | 34 | 21 | 53 | 2 |  |  |  |  |  |  |  |  |
| Additional drainage interventions should be performed (e.g. EVT, drainage tube) | 6 | 15 | 16 | 31 | 19 | 16 | 34 | 3 |  |  |  |  |  |  |  |  |
| **Statement** | **Strongly disagree (n)** | **Disagree (n)** | **Somewhat disagree (n)** | **Somewhat agree**  **(n)** | **Agree**  **(n)** | **Strongly agree**  **(n)** | **Consensus (%)** | **No comment (n)** |  |  |  |  |  |  |  |  |
| Additional leak closure interventions should be performed (e.g. stent, endoscopic tissue adhesives, surgical closure) | 9 | 22 | 17 | 19 | 16 | 22 | 36 | 1 |  |  |  |  |  |  |  |  |
| The next statements are formulated regarding patients with intrathoracic leak with mediastinal fluid collections. Indicate for each of the following statements if you agree with that statement. | | | | | | | | |  |  |  |  |  |  |  |  |
| Only non-invasive management/supportive care should be performed | 32 | 33 | 19 | 13 | 4 | 3 | 7 | 2 |  |  |  |  |  |  |  |  |
| Additional drainage interventions should be performed (e.g. EVT, drainage tube) | 0 | 0 | 3 | 5 | 39 | 58 | 92 | 1 |  |  |  |  |  |  |  |  |
| Additional leak closure interventions should be performed (e.g. stent, endoscopic tissue adhesives, surgical closure) | 3 | 8 | 7 | 17 | 28 | 42 | 67 | 1 |  |  |  |  |  |  |  |  |
| The next statements are formulated regarding patients with intrathoracic leak with pleural fluid collections. Indicate for each of the following statements if you agree with that statement. | | | | | | | | |  |  |  |  |  |  |  |  |
| Only non-invasive management/supportive care should be performed | 41 | 29 | 16 | 9 | 5 | 4 | 9 | 2 |  |  |  |  |  |  |  |  |
| Additional drainage interventions should be performed (e.g. EVT, drainage tube) | 0 | 0 | 0 | 4 | 29 | 71 | 96 | 2 |  |  |  |  |  |  |  |  |
| Additional leak closure interventions should be performed (e.g. stent, endoscopic tissue adhesives, surgical closure) | 2 | 7 | 6 | 12 | 28 | 50 | 74 | 1 |  |  |  |  |  |  |  |  |
| Indicate for each of the following treatment options, if you agree that it should be the first choice treatment in patients with anastomotic leak and overall conduit ischemia/necrosis. | | | | | | | | |  |  |  |  |  |  |  |  |
| Primary esophageal diversion | 1 | 5 | 4 | 12 | 39 | 42 | 79 | 3 |  |  |  |  |  |  |  |  |
| Continuity-preserving treatment | 16 | 27 | 13 | 18 | 18 | 9 | 27 | 5 |  |  |  |  |  |  |  |  |
| **Statement** | **Strongly disagree (n)** | **Disagree (n)** | **Somewhat disagree (n)** | **Somewhat agree**  **(n)** | **Agree**  **(n)** | **Strongly agree**  **(n)** | **Consensus (%)** | **No comment (n)** |  |  |  |  |  |  |  |  |
| Indicate for each of the following drainage tools if you agree that it is useful in patients with a cervical leak. | | | | | | | | |  |  |  |  |  |  |  |  |
| Bedside opening of the cervical incision | 2 | 3 | 4 | 11 | 37 | 46 | 81 | 3 |  |  |  |  |  |  |  |  |
| Nasogastric drainage tube | 3 | 9 | 8 | 19 | 48 | 16 | 62 | 3 |  |  |  |  |  |  |  |  |
| Surgical drainage tube | 1 | 9 | 18 | 25 | 39 | 11 | 49 | 3 |  |  |  |  |  |  |  |  |
| Radiological drainage tube | 1 | 19 | 21 | 22 | 29 | 10 | 38 | 4 |  |  |  |  |  |  |  |  |
| Endoscopic nasomediastinum drainage | 2 | 25 | 14 | 22 | 23 | 6 | 32 | 14 |  |  |  |  |  |  |  |  |
| Endoscopic vacuum therapy | 2 | 14 | 15 | 18 | 31 | 18 | 50 | 8 |  |  |  |  |  |  |  |  |
| Indicate for each of the following leak closure interventions if you agree that it is useful in patients with a cervical leak. | | | | | | | | |  |  |  |  |  |  |  |  |
| Stent placement (endoscopically or radiologically) | 13 | 22 | 23 | 23 | 14 | 6 | 20 | 5 |  |  |  |  |  |  |  |  |
| Endoscopic clipping | 9 | 31 | 22 | 19 | 15 | 0 | 16 | 10 |  |  |  |  |  |  |  |  |
| Endoscopic suturing | 11 | 35 | 22 | 15 | 5 | 0 | 6 | 18 |  |  |  |  |  |  |  |  |
| Endoscopic tissue adhesives | 10 | 38 | 16 | 15 | 10 | 1 | 12 | 16 |  |  |  |  |  |  |  |  |
| Endoscopic vacuum therapy combined with stent (e.g. VACStent) | 7 | 15 | 13 | 19 | 21 | 16 | 41 | 15 |  |  |  |  |  |  |  |  |
| Surgical closure | 8 | 39 | 22 | 26 | 5 | 2 | 7 | 4 |  |  |  |  |  |  |  |  |
| Indicate for each of the following drainage tools if you agree that it is useful in patients with an intrathoracic leak. | | | | | | | | |  |  |  |  |  |  |  |  |
| Nasogastric drainage tube | 1 | 4 | 2 | 20 | 49 | 28 | 74 | 2 |  |  |  |  |  |  |  |  |
| Surgical drainage tube | 0 | 4 | 9 | 16 | 50 | 25 | 72 | 2 |  |  |  |  |  |  |  |  |
| Radiological drainage tube | 0 | 3 | 3 | 24 | 44 | 28 | 71 | 4 |  |  |  |  |  |  |  |  |
| Endoscopic nasomediastinum drainage | 1 | 8 | 8 | 20 | 37 | 16 | 59 | 16 |  |  |  |  |  |  |  |  |
| Endoscopic vacuum therapy | 0 | 1 | 2 | 15 | 32 | 49 | 82 | 7 |  |  |  |  |  |  |  |  |
| Indicate for each of the following leak closure interventions if you agree that it is useful in patients with an intrathoracic leak. | | | | | | | | |  |  |  |  |  |  |  |  |
| Stent placement (endoscopically or radiologically) | 2 | 5 | 7 | 16 | 32 | 42 | 71 | 2 |  |  |  |  |  |  |  |  |
| Endoscopic clipping | 7 | 23 | 28 | 13 | 21 | 7 | 28 | 7 |  |  |  |  |  |  |  |  |
| Endoscopic suturing | 9 | 29 | 25 | 13 | 13 | 2 | 17 | 15 |  |  |  |  |  |  |  |  |
| **Statement** | **Strongly disagree (n)** | **Disagree (n)** | **Somewhat disagree (n)** | **Somewhat agree**  **(n)** | **Agree**  **(n)** | **Strongly agree**  **(n)** | **Consensus (%)** | **No comment (n)** |  |  |  |  |  |  |  |  |
| Endoscopic tissue adhesives | 9 | 31 | 29 | 11 | 12 | 3 | 16 | 11 |  |  |  |  |  |  |  |  |
| Endoscopic vacuum therapy combined with stent (e.g. VACStent) | 2 | 3 | 2 | 14 | 31 | 40 | 77 | 14 |  |  |  |  |  |  |  |  |
| Surgical closure | 3 | 25 | 29 | 20 | 18 | 6 | 24 | 5 |  |  |  |  |  |  |  |  |
| Indicate for each of the following situations if you agree that it is reason for surgical reintervention. | | | | | | | | |  |  |  |  |  |  |  |  |
| When the patient with a leak shows signs of uncontrolled sepsis | 0 | 4 | 3 | 9 | 36 | 54 | 85 | 0 |  |  |  |  |  |  |  |  |
| Step-up after failure of initiated treatment of anastomotic leak | 0 | 1 | 5 | 18 | 47 | 34 | 77 | 1 |  |  |  |  |  |  |  |  |
| Early leakage (within the first 72 hours after esophagectomy) | 1 | 17 | 14 | 30 | 26 | 17 | 41 | 1 |  |  |  |  |  |  |  |  |
| Uncontained intrathoracic anastomotic leaks | 1 | 8 | 13 | 14 | 32 | 36 | 65 | 2 |  |  |  |  |  |  |  |  |
| *POD; postoperative day. CRP; C-reactive protein. CT; computed tomography. EVT; endoscopic vacuum therapy. ^†^Defined as % respondents that agreed or strongly agreed with the statement.* | | | | | | | | |  |  |  |  |  |  |  |  |

| **Supplementary Table S4** Second-round Delphi survey results and corresponding consensus | | | | |  |  |  |  |  |  |  |  |  |  |  |  |
| --- | --- | --- | --- | --- | --- | --- | --- | --- | --- | --- | --- | --- | --- | --- | --- | --- |
| **Statements** | **Strongly disagree (n)** | **Disagree (n)** | **Somewhat disagree (n)** | **Somewhat agree**  **(n)** | **Agree**  **(n)** | **Strongly agree**  **(n)** | **Consensus^†^ (%)** | **No comment (n)** |  |  |  |  |  |  |  |  |
|  |  |  |  |  |  |  |  |  |  |  |  |  |  |  |  |  |
| *Diagnosis* | | | | | | | | |  |  |  |  |  |  |  |  |
| Indicate for each clinical sign if you agree that that clinical sign, amongst other signs and biochemical results, is useful in the diagnostic process of anastomotic leak after esophagectomy. | | | | | | | | |  |  |  |  |  |  |  |  |
| Tachycardia | 2 | 0 | 1 | 19 | 57 | 57 | 84 | 0 |  |  |  |  |  |  |  |  |
| Atrial fibrillation (Altered) | 2 | 6 | 7 | 30 | 54 | 33 | 66 | 4 |  |  |  |  |  |  |  |  |
| Subcutaneous emphysema | 2 | 15 | 21 | 51 | 30 | 17 | 35 | 0 |  |  |  |  |  |  |  |  |
| Chest pain | 3 | 14 | 20 | 59 | 33 | 5 | 28 | 2 |  |  |  |  |  |  |  |  |
| Redness and swelling in the associated neck incision (cervical anastomosis) (altered) | 2 | 0 | 4 | 11 | 48 | 69 | 87 | 2 |  |  |  |  |  |  |  |  |
| Increased output of postoperative drain | 3 | 16 | 16 | 46 | 41 | 14 | 40 | 0 |  |  |  |  |  |  |  |  |
| Indicate for each biochemical test if you agree that that biochemical test, amongst other signs and biochemical tests, is useful in the diagnostic process of anastomotic leak after esophagectomy. | | | | | | | | |  |  |  |  |  |  |  |  |
| Drain amylase | 3 | 10 | 11 | 25 | 49 | 32 | 62 | 6 |  |  |  |  |  |  |  |  |
| Serum white blood count (WBC) | 2 | 1 | 3 | 17 | 84 | 29 | 83 | 0 |  |  |  |  |  |  |  |  |
| Serum procalcitonin (PCT) | 2 | 9 | 16 | 28 | 47 | 11 | 51 | 23 |  |  |  |  |  |  |  |  |
| Serum neutrophil count | 1 | 10 | 12 | 42 | 51 | 11 | 49 | 9 |  |  |  |  |  |  |  |  |
| Serum bilirubin | 30 | 53 | 27 | 14 | 4 | 1 | 4 | 7 |  |  |  |  |  |  |  |  |
| Serum lactate | 8 | 37 | 30 | 38 | 17 | 3 | 15 | 3 |  |  |  |  |  |  |  |  |
| Routine, daily measurement of CRP is useful as part of standard postoperative protocol after esophagectomy. (New) | 2 | 14 | 14 | 20 | 35 | 46 | 62 | 5 |  |  |  |  |  |  |  |  |
| Routine measurement of CRP on alternate days is useful as part of standard postoperative protocol after esophagectomy. (New) | 0 | 14 | 9 | 36 | 42 | 29 | 55 | 6 |  |  |  |  |  |  |  |  |
| **Statements** | **Strongly disagree (n)** | **Disagree (n)** | **Somewhat disagree (n)** | **Somewhat agree**  **(n)** | **Agree**  **(n)** | **Strongly agree**  **(n)** | **Consensus (%)** | **No comment (n)** |  |  |  |  |  |  |  |  |
| If routine monitoring of CRP is performed, indicate for each postoperative day (POD) if you agree that routine monitoring should start on that POD. (New) | | | | | | | | |  |  |  |  |  |  |  |  |
| POD 1 | 16 | 26 | 11 | 14 | 27 | 32 | 47 | 10 |  |  |  |  |  |  |  |  |
| POD 2 | 12 | 22 | 13 | 20 | 31 | 21 | 44 | 17 |  |  |  |  |  |  |  |  |
| POD 3 | 4 | 14 | 5 | 9 | 43 | 49 | 74 | 12 |  |  |  |  |  |  |  |  |
| A CRP of 200mg/L, combined with other clinical signs and biochemical tests, is an useful cut-off value for considering additional diagnostics (e.g., CT scan, endoscopy). (New) | 1 | 6 | 13 | 26 | 51 | 31 | 64 | 8 |  |  |  |  |  |  |  |  |
| Indicate for each postoperative day (POD) if you agree that routine monitoring drain amylase on that day is useful in the diagnostic process of anastomotic leak. | | | | | | | | |  |  |  |  |  |  |  |  |
| POD 2 | 13 | 38 | 24 | 14 | 18 | 5 | 21 | 24 |  |  |  |  |  |  |  |  |
| POD 3 | 7 | 28 | 21 | 19 | 23 | 15 | 34 | 23 |  |  |  |  |  |  |  |  |
| POD 4 | 7 | 23 | 18 | 23 | 22 | 19 | 37 | 24 |  |  |  |  |  |  |  |  |
| POD 5 | 6 | 19 | 14 | 9 | 41 | 27 | 59 | 20 |  |  |  |  |  |  |  |  |
| POD 6 | 7 | 21 | 18 | 14 | 35 | 18 | 47 | 23 |  |  |  |  |  |  |  |  |
| POD 7 | 8 | 20 | 14 | 15 | 38 | 18 | 50 | 23 |  |  |  |  |  |  |  |  |
| The trend in drain amylase values is useful in the decision for additional diagnostics regarding anastomotic leak. | 6 | 20 | 23 | 30 | 28 | 12 | 34 | 17 |  |  |  |  |  |  |  |  |
| Measuring drain amylase levels is useful when suspecting anastomotic leak, based on suspicious drain fluid aspect. (New) | 5 | 14 | 13 | 28 | 37 | 28 | 52 | 11 |  |  |  |  |  |  |  |  |
| Measuring drain amylase levels is useful when suspecting anastomotic leak, based on clinical signs and/or biochemical results (e.g. elevated CRP), without suspicious drain fluid aspect. (New) | 3 | 13 | 14 | 32 | 40 | 24 | 51 | 10 |  |  |  |  |  |  |  |  |
| **Statements** | **Strongly disagree (n)** | **Disagree (n)** | **Somewhat disagree (n)** | **Somewhat agree**  **(n)** | **Agree**  **(n)** | **Strongly agree**  **(n)** | **Consensus (%)** | **No comment (n)** |  |  |  |  |  |  |  |  |
| An amylase of 500mg/L, combined with other clinical signs and biochemical tests, is an useful cut-off value for considering additional diagnostics (e.g. CT, endoscopy). (New) | 5 | 9 | 15 | 23 | 44 | 22 | 56 | 18 |  |  |  |  |  |  |  |  |
| Screening for anastomotic leak using additional diagnostics (e.g. CT, endoscopy, radiography) should be performed in asymptomatic patients with normal biochemical results (Altered). | 37 | 42 | 14 | 9 | 20 | 14 | 25 | 0 |  |  |  |  |  |  |  |  |
| Screening for anastomotic leak in asymptomatic patients and normal biochemical results should be performed by …. (Altered) | | | | | | | | |  |  |  |  |  |  |  |  |
| Routine contrast radiography | 31 | 46 | 4 | 11 | 28 | 10 | 29 | 6 |  |  |  |  |  |  |  |  |
| Routine computed tomography (CT) scan | 38 | 54 | 8 | 13 | 10 | 7 | 71^‡^ | 6 |  |  |  |  |  |  |  |  |
| Routine endoscopy | 52 | 56 | 9 | 9 | 3 | 0 | 84^‡^ | 7 |  |  |  |  |  |  |  |  |
| Methylene blue is useful when drains are still in place and you suspect cervical anastomotic leak based on clinical signs and/or biochemical results. (New) | 4 | 11 | 11 | 33 | 46 | 26 | 55 | 5 |  |  |  |  |  |  |  |  |
| If you suspect cervical anastomotic leak based on clinical signs and/or biochemical tests, indicate if you agree that bedside opening of the cervical incision is indicated in the following scenarios. (New) | | | | | | | | |  |  |  |  |  |  |  |  |
| Always | 12 | 32 | 23 | 26 | 20 | 19 | 30 | 4 |  |  |  |  |  |  |  |  |
| Local signs of infection (red/swollen) | 1 | 8 | 6 | 19 | 41 | 58 | 74 | 3 |  |  |  |  |  |  |  |  |
| No drains in place | 5 | 17 | 10 | 25 | 44 | 27 | 56 | 8 |  |  |  |  |  |  |  |  |
| The patient is septic | 3 | 9 | 8 | 12 | 49 | 52 | 76 | 3 |  |  |  |  |  |  |  |  |
| A patient with a cervical anastomosis is suspected of anastomotic leak based on systemic signs and/or biochemical tests without local sings of infection. The next diagnostic assessment to be performed is … (New) | | | | | | | | |  |  |  |  |  |  |  |  |
| Bedside opening of the cervical incision | 19 | 34 | 16 | 32 | 20 | 10 | 23 | 5 |  |  |  |  |  |  |  |  |
| Contrast radiography | 10 | 32 | 12 | 16 | 39 | 22 | 47 | 5 |  |  |  |  |  |  |  |  |
| Computed tomography (CT) scan | 2 | 6 | 5 | 13 | 46 | 60 | 80 | 4 |  |  |  |  |  |  |  |  |
| **Statements** | **Strongly disagree (n)** | **Disagree (n)** | **Somewhat disagree (n)** | **Somewhat agree**  **(n)** | **Agree**  **(n)** | **Strongly agree**  **(n)** | **Consensus (%)** | **No comment (n)** |  |  |  |  |  |  |  |  |
| Endoscopy | 5 | 16 | 21 | 35 | 37 | 18 | 42 | 4 |  |  |  |  |  |  |  |  |
| CT scan and endoscopy | 3 | 9 | 20 | 27 | 35 | 35 | 54 | 7 |  |  |  |  |  |  |  |  |
| A patient with a cervical anastomosis is suspected of anastomotic leak based on systemic signs and/or biochemical tests and the cervical incision shows local sings of infection (red/swollen). The next diagnostic assessment to be performed is … (New) | | | | | | | | |  |  |  |  |  |  |  |  |
| Bedside opening of the cervical incision | 3 | 11 | 5 | 18 | 34 | 61 | 72 | 4 |  |  |  |  |  |  |  |  |
| Contrast radiography | 8 | 38 | 16 | 19 | 38 | 11 | 38 | 6 |  |  |  |  |  |  |  |  |
| Computed tomography (CT) scan | 2 | 6 | 5 | 17 | 54 | 47 | 77 | 5 |  |  |  |  |  |  |  |  |
| Endoscopy | 8 | 12 | 24 | 29 | 39 | 18 | 44 | 6 |  |  |  |  |  |  |  |  |
| CT scan and endoscopy | 7 | 10 | 18 | 24 | 37 | 32 | 54 | 8 |  |  |  |  |  |  |  |  |
| If you confirmed cervical anastomotic leak by opening the cervical incision and patient has mild symptoms (not septic), indicate for the following diagnostic assessments if you agree that it is useful to be performed next. (New) | | | | | | | | |  |  |  |  |  |  |  |  |
| Contrast radiography | 11 | 49 | 16 | 23 | 20 | 9 | 23 | 8 |  |  |  |  |  |  |  |  |
| Computed tomography (CT) scan | 2 | 10 | 10 | 19 | 47 | 42 | 69 | 6 |  |  |  |  |  |  |  |  |
| Endoscopy | 0 | 21 | 25 | 29 | 35 | 18 | 41 | 8 |  |  |  |  |  |  |  |  |
| CT scan and endoscopy | 7 | 21 | 19 | 22 | 30 | 27 | 45 | 10 |  |  |  |  |  |  |  |  |
| If you confirmed cervical anastomotic leak by opening the cervical incision and patient is septic, indicate for the following diagnostic assessment if you agree that it is useful to be performed next. (New) | | | | | | | | |  |  |  |  |  |  |  |  |
| Contrast radiography | 18 | 55 | 16 | 17 | 18 | 5 | 18 | 7 |  |  |  |  |  |  |  |  |
| Computed tomography (CT) scan | 1 | 4 | 3 | 7 | 44 | 71 | 89 | 6 |  |  |  |  |  |  |  |  |
| Endoscopy | 3 | 16 | 11 | 20 | 50 | 28 | 61 | 8 |  |  |  |  |  |  |  |  |
| CT scan and endoscopy | 3 | 11 | 9 | 16 | 32 | 55 | 69 | 10 |  |  |  |  |  |  |  |  |
| Methylene blue is useful when drains are still in place and you suspect intrathoracic anastomotic leak based on clinical signs and/or biochemical results. (New) | 5 | 23 | 12 | 29 | 40 | 20 | 47 | 7 |  |  |  |  |  |  |  |  |
| **Statements** | **Strongly disagree (n)** | **Disagree (n)** | **Somewhat disagree (n)** | **Somewhat agree**  **(n)** | **Agree**  **(n)** | **Strongly agree**  **(n)** | **Consensus (%)** | **No comment (n)** |  |  |  |  |  |  |  |  |
| When a cervical or intrathoracic anastomotic leak is diagnosed on CT scan, it is useful to perform additional endoscopy. (Altered) | 1 | 1 | 5 | 23 | 43 | 60 | 77 | 3 |  |  |  |  |  |  |  |  |
| The size of the defect should be measured during endoscopy in order to guide anastomotic leak treatment. | 0 | 3 | 1 | 16 | 52 | 63 | 85 | 1 |  |  |  |  |  |  |  |  |
| The condition of the gastric conduit (e.g. presence of conduit ischemia/necrosis) should be assessed during endoscopy in order to guide anastomotic leak treatment. (New) | 0 | 0 | 1 | 2 | 38 | 95 | 98 | 0 |  |  |  |  |  |  |  |  |
| The extent of contamination (e.g. mediastinal and/or pleural fluid collections) should be assessed on CT in order to guide anastomotic leak treatment. (New) | 0 | 0 | 0 | 2 | 27 | 107 | 99 | 0 |  |  |  |  |  |  |  |  |
| The individual risk of 90-day mortality as predicted by a ‘leak severity score’ is useful to guide anastomotic leak treatment. | 2 | 8 | 20 | 58 | 31 | 9 | 31 | 8 |  |  |  |  |  |  |  |  |
| *Treatment* | | | | | | | | |  |  |  |  |  |  |  |  |
| Indicate for each of the following treatment options if you agree that it should be part of non-invasive management/supportive care of all anastomotic leak patients. | | | | | | | | |  |  |  |  |  |  |  |  |
| Antifungal therapy (according to local protocol) | 1 | 6 | 9 | 19 | 68 | 33 | 74 | 0 |  |  |  |  |  |  |  |  |
| Nil-by-mouth | 0 | 6 | 6 | 10 | 49 | 64 | 84 | 1 |  |  |  |  |  |  |  |  |
| Clear water diet | 12 | 44 | 24 | 24 | 23 | 9 | 24 | 0 |  |  |  |  |  |  |  |  |
| Antacids | 4 | 34 | 24 | 26 | 29 | 13 | 32 | 6 |  |  |  |  |  |  |  |  |
| Proton-pump inhibitors (New) | 0 | 9 | 4 | 16 | 55 | 51 | 79 | 1 |  |  |  |  |  |  |  |  |
| **Statements** | **Strongly disagree (n)** | **Disagree (n)** | **Somewhat disagree (n)** | **Somewhat agree**  **(n)** | **Agree**  **(n)** | **Strongly agree**  **(n)** | **Consensus (%)** | **No comment (n)** |  |  |  |  |  |  |  |  |
| The next statements are formulated regarding patients with a cervical leak with only local manifestations without fluid collections and mild symptoms. Indicate for each of the following statements if you agree with that statement. (Altered) | | | | | | | | |  |  |  |  |  |  |  |  |
| Only non-invasive management/supportive care should be performed | 2 | 20 | 26 | 21 | 35 | 28 | 48 | 4 |  |  |  |  |  |  |  |  |
| Additional drainage interventions should be performed (e.g. opening cervical incision, EVT, drainage tube) | 2 | 11 | 20 | 25 | 44 | 30 | 56 | 4 |  |  |  |  |  |  |  |  |
| Additional leak closure interventions should be performed (e.g. stent, endoscopic tissue adhesives, surgical closure) | 12 | 38 | 41 | 21 | 16 | 3 | 15 | 5 |  |  |  |  |  |  |  |  |
| The next statements are formulated regarding patients with a cervical leak without conduit ischemia/necrosis. Indicate for each scenario if, in addition to supportive care and drainage interventions, leak closure interventions (e.g. stent, EVT, surgical closure) should be performed when … (New) | | | | | | | | |  |  |  |  |  |  |  |  |
| The patient has mild symptoms and mediastinal fluid collections | 3 | 17 | 14 | 28 | 48 | 20 | 52 | 6 |  |  |  |  |  |  |  |  |
| The patient is septic and has mediastinal fluid collections | 4 | 16 | 9 | 10 | 34 | 57 | 70 | 6 |  |  |  |  |  |  |  |  |
| The patient has mild symptoms and pleural fluid collections | 2 | 20 | 8 | 27 | 49 | 24 | 56 | 6 |  |  |  |  |  |  |  |  |
| The patient is septic and has pleural fluid collections | 4 | 16 | 9 | 11 | 30 | 60 | 69 | 6 |  |  |  |  |  |  |  |  |
| Indicate for each of the following drainage tools if you agree that it is useful in patients with a cervical leak. | | | | | | | | |  |  |  |  |  |  |  |  |
| Nasogastric drainage tube | 3 | 14 | 22 | 35 | 43 | 16 | 44 | 3 |  |  |  |  |  |  |  |  |
| Surgical drainage tube | 1 | 14 | 20 | 51 | 39 | 8 | 35 | 3 |  |  |  |  |  |  |  |  |
| Radiological drainage tube | 2 | 22 | 30 | 50 | 23 | 6 | 22 | 3 |  |  |  |  |  |  |  |  |
| Endoscopic nasomediastinum drainage | 2 | 32 | 35 | 32 | 24 | 3 | 21 | 8 |  |  |  |  |  |  |  |  |
| Endoscopic vacuum therapy (EVT) | 2 | 8 | 15 | 35 | 51 | 21 | 55 | 4 |  |  |  |  |  |  |  |  |
| Surgical drainage (New) | 0 | 9 | 17 | 41 | 55 | 11 | 50 | 3 |  |  |  |  |  |  |  |  |
| **Statements** | **Strongly disagree (n)** | **Disagree (n)** | **Somewhat disagree (n)** | **Somewhat agree**  **(n)** | **Agree**  **(n)** | **Strongly agree**  **(n)** | **Consensus (%)** | **No comment (n)** |  |  |  |  |  |  |  |  |
| Indicate for each of the following leak closure interventions if you agree that it is useful in patients with a cervical leak. | | | | | | | | |  |  |  |  |  |  |  |  |
| Stent placement (endoscopically or radiologically) | 16 | 55 | 23 | 19 | 14 | 4 | 14 | 5 |  |  |  |  |  |  |  |  |
| Endoscopic clipping | 15 | 61 | 24 | 21 | 7 | 2 | 7 | 6 |  |  |  |  |  |  |  |  |
| Endoscopic suturing | 23 | 73 | 18 | 8 | 4 | 1 | 76**^‡^** | 9 |  |  |  |  |  |  |  |  |
| Endoscopic tissue adhesives | 23 | 60 | 22 | 21 | 2 | 0 | 2 | 8 |  |  |  |  |  |  |  |  |
| Endoscopic vacuum therapy (EVT) (New) | 3 | 14 | 11 | 37 | 49 | 16 | 50 | 6 |  |  |  |  |  |  |  |  |
| Endoscopic vacuum therapy combined with stent (e.g. VACStent) | 7 | 34 | 20 | 32 | 23 | 1 | 21 | 19 |  |  |  |  |  |  |  |  |
| Surgical closure | 26 | 57 | 25 | 15 | 7 | 1 | 6 | 5 |  |  |  |  |  |  |  |  |
| The next statements are formulated regarding patients with an intrathoracic leak with only local manifestations without fluid collections and mild symptoms. Indicate for each of the following statements if you agree with that statement. (Altered) | | | | | | | | |  |  |  |  |  |  |  |  |
| Only non-invasive management/supportive care should be performed | 3 | 23 | 24 | 25 | 45 | 15 | 44 | 1 |  |  |  |  |  |  |  |  |
| Additional drainage interventions should be performed (e.g. EVT, drainage tube) | 3 | 11 | 20 | 36 | 42 | 23 | 48 | 1 |  |  |  |  |  |  |  |  |
| Additional leak closure should be performed (e.g. stent, EVT, surgical closure) | 2 | 21 | 29 | 29 | 37 | 16 | 40 | 2 |  |  |  |  |  |  |  |  |
| The following statements are formulated regarding patients with an intrathoracic leak without conduit ischemia/necrosis. Indicate for each scenario if, in addition to supportive care and drainage interventions, leak closure interventions (e.g. stent, EVT, surgical closure) should be performed when … (New) | | | | | | | | |  |  |  |  |  |  |  |  |
| The patient has mild symptoms and mediastinal fluid collections | 1 | 11 | 9 | 23 | 59 | 30 | 67 | 3 |  |  |  |  |  |  |  |  |
| The patient is septic and mediastinal fluid collections | 2 | 12 | 2 | 7 | 43 | 67 | 83 | 3 |  |  |  |  |  |  |  |  |
| The patient has mild symptoms and pleural fluid collections | 1 | 10 | 5 | 14 | 61 | 42 | 77 | 3 |  |  |  |  |  |  |  |  |
| The patient is septic and has pleural fluid collections | 3 | 9 | 3 | 8 | 38 | 72 | 83 | 3 |  |  |  |  |  |  |  |  |
| **Statements** | **Strongly disagree (n)** | **Disagree (n)** | **Somewhat disagree (n)** | **Somewhat agree**  **(n)** | **Agree**  **(n)** | **Strongly agree**  **(n)** | **Consensus (%)** | **No comment (n)** |  |  |  |  |  |  |  |  |
| Indicate for each of the following drainage tools if you agree that it is useful in patients with an intrathoracic leak. | | | | | | | | |  |  |  |  |  |  |  |  |
| Nasogastric drainage tube | 0 | 4 | 9 | 20 | 65 | 37 | 76 | 1 |  |  |  |  |  |  |  |  |
| Surgical drainage tube | 0 | 3 | 6 | 20 | 83 | 23 | 79 | 1 |  |  |  |  |  |  |  |  |
| Radiological drainage tube | 0 | 0 | 3 | 23 | 79 | 29 | 81 | 2 |  |  |  |  |  |  |  |  |
| Endoscopic nasomediastinum drainage | 0 | 15 | 24 | 34 | 45 | 12 | 44 | 6 |  |  |  |  |  |  |  |  |
| Surgical drainage (New) | 0 | 9 | 25 | 35 | 55 | 11 | 49 | 1 |  |  |  |  |  |  |  |  |
| Indicate for each of the following leak closure interventions if you agree that it is useful in patients with an intrathoracic leak. | | | | | | | | |  |  |  |  |  |  |  |  |
| Stent placement (endoscopically or radiologically) | 3 | 4 | 11 | 29 | 56 | 31 | 65 | 2 |  |  |  |  |  |  |  |  |
| Endoscopic clipping | 8 | 38 | 34 | 30 | 19 | 4 | 17 | 3 |  |  |  |  |  |  |  |  |
| Endoscopic suturing | 13 | 65 | 28 | 15 | 7 | 2 | 7 | 6 |  |  |  |  |  |  |  |  |
| Endoscopic tissue adhesives | 19 | 62 | 23 | 15 | 5 | 2 | 6 | 10 |  |  |  |  |  |  |  |  |
| Endoscopic vacuum therapy (EVT) (New) | 2 | 2 | 4 | 16 | 65 | 44 | 82 | 3 |  |  |  |  |  |  |  |  |
| Endoscopic vacuum therapy combined with stent (e.g. VACStent) | 3 | 4 | 5 | 20 | 63 | 23 | 73 | 18 |  |  |  |  |  |  |  |  |
| Surgical closure | 10 | 36 | 32 | 29 | 21 | 6 | 20 | 2 |  |  |  |  |  |  |  |  |
| Indicate for each of the following treatment options, if you agree that it should be the first choice treatment for patients with anastomotic leak and mild symptoms, and limited ischemia/necrosis of the top. (New) | | | | | | | | |  |  |  |  |  |  |  |  |
| Primary esophageal diversion | 23 | 44 | 35 | 19 | 8 | 4 | 9 | 3 |  |  |  |  |  |  |  |  |
| Continuity-preserving treatment | 1 | 3 | 2 | 15 | 68 | 46 | 84 | 1 |  |  |  |  |  |  |  |  |
| Indicate for each of the following treatment options, if you agree that it should be the first choice treatment for septic patients with anastomotic leak, and limited ischemia/necrosis of the top. (New) | | | | | | | | |  |  |  |  |  |  |  |  |
| Primary esophageal diversion | 12 | 33 | 32 | 21 | 22 | 15 | 27 | 1 |  |  |  |  |  |  |  |  |
| Continuity-preserving treatment | 4 | 15 | 17 | 17 | 55 | 27 | 61 | 1 |  |  |  |  |  |  |  |  |
| Indicate for each of the following treatment options, if you agree that it should be the first choice treatment for patients with anastomotic leak and mild symptoms, and overall ischemia/necrosis of the conduit. (New) | | | | | | | | |  |  |  |  |  |  |  |  |
| Primary esophageal diversion | 1 | 6 | 16 | 10 | 61 | 39 | 75 | 3 |  |  |  |  |  |  |  |  |
| **Statements** | **Strongly disagree (n)** | **Disagree (n)** | **Somewhat disagree (n)** | **Somewhat agree**  **(n)** | **Agree**  **(n)** | **Strongly agree**  **(n)** | **Consensus (%)** | **No comment (n)** |  |  |  |  |  |  |  |  |
| Continuity-preserving treatment | 15 | 52 | 23 | 18 | 19 | 7 | 19 | 2 |  |  |  |  |  |  |  |  |
| Indicate for each of the following treatment options, if you agree that it should be the first choice treatment for septic patients with anastomotic leak, and overall ischemia/necrosis of the conduit. (New) | | | | | | | | |  |  |  |  |  |  |  |  |
| Primary esophageal diversion | 0 | 4 | 2 | 9 | 27 | 91 | 89 | 3 |  |  |  |  |  |  |  |  |
| Continuity-preserving treatment | 52 | 49 | 17 | 9 | 3 | 4 | 75**^‡^** | 2 |  |  |  |  |  |  |  |  |
| Indicate for each of the following situations if you agree that it is reason for surgical washout and better drainage. (Altered) | | | | | | | | |  |  |  |  |  |  |  |  |
| When the patient with a leak shows signs of uncontrolled sepsis | 1 | 3 | 4 | 13 | 50 | 64 | 84 | 1 |  |  |  |  |  |  |  |  |
| Step-up after failure of initiated treatment of anastomotic leak | 0 | 3 | 2 | 19 | 64 | 47 | 82 | 1 |  |  |  |  |  |  |  |  |
| Early leakage (within the first 72 hours after esophagectomy) | 1 | 19 | 19 | 41 | 36 | 19 | 41 | 1 |  |  |  |  |  |  |  |  |
| Uncontained intrathoracic anastomotic leaks | 2 | 5 | 10 | 18 | 43 | 56 | 74 | 2 |  |  |  |  |  |  |  |  |
| Indicate for each of the following situations if you agree that it is reason for surgical repair of the anastomotic defect. (New) | | | | | | | | |  |  |  |  |  |  |  |  |
| When the patient with a leak shows signs of uncontrolled sepsis | 16 | 36 | 27 | 16 | 26 | 13 | 29 | 2 |  |  |  |  |  |  |  |  |
| Step-up after failure of initiated treatment of anastomotic leak | 10 | 28 | 26 | 23 | 36 | 11 | 35 | 2 |  |  |  |  |  |  |  |  |
| Early leakage (within the first 72 hours after esophagectomy) | 4 | 12 | 18 | 33 | 45 | 22 | 50 | 2 |  |  |  |  |  |  |  |  |
| Uncontained intrathoracic anastomotic leaks | 15 | 35 | 18 | 20 | 33 | 13 | 34 | 2 |  |  |  |  |  |  |  |  |
| Indicate for each of the following situations if you agree that it is reason for esophageal diversion. (New) | | | | | | | | |  |  |  |  |  |  |  |  |
| When the patient with a leak shows signs of uncontrolled sepsis | 2 | 18 | 19 | 19 | 45 | 33 | 57 | 0 |  |  |  |  |  |  |  |  |
| Step-up after failure of initiated treatment of anastomotic leak | 2 | 14 | 26 | 23 | 53 | 18 | 52 | 0 |  |  |  |  |  |  |  |  |
| Early leakage (within the first 72 hours after esophagectomy) | 18 | 48 | 38 | 18 | 10 | 3 | 10 | 1 |  |  |  |  |  |  |  |  |
| Uncontained intrathoracic anastomotic leaks | 8 | 26 | 20 | 30 | 34 | 17 | 38 | 1 |  |  |  |  |  |  |  |  |
| *CRP; C-reactive protein. POD; postoperative day. CT; computed tomography. ^†^Defined as % respondents that agreed or strongly agreed with the statement.*  ***^‡^****(Near) Consensus based on (strongly) disagreed.* | | | | | | | | |  |  |  |  |  |  |  |  |

| **Supplementary Table S5** First-round Delphi survey consensus stratified by hospital volume and continent | | | | | | | | | | |
| --- | --- | --- | --- | --- | --- | --- | --- | --- | --- | --- |
|  |  | **Hospital volume** | | | **Continent** | | | | | |
| **Statements** | **General** | **Low** | **Middle** | **High** | **Asia** | **Africa** | **North America** | **South America** | **Europe** | **Oceania** |
|  |  |  |  |  |  |  |  |  |  |  |
| Number of respondents (n) | 106 | 7 | 52 | 47 | 17 | 4 | 7 | 6 | 67 | 5 |
| *Diagnosis* | | | | | | | | | | |
| Indicate for each clinical sign if you agree that that clinical sign is useful in the diagnostic process of anastomotic leak after esophagectomy. | | | | | | | | | | |
| Tachycardia | 78 | 100 | 85 | 69 | 71 | 75 | 86 | 100 | 78 | 80 |
| Cardiac arrhythmia (e.g. atrial fibrillation) | 62 | 71 | 69 | 53 | 35 | 0 | 71 | 50 | 74 | 40 |
| Fever (>38.5◦C) | 85 | 86 | 83 | 87 | 82 | 75 | 71 | 67 | 91 | 60 |
| Respiratory distress (e.g. tachypnea, oxygen delivery increase) | 81 | 86 | 80 | 81 | 53 | 75 | 57 | 67 | 94 | 60 |
| Subcutaneous emphysema | 48 | 71 | 45 | 48 | 47 | 75 | 29 | 60 | 50 | 20 |
| Chest pain | 43 | 71 | 38 | 45 | 47 | 100 | 14 | 50 | 43 | 20 |
| Redness and swelling in the associated neck and chest incisions | 77 | 86 | 74 | 80 | 100 | 100 | 71 | 60 | 75 | 40 |
| Changed aspect of postoperative drain fluids | 92 | 100 | 92 | 91 | 88 | 100 | 100 | 100 | 92 | 75 |
| Increased output of postoperative drain | 50 | 86 | 48 | 46 | 53 | 100 | 71 | 67 | 44 | 20 |
| Indicate for each biochemical test if you agree that that biochemical test is useful in the diagnostic process of anastomotic leak after esophagectomy. | | | | | | | | | | |
| Serum C-reactive protein (CRP) | 90 | 86 | 88 | 92 | 88 | 100 | 50 | 83 | 93 | 100 |
| Drain amylase | 74 | 67 | 76 | 73 | 80 | 100 | 83 | 67 | 72 | 60 |
| Serum white blood count (WBC) | 75 | 86 | 69 | 79 | 65 | 100 | 71 | 67 | 76 | 80 |
| Serum CRP-to-albumin ratio | 34 | 40 | 46 | 20 | 25 | 100 | 33 | 80 | 29 | 25 |
| Serum procalcitonin | 52 | 86 | 60 | 35 | 69 | 100 | 0 | 20 | 50 | 33 |
| Serum prealbumin | 15 | 50 | 16 | 8 | 40 | 0 | 0 | 20 | 8 | 33 |
| Plasma cytokines | 13 | 33 | 20 | 5 | 25 | 0 | 0 | 0 | 13 | 0 |
| Serum neutrophil count | 49 | 57 | 52 | 44 | 53 | 100 | 50 | 50 | 42 | 75 |
| **Statements** | **General** | **Low** | **Middle** | **High** | **Asia** | **Africa** | **North America** | **South America** | **Europe** | **Oceania** |
| Serum platelet-to-lymphocyte ratio | 24 | 50 | 33 | 9 | 11 | 0 | 0 | 60 | 24 | 33 |
| Neutrophil-to-lymphocyte ratio | 27 | 40 | 38 | 12 | 36 | 0 | 0 | 50 | 22 | 33 |
| Serum bilirubin | 2 | 17 | 0 | 3 | 8 | 0 | 0 | 0 | 2 | 0 |
| The coefficient of serum procalcitonin and blood G | 6 | 25 | 9 | 0 | 14 | 0 | 0 | 25 | 3 | 0 |
| Serum lactate | 36 | 71 | 41 | 23 | 33 | 0 | 20 | 67 | 34 | 50 |
| Indicate for each postoperative day (POD) if you agree that monitoring serum C-reactive protein (CRP) on that day is useful in the diagnostic process of anastomotic leak. | | | | | | | | | | |
| POD 1 | 32 | 17 | 25 | 41 | 20 | 25 | 0 | 0 | 38 | 40 |
| POD 2 | 33 | 33 | 32 | 33 | 20 | 33 | 0 | 0 | 40 | 20 |
| POD 3 | 64 | 50 | 70 | 60 | 50 | 75 | 0 | 50 | 68 | 80 |
| POD 4 | 68 | 57 | 67 | 71 | 73 | 67 | 0 | 60 | 72 | 40 |
| POD 5 | 86 | 71 | 87 | 87 | 88 | 75 | 0 | 80 | 88 | 100 |
| POD 6 | 76 | 86 | 73 | 79 | 87 | 67 | 100 | 60 | 78 | 40 |
| POD 7 | 72 | 86 | 69 | 73 | 87 | 67 | 100 | 40 | 70 | 80 |
| POD 8 | 55 | 67 | 50 | 59 | 79 | 67 | 50 | 20 | 54 | 40 |
| POD 9 or longer | 44 | 67 | 38 | 46 | 67 | 67 | 50 | 20 | 41 | 20 |
| The trend in CRP values is useful in the decision for additional diagnostics regarding anastomotic leak. | 89 | 86 | 89 | 89 | 86 | 75 | 67 | 80 | 91 | 100 |
| Indicate for each postoperative day (POD) if you agree that monitoring drain amylase on that day is useful in the diagnostic process of anastomotic leak. | | | | | | | | | | |
| POD 1 | 20 | 0 | 18 | 25 | 27 | 33 | 33 | 20 | 18 | 0 |
| POD 2 | 29 | 20 | 29 | 30 | 27 | 67 | 33 | 20 | 30 | 0 |
| POD 3 | 49 | 40 | 51 | 47 | 58 | 100 | 67 | 33 | 47 | 20 |
| POD 4 | 60 | 100 | 58 | 55 | 55 | 100 | 100 | 100 | 52 | 40 |
| POD 5 | 65 | 100 | 69 | 55 | 73 | 100 | 100 | 100 | 57 | 40 |
| POD 6 | 58 | 80 | 61 | 52 | 78 | 67 | 100 | 100 | 48 | 40 |
| POD 7 | 56 | 80 | 59 | 50 | 80 | 67 | 67 | 83 | 48 | 40 |
| POD 8 | 49 | 80 | 47 | 47 | 78 | 67 | 33 | 80 | 38 | 60 |
| **Statements** | **General** | **Low** | **Middle** | **High** | **Asia** | **Africa** | **North America** | **South America** | **Europe** | **Oceania** |
| POD 9 or longer | 45 | 80 | 40 | 45 | 89 | 67 | 33 | 60 | 33 | 40 |
| The trend in drain amylase values is useful in the decision for additional diagnostics regarding anastomotic leak. | 47 | 100 | 41 | 49 | 70 | 67 | 50 | 80 | 41 | 20 |
| Screening for anastomotic leak in asymptomatic patients should be performed by …. | | | | | | | | | | |
| Routine contrast radiography | 36 | 43 | 44 | 25 | 50 | 67 | 71 | 67 | 22 | 60 |
| Routine computed tomography (CT) scan | 21 | 29 | 20 | 21 | 25 | 0 | 0 | 67 | 16 | 60 |
| Routine endoscopy | 9 | 14 | 4 | 14 | 19 | 0 | 0 | 17 | 8 | 0 |
| Screening in asymptomatic patients should not be performed | 50 | 29 | 47 | 57 | 65 | 33 | 17 | 17 | 54 | 40 |
| If you suspect cervical anastomotic leak based on clinical sign(s) and/or biochemical test(s), indicate for the following diagnostic assessments if you agree that it should be the first choice. | | | | | | | | | | |
| Bedside opening of the cervical incision | 61 | 71 | 52 | 71 | 89 | 50 | 71 | 67 | 56 | 20 |
| Contrast radiography | 36 | 29 | 40 | 33 | 44 | 67 | 71 | 83 | 21 | 80 |
| Computed tomography (CT) scan | 76 | 86 | 72 | 79 | 77 | 0 | 57 | 100 | 81 | 60 |
| Endoscopy | 43 | 29 | 42 | 47 | 59 | 0 | 29 | 33 | 45 | 20 |
| CT scan and endoscopy | 49 | 29 | 51 | 50 | 53 | 0 | 29 | 67 | 53 | 20 |
| If you suspect intrathoracic anastomotic leak based on clinical sign(s) and/or biochemical test(s), indicate for the following diagnostic assessments if you agree that it should be the first choice. | | | | | | | | | | |
| Contrast radiography | 38 | 29 | 38 | 39 | 56 | 67 | 57 | 83 | 24 | 60 |
| Computed tomography (CT) scan | 84 | 86 | 90 | 78 | 100 | 67 | 86 | 83 | 79 | 100 |
| Endoscopy | 61 | 29 | 63 | 64 | 77 | 67 | 57 | 67 | 58 | 40 |
| CT scan and endoscopy | 71 | 43 | 76 | 70 | 71 | 33 | 57 | 67 | 76 | 60 |
| When an anastomotic leak is diagnosed on CT scan, it is useful to perform additional endoscopy. | 77 | 86 | 75 | 78 | 75 | 25 | 86 | 67 | 81 | 80 |
| When an anastomotic leak is diagnosed on endoscopy, it is useful to perform additional CT scan. | 90 | 100 | 90 | 87 | 88 | 100 | 71 | 100 | 90 | 100 |
| **Statements** | **General** | **Low** | **Middle** | **High** | **Asia** | **Africa** | **North America** | **South America** | **Europe** | **Oceania** |
| The size of the defect should be measured during endoscopy in order to guide anastomotic leak treatment. | 78 | 100 | 75 | 79 | 88 | 50 | 71 | 100 | 78 | 60 |
| A ‘leak severity score’ predicting the risk of 90-day mortality is useful to guide anastomotic leak treatment. | 50 | 67 | 50 | 48 | 58 | 67 | 29 | 80 | 47 | 60 |
| *Treatment* | | | | | | | | | | |
| Indicate for each of the following treatment options if you agree that it should be part of non-invasive management/supportive care of all anastomotic leak patients. | | | | | | | | | | |
| Broad spectrum antibiotics (according to local protocol) | 99 | 100 | 100 | 98 | 94 | 100 | 100 | 100 | 100 | 100 |
| Antifungal therapy (according to local protocol) | 69 | 71 | 69 | 68 | 47 | 33 | 57 | 50 | 78 | 80 |
| Nil-by-mouth | 79 | 71 | 85 | 75 | 82 | 75 | 71 | 83 | 78 | 100 |
| Clear water diet | 24 | 29 | 22 | 26 | 24 | 33 | 0 | 17 | 28 | 0 |
| Feeding support (e.g. enteral feeding using jejunal tube placement, jejunostomy or parenteral feeding) | 98 | 100 | 98 | 98 | 88 | 100 | 100 | 100 | 100 | 100 |
| Antacids | 65 | 71 | 56 | 74 | 63 | 33 | 43 | 40 | 74 | 40 |
| The next statements are formulated regarding patients with cervical leak with only local manifestations and no fluid collections. Indicate for each of the following statements if you agree with that statement. | | | | | | | | | | |
| Only non-invasive management/supportive care should be performed | 51 | 43 | 52 | 51 | 53 | 75 | 29 | 67 | 49 | 60 |
| Additional drainage interventions should be performed (e.g. opening cervical incision, EVT, drainage tube) | 63 | 71 | 64 | 60 | 71 | 50 | 57 | 33 | 69 | 0 |
| Additional leak closure interventions should be performed (e.g. stent, endoscopic tissue adhesives, surgical closure) | 13 | 0 | 10 | 18 | 19 | 0 | 29 | 17 | 11 | 0 |
| **Statements** | **General** | **Low** | **Middle** | **High** | **Asia** | **Africa** | **North America** | **South America** | **Europe** | **Oceania** |
| The next statements are formulated regarding patients with cervical leak with mediastinal fluid collections. Indicate for each of the following statements if you agree with that statement. | | | | | | | | | | |
| Only non-invasive management/supportive care should be performed | 7 | 14 | 4 | 9 | 13 | 0 | 0 | 17 | 5 | 20 |
| Additional drainage interventions should be performed (e.g. opening cervical incision, EVT, drainage tube) | 91 | 86 | 87 | 98 | 75 | 100 | 86 | 83 | 97 | 80 |
| Additional leak closure interventions should be performed (e.g. stent, endoscopic tissue adhesives, surgical closure) | 45 | 57 | 48 | 40 | 56 | 50 | 71 | 83 | 36 | 40 |
| The next statements are formulated regarding patients with cervical leak with pleural fluid collections. Indicate for each of the following statements if you agree with that statement. | | | | | | | | | | |
| Only non-invasive management/supportive care should be performed | 8 | 14 | 6 | 9 | 12 | 0 | 0 | 17 | 8 | 0 |
| Additional drainage interventions should be performed (e.g. opening cervical incision, EVT, drainage tube) | 92 | 86 | 92 | 93 | 77 | 100 | 100 | 83 | 97 | 80 |
| Additional leak closure interventions should be performed (e.g. stent, endoscopic tissue adhesives, surgical closure) | 59 | 57 | 65 | 52 | 82 | 67 | 86 | 83 | 48 | 40 |
| The next statements are formulated regarding patients with intrathoracic leak with only local manifestations and no fluid collections. Indicate for each of the following statements if you agree with that statement. | | | | | | | | | | |
| Only non-invasive management/supportive care should be performed | 53 | 57 | 44 | 62 | 41 | 67 | 29 | 67 | 53 | 100 |
| Additional drainage interventions should be performed (e.g. EVT, drainage tube) | 34 | 43 | 41 | 26 | 44 | 33 | 43 | 17 | 35 | 0 |
| Additional leak closure interventions should be performed (e.g. stent, endoscopic tissue adhesives, surgical closure) | 36 | 57 | 41 | 28 | 35 | 0 | 100 | 33 | 34 | 0 |
| **Statements** | **General** | **Low** | **Middle** | **High** | **Asia** | **Africa** | **North America** | **South America** | **Europe** | **Oceania** |
| The next statements are formulated regarding patients with intrathoracic leak with mediastinal fluid collections. Indicate for each of the following statements if you agree with that statement. | | | | | | | | | | |
| Only non-invasive management/supportive care should be performed | 7 | 14 | 4 | 9 | 12 | 0 | 0 | 17 | 5 | 20 |
| Additional drainage interventions should be performed (e.g. EVT, drainage tube) | 92 | 100 | 92 | 92 | 88 | 67 | 100 | 100 | 94 | 80 |
| Additional leak closure interventions should be performed (e.g. stent, endoscopic tissue adhesives, surgical closure) | 67 | 86 | 69 | 62 | 65 | 67 | 100 | 100 | 64 | 20 |
| The next statements are formulated regarding patients with intrathoracic leak with pleural fluid collections. Indicate for each of the following statements if you agree with that statement. | | | | | | | | | | |
| Only non-invasive management/supportive care should be performed | 9 | 14 | 10 | 6 | 6 | 0 | 14 | 33 | 6 | 20 |
| Additional drainage interventions should be performed (e.g. EVT, drainage tube) | 96 | 100 | 100 | 91 | 88 | 100 | 100 | 100 | 97 | 100 |
| Additional leak closure interventions should be performed (e.g. stent, endoscopic tissue adhesives, surgical closure) | 74 | 100 | 75 | 70 | 71 | 67 | 100 | 83 | 76 | 20 |
| Indicate for each of the following treatment options, if you agree that it should be the first choice treatment in patients with anastomotic leak and overall conduit ischemia/necrosis. | | | | | | | | | | |
| Primary esophageal diversion | 79 | 86 | 81 | 75 | 77 | 100 | 83 | 67 | 77 | 100 |
| Continuity-preserving treatment | 27 | 43 | 20 | 32 | 25 | 33 | 14 | 50 | 28 | 0 |
| Indicate for each of the following drainage tools if you agree that it is useful in patients with a cervical leak. | | | | | | | | | | |
| Bedside opening of the cervical incision | 81 | 100 | 71 | 89 | 88 | 75 | 86 | 67 | 84 | 20 |
| Nasogastric drainage tube | 62 | 71 | 52 | 73 | 59 | 50 | 43 | 0 | 70 | 80 |
| Surgical drainage tube | 49 | 57 | 52 | 43 | 53 | 75 | 29 | 50 | 48 | 40 |
| Radiological drainage tube | 38 | 29 | 37 | 41 | 47 | 25 | 29 | 50 | 35 | 60 |
| **Statements** | **General** | **Low** | **Middle** | **High** | **Asia** | **Africa** | **North America** | **South America** | **Europe** | **Oceania** |
| Endoscopic nasomediastinum drainage | 32 | 17 | 27 | 39 | 46 | 0 | 43 | 50 | 30 | 0 |
| Endoscopic vacuum therapy | 50 | 33 | 49 | 54 | 50 | 0 | 57 | 67 | 53 | 0 |
| Indicate for each of the following leak closure interventions if you agree that it is useful in patients with a cervical leak. | | | | | | | | | | |
| Stent placement (endoscopically or radiologically) | 20 | 14 | 18 | 23 | 35 | 25 | 14 | 0 | 19 | 0 |
| Endoscopic clipping | 16 | 29 | 15 | 14 | 13 | 0 | 17 | 67 | 13 | 0 |
| Endoscopic suturing | 6 | 0 | 5 | 8 | 7 | 0 | 0 | 0 | 7 | 0 |
| Endoscopic tissue adhesives | 12 | 14 | 14 | 10 | 14 | 33 | 0 | 50 | 9 | 0 |
| Endoscopic vacuum therapy combined with stent (e.g. VACStent) | 41 | 17 | 39 | 46 | 43 | 67 | 50 | 33 | 41 | 0 |
| Surgical closure | 7 | 14 | 8 | 5 | 6 | 0 | 0 | 0 | 10 | 0 |
| Indicate for each of the following drainage tools if you agree that it is useful in patients with an intrathoracic leak. | | | | | | | | | | |
| Nasogastric drainage tube | 74 | 86 | 69 | 78 | 71 | 67 | 57 | 33 | 80 | 80 |
| Surgical drainage tube | 72 | 100 | 69 | 72 | 65 | 100 | 71 | 100 | 70 | 80 |
| Radiological drainage tube | 71 | 50 | 65 | 79 | 71 | 0 | 71 | 60 | 72 | 100 |
| Endoscopic nasomediastinum drainage | 59 | 25 | 49 | 73 | 54 | 0 | 50 | 75 | 62 | 60 |
| Endoscopic vacuum therapy | 82 | 60 | 78 | 89 | 64 | 50 | 57 | 80 | 90 | 75 |
| Indicate for each of the following leak closure interventions if you agree that it is useful in patients with an intrathoracic leak. | | | | | | | | | | |
| Stent placement (endoscopically or radiologically) | 71 | 57 | 75 | 70 | 88 | 67 | 100 | 83 | 64 | 60 |
| Endoscopic clipping | 28 | 29 | 40 | 16 | 40 | 0 | 33 | 50 | 23 | 40 |
| Endoscopic suturing | 17 | 0 | 17 | 19 | 27 | 50 | 33 | 20 | 10 | 25 |
| Endoscopic tissue adhesives | 16 | 29 | 19 | 11 | 20 | 50 | 0 | 33 | 12 | 50 |
| Endoscopic vacuum therapy combined with stent (e.g. VACStent) | 77 | 83 | 84 | 70 | 55 | 67 | 71 | 83 | 82 | 67 |
| Surgical closure | 24 | 43 | 20 | 25 | 27 | 33 | 29 | 0 | 24 | 20 |
|  |  |  |  |  |  |  |  |  |  |  |
|  |  |  |  |  |  |  |  |  |  |  |
| **Statements** | **General** | **Low** | **Middle** | **High** | **Asia** | **Africa** | **North America** | **South America** | **Europe** | **Oceania** |
| Indicate for each of the following situations if you agree that it is reason for surgical reintervention. | | | | | | | | | | |
| When the patient with a leak shows signs of uncontrolled sepsis | 85 | 100 | 87 | 81 | 82 | 100 | 86 | 100 | 84 | 80 |
| Step-up after failure of initiated treatment of anastomotic leak | 77 | 86 | 73 | 80 | 77 | 100 | 83 | 67 | 78 | 60 |
| Early leakage (within the first 72 hours after esophagectomy) | 41 | 57 | 44 | 35 | 47 | 75 | 43 | 17 | 36 | 80 |
| Uncontained intrathoracic anastomotic leaks | 65 | 100 | 71 | 54 | 63 | 100 | 71 | 100 | 61 | 60 |
| *POD; postoperative day. CRP; C-reactive protein. CT; computed tomography. EVT; endoscopic vacuum therapy. Consensus defined as % respondents that agreed or strongly agreed with the statement.* | | | | | | | | | | |

| **Supplementary Table S6** Second-round Delphi survey consensus stratified by hospital volume and continent | | | | | | | | | | |  |  |  |  |  |  |  |  |  |  |
| --- | --- | --- | --- | --- | --- | --- | --- | --- | --- | --- | --- | --- | --- | --- | --- | --- | --- | --- | --- | --- |
|  |  | **Hospital volume** | | | **Continent** | | | | | |  |  |  |  |  |  |  |  |  |  |
| **Statements** | **General** | **Low** | **Middle** | **High** | **Asia** | **Africa** | **North America** | **South America** | **Europe** | **Oceania** |  |  |  |  |  |  |  |  |  |  |
|  |  |  |  |  |  |  |  |  |  |  |  |  |  |  |  |  |  |  |  |  |
| Number of respondents (n) | **136** | **22** | **64** | **50** | **15** | **5** | **10** | **8** | **83** | **15** |  |  |  |  |  |  |  |  |  |  |
| *Diagnosis* | | | | | | | | | | |  |  |  |  |  |  |  |  |  |  |
| Indicate for each clinical sign if you agree that that clinical sign, amongst other signs and biochemical results, is useful in the diagnostic process of anastomotic leak after esophagectomy. | | | | | | | | | | |  |  |  |  |  |  |  |  |  |  |
| Tachycardia | 84 | 96 | 83 | 80 | 93 | 100 | 100 | 100 | 76 | 93 |  |  |  |  |  |  |  |  |  |  |
| Atrial fibrillation (Altered) | 66 | 70 | 73 | 56 | 50 | 60 | 80 | 86 | 63 | 79 |  |  |  |  |  |  |  |  |  |  |
| Subcutaneous emphysema | 35 | 46 | 36 | 28 | 50 | 60 | 50 | 50 | 29 | 27 |  |  |  |  |  |  |  |  |  |  |
| Chest pain | 28 | 46 | 24 | 26 | 29 | 40 | 20 | 14 | 28 | 40 |  |  |  |  |  |  |  |  |  |  |
| Redness and swelling in the associated neck incision (cervical anastomosis) (altered) | 87 | 82 | 86 | 92 | 93 | 80 | 100 | 88 | 87 | 80 |  |  |  |  |  |  |  |  |  |  |
| Increased output of postoperative drain | 40 | 73 | 36 | 32 | 71 | 60 | 60 | 75 | 30 | 33 |  |  |  |  |  |  |  |  |  |  |
| Indicate for each biochemical test if you agree that that biochemical test, amongst other signs and biochemical tests, is useful in the diagnostic process of anastomotic leak after esophagectomy. | | | | | | | | | | |  |  |  |  |  |  |  |  |  |  |
| Drain amylase | 62 | 64 | 68 | 54 | 57 | 75 | 80 | 88 | 63 | 33 |  |  |  |  |  |  |  |  |  |  |
| Serum white blood count (WBC) | 83 | 91 | 88 | 74 | 86 | 100 | 90 | 88 | 80 | 87 |  |  |  |  |  |  |  |  |  |  |
| Serum procalcitonin (PCT) | 51 | 71 | 39 | 58 | 64 | 80 | 50 | 57 | 52 | 18 |  |  |  |  |  |  |  |  |  |  |
| Serum neutrophil count | 49 | 68 | 43 | 47 | 54 | 60 | 70 | 63 | 41 | 60 |  |  |  |  |  |  |  |  |  |  |
| Serum bilirubin | 4 | 10 | 2 | 4 | 7 | 20 | 0 | 13 | 3 | 0 |  |  |  |  |  |  |  |  |  |  |
| Serum lactate | 15 | 38 | 11 | 10 | 21 | 40 | 10 | 14 | 13 | 13 |  |  |  |  |  |  |  |  |  |  |
| Routine, daily measurement of CRP is useful as part of standard postoperative protocol after esophagectomy. (New) | 62 | 77 | 62 | 54 | 43 | 40 | 40 | 88 | 66 | 60 |  |  |  |  |  |  |  |  |  |  |
| Routine measurement of CRP on alternate days is useful as part of standard postoperative protocol after esophagectomy. (New) | 55 | 59 | 50 | 58 | 36 | 75 | 20 | 75 | 55 | 67 |  |  |  |  |  |  |  |  |  |  |
| **Statements** | **General** | **Low** | **Middle** | **High** | **Asia** | **Africa** | **North America** | **South America** | **Europe** | **Oceania** |  |  |  |  |  |  |  |  |  |  |
| If routine monitoring of CRP is performed, indicate for each postoperative day (POD) if you agree that routine monitoring should start on that POD. (New) | | | | | | | | | | |  |  |  |  |  |  |  |  |  |  |
| POD 1 | 47 | 52 | 42 | 50 | 55 | 60 | 60 | 50 | 45 | 40 |  |  |  |  |  |  |  |  |  |  |
| POD 2 | 44 | 38 | 46 | 43 | 50 | 25 | 40 | 25 | 45 | 50 |  |  |  |  |  |  |  |  |  |  |
| POD 3 | 74 | 62 | 69 | 87 | 84 | 60 | 40 | 88 | 76 | 64 |  |  |  |  |  |  |  |  |  |  |
| A CRP of 200mg/L, combined with other clinical signs and biochemical tests, is an useful cut-off value for considering additional diagnostics (e.g., CT scan, endoscopy). (New) | 64 | 81 | 52 | 72 | 69 | 100 | 60 | 88 | 61 | 60 |  |  |  |  |  |  |  |  |  |  |
| Indicate for each postoperative day (POD) if you agree that routine monitoring drain amylase on that day is useful in the diagnostic process of anastomotic leak. | | | | | | | | | | |  |  |  |  |  |  |  |  |  |  |
| POD 2 | 21 | 32 | 18 | 18 | 30 | 33 | 50 | 25 | 17 | 8 |  |  |  |  |  |  |  |  |  |  |
| POD 3 | 34 | 42 | 29 | 36 | 50 | 33 | 63 | 25 | 32 | 17 |  |  |  |  |  |  |  |  |  |  |
| POD 4 | 37 | 47 | 36 | 32 | 60 | 33 | 50 | 50 | 34 | 17 |  |  |  |  |  |  |  |  |  |  |
| POD 5 | 59 | 68 | 54 | 60 | 84 | 100 | 75 | 88 | 54 | 23 |  |  |  |  |  |  |  |  |  |  |
| POD 6 | 47 | 58 | 46 | 44 | 67 | 67 | 75 | 88 | 40 | 17 |  |  |  |  |  |  |  |  |  |  |
| POD 7 | 50 | 58 | 42 | 56 | 67 | 67 | 75 | 75 | 47 | 8 |  |  |  |  |  |  |  |  |  |  |
| The trend in drain amylase values is useful in the decision for additional diagnostics regarding anastomotic leak. | 34 | 62 | 19 | 40 | 46 | 50 | 56 | 50 | 28 | 23 |  |  |  |  |  |  |  |  |  |  |
| Measuring drain amylase levels is useful when suspecting anastomotic leak, based on suspicious drain fluid aspect. (New) | 52 | 57 | 52 | 50 | 62 | 50 | 60 | 75 | 54 | 14 |  |  |  |  |  |  |  |  |  |  |
| Measuring drain amylase levels is useful when suspecting anastomotic leak, based on clinical signs and/or biochemical results (e.g. elevated CRP), without suspicious drain fluid aspect. (New) | 51 | 57 | 53 | 44 | 69 | 75 | 50 | 75 | 48 | 29 |  |  |  |  |  |  |  |  |  |  |
| **Statements** | **General** | **Low** | **Middle** | **High** | **Asia** | **Africa** | **North America** | **South America** | **Europe** | **Oceania** |  |  |  |  |  |  |  |  |  |  |
| An amylase of 500mg/L, combined with other clinical signs and biochemical tests, is an useful cut-off value for considering additional diagnostics (e.g. CT, endoscopy). (New) | 56 | 55 | 55 | 58 | 62 | 100 | 50 | 75 | 57 | 25 |  |  |  |  |  |  |  |  |  |  |
| Screening for anastomotic leak using additional diagnostics (e.g. CT, endoscopy, radiography) should be performed in asymptomatic patients with normal biochemical results (New). | 25 | 36 | 25 | 20 | 21 | 80 | 50 | 25 | 19 | 27 |  |  |  |  |  |  |  |  |  |  |
| Screening for anastomotic leak in asymptomatic patients and normal biochemical results should be performed by …. | | | | | | | | | | |  |  |  |  |  |  |  |  |  |  |
| Routine contrast radiography | 29 | 41 | 32 | 21 | 33 | 60 | 44 | 63 | 22 | 29 |  |  |  |  |  |  |  |  |  |  |
| Routine computed tomography (CT) scan | 13 | 14 | 15 | 10 | 25 | 40 | 22 | 25 | 7 | 13 |  |  |  |  |  |  |  |  |  |  |
| Routine endoscopy | 2 | 0 | 3 | 2 | 17 | 0 | 0 | 0 | 1 | 0 |  |  |  |  |  |  |  |  |  |  |
| Methylene blue is useful when drains are still in place and you suspect cervical anastomotic leak based on clinical signs and/or biochemical results. (New) | 55 | 68 | 57 | 47 | 69 | 80 | 40 | 75 | 54 | 40 |  |  |  |  |  |  |  |  |  |  |
| If you suspect cervical anastomotic leak based on clinical signs and/or biochemical tests, indicate if you agree that bedside opening of the cervical incision is indicated in the following scenarios. (New) | | | | | | | | | | |  |  |  |  |  |  |  |  |  |  |
| Always | 30 | 27 | 32 | 27 | 43 | 60 | 40 | 25 | 28 | 13 |  |  |  |  |  |  |  |  |  |  |
| Local signs of infection (red/swollen) | 74 | 68 | 79 | 71 | 79 | 80 | 100 | 88 | 70 | 67 |  |  |  |  |  |  |  |  |  |  |
| No drains in place | 56 | 55 | 63 | 46 | 79 | 75 | 70 | 63 | 49 | 50 |  |  |  |  |  |  |  |  |  |  |
| The patient is septic | 76 | 73 | 79 | 73 | 93 | 80 | 100 | 75 | 73 | 60 |  |  |  |  |  |  |  |  |  |  |
| A patient with a cervical anastomosis is suspected of anastomotic leak based on systemic signs and/or biochemical tests without local sings of infection. The next diagnostic assessment to be performed is … (New) | | | | | | | | | | |  |  |  |  |  |  |  |  |  |  |
| Bedside opening of the cervical incision | 23 | 14 | 32 | 15 | 36 | 20 | 10 | 43 | 21 | 20 |  |  |  |  |  |  |  |  |  |  |
| Contrast radiography | 47 | 59 | 44 | 44 | 57 | 100 | 70 | 88 | 33 | 53 |  |  |  |  |  |  |  |  |  |  |
| Computed tomography (CT) scan | 80 | 86 | 79 | 79 | 71 | 60 | 80 | 100 | 84 | 64 |  |  |  |  |  |  |  |  |  |  |
| **Statements** | **General** | **Low** | **Middle** | **High** | **Asia** | **Africa** | **North America** | **South America** | **Europe** | **Oceania** |  |  |  |  |  |  |  |  |  |  |
| Endoscopy | 42 | 46 | 39 | 44 | 43 | 0 | 40 | 50 | 43 | 14 |  |  |  |  |  |  |  |  |  |  |
| CT scan and endoscopy | 54 | 43 | 56 | 57 | 36 | 20 | 60 | 57 | 65 | 21 |  |  |  |  |  |  |  |  |  |  |
| A patient with a cervical anastomosis is suspected of anastomotic leak based on systemic signs and/or biochemical tests and the cervical incision shows local sings of infection (red/swollen). The next diagnostic assessment to be performed is … (New) | | | | | | | | | | |  |  |  |  |  |  |  |  |  |  |
| Bedside opening of the cervical incision | 72 | 73 | 73 | 71 | 64 | 60 | 100 | 100 | 70 | 57 |  |  |  |  |  |  |  |  |  |  |
| Contrast radiography | 38 | 46 | 40 | 30 | 57 | 60 | 40 | 75 | 24 | 64 |  |  |  |  |  |  |  |  |  |  |
| Computed tomography (CT) scan | 77 | 86 | 73 | 79 | 64 | 60 | 80 | 100 | 80 | 64 |  |  |  |  |  |  |  |  |  |  |
| Endoscopy | 44 | 46 | 41 | 47 | 43 | 0 | 30 | 63 | 52 | 14 |  |  |  |  |  |  |  |  |  |  |
| CT scan and endoscopy | 54 | 43 | 55 | 57 | 50 | 40 | 40 | 57 | 63 | 21 |  |  |  |  |  |  |  |  |  |  |
| If you confirmed cervical anastomotic leak by opening the cervical incision and patient has mild symptoms (not septic), indicate for the following diagnostic assessments if you agree that it is useful to be performed next. (New) | | | | | | | | | | |  |  |  |  |  |  |  |  |  |  |
| Contrast radiography | 23 | 18 | 26 | 20 | 29 | 60 | 30 | 50 | 13 | 36 |  |  |  |  |  |  |  |  |  |  |
| Computed tomography (CT) scan | 69 | 68 | 73 | 63 | 71 | 40 | 60 | 88 | 75 | 36 |  |  |  |  |  |  |  |  |  |  |
| Endoscopy | 41 | 41 | 42 | 41 | 36 | 0 | 22 | 38 | 50 | 29 |  |  |  |  |  |  |  |  |  |  |
| CT scan and endoscopy | 45 | 48 | 44 | 46 | 50 | 20 | 33 | 43 | 51 | 29 |  |  |  |  |  |  |  |  |  |  |
| If you confirmed cervical anastomotic leak by opening the cervical incision and patient is septic, indicate for the following diagnostic assessment if you agree that it is useful to be performed next. (New) | | | | | | | | | | |  |  |  |  |  |  |  |  |  |  |
| Contrast radiography | 18 | 27 | 18 | 13 | 21 | 100 | 10 | 38 | 10 | 29 |  |  |  |  |  |  |  |  |  |  |
| Computed tomography (CT) scan | 89 | 86 | 92 | 85 | 93 | 100 | 100 | 88 | 89 | 71 |  |  |  |  |  |  |  |  |  |  |
| Endoscopy | 61 | 55 | 57 | 69 | 64 | 0 | 80 | 63 | 64 | 43 |  |  |  |  |  |  |  |  |  |  |
| CT scan and endoscopy | 69 | 57 | 65 | 80 | 71 | 0 | 70 | 71 | 74 | 57 |  |  |  |  |  |  |  |  |  |  |
| Methylene blue is useful when drains are still in place and you suspect intrathoracic anastomotic leak based on clinical signs and/or biochemical results. (New) | 47 | 68 | 48 | 35 | 62 | 50 | 40 | 75 | 46 | 27 |  |  |  |  |  |  |  |  |  |  |
| When a cervical or intrathoracic anastomotic leak is diagnosed on CT scan, it is useful to perform additional endoscopy. | 77 | 77 | 79 | 75 | 64 | 75 | 70 | 88 | 85 | 47 |  |  |  |  |  |  |  |  |  |  |
| **Statements** | **General** | **Low** | **Middle** | **High** | **Asia** | **Africa** | **North America** | **South America** | **Europe** | **Oceania** |  |  |  |  |  |  |  |  |  |  |
| The size of the defect should be measured during endoscopy in order to guide anastomotic leak treatment. | 85 | 91 | 81 | 88 | 86 | 75 | 70 | 100 | 86 | 87 |  |  |  |  |  |  |  |  |  |  |
| The condition of the gastric conduit (e.g. presence of conduit ischemia/necrosis) should be assessed during endoscopy in order to guide anastomotic leak treatment. (New) | 98 | 100 | 97 | 98 | 100 | 80 | 100 | 100 | 99 | 93 |  |  |  |  |  |  |  |  |  |  |
| The extent of contamination (e.g. mediastinal and/or pleural fluid collections) should be assessed on CT in order to guide anastomotic leak treatment. (New) | 99 | 100 | 100 | 96 | 100 | 100 | 90 | 100 | 99 | 100 |  |  |  |  |  |  |  |  |  |  |
| The individual risk of 90-day mortality as predicted by a ‘leak severity score’ is useful to guide anastomotic leak treatment. | 31 | 57 | 27 | 26 | 36 | 67 | 30 | 63 | 29 | 14 |  |  |  |  |  |  |  |  |  |  |
| *Treatment* | | | | | | | | | | |  |  |  |  |  |  |  |  |  |  |
| Indicate for each of the following treatment options if you agree that it should be part of non-invasive management/supportive care of all anastomotic leak patients. | | | | | | | | | | |  |  |  |  |  |  |  |  |  |  |
| Antifungal therapy (according to local protocol) | 74 | 68 | 73 | 78 | 57 | 60 | 70 | 38 | 83 | 67 |  |  |  |  |  |  |  |  |  |  |
| Nil-by-mouth | 84 | 96 | 81 | 82 | 100 | 80 | 70 | 88 | 83 | 80 |  |  |  |  |  |  |  |  |  |  |
| Clear water diet | 24 | 9 | 25 | 28 | 21 | 20 | 20 | 13 | 24 | 33 |  |  |  |  |  |  |  |  |  |  |
| Antacids | 32 | 41 | 21 | 43 | 57 | 20 | 50 | 0 | 28 | 43 |  |  |  |  |  |  |  |  |  |  |
| Proton-pump inhibitors (Altered) | 79 | 73 | 80 | 80 | 79 | 80 | 50 | 63 | 82 | 87 |  |  |  |  |  |  |  |  |  |  |
| The next statements are formulated regarding patients with a cervical leak with only local manifestations without fluid collections and mild symptoms. Indicate for each of the following statements if you agree with that statement. (Altered) | | | | | | | | | | |  |  |  |  |  |  |  |  |  |  |
| Only non-invasive management/supportive care should be performed | 48 | 50 | 51 | 43 | 57 | 20 | 20 | 75 | 46 | 60 |  |  |  |  |  |  |  |  |  |  |
| Additional drainage interventions should be performed (e.g. opening cervical incision, EVT, drainage tube) | 56 | 59 | 54 | 57 | 43 | 60 | 50 | 50 | 63 | 40 |  |  |  |  |  |  |  |  |  |  |
| **Statements** | **General** | **Low** | **Middle** | **High** | **Asia** | **Africa** | **North America** | **South America** | **Europe** | **Oceania** |  |  |  |  |  |  |  |  |  |  |
| Additional leak closure interventions should be performed (e.g. stent, endoscopic tissue adhesives, surgical closure) | 15 | 14 | 15 | 15 | 7 | 60 | 0 | 13 | 14 | 20 |  |  |  |  |  |  |  |  |  |  |
| The next statements are formulated regarding patients with a cervical leak without conduit ischemia/necrosis. Indicate for each scenario if, in addition to supportive care and drainage interventions, leak closure interventions (e.g. stent, EVT, surgical closure) should be performed when … (New) | | | | | | | | | | |  |  |  |  |  |  |  |  |  |  |
| The patient has mild symptoms and mediastinal fluid collections | 52 | 73 | 52 | 44 | 57 | 40 | 60 | 88 | 53 | 27 |  |  |  |  |  |  |  |  |  |  |
| The patient is septic and has mediastinal fluid collections | 70 | 77 | 69 | 67 | 71 | 60 | 90 | 100 | 67 | 60 |  |  |  |  |  |  |  |  |  |  |
| The patient has mild symptoms and pleural fluid collections | 56 | 77 | 55 | 48 | 50 | 60 | 60 | 100 | 58 | 27 |  |  |  |  |  |  |  |  |  |  |
| The patient is septic and has pleural fluid collections | 69 | 73 | 69 | 67 | 71 | 60 | 90 | 100 | 68 | 47 |  |  |  |  |  |  |  |  |  |  |
| Indicate for each of the following drainage tools if you agree that it is useful in patients with a cervical leak. | | | | | | | | | | |  |  |  |  |  |  |  |  |  |  |
| Nasogastric drainage tube | 44 | 36 | 40 | 54 | 50 | 20 | 20 | 13 | 50 | 47 |  |  |  |  |  |  |  |  |  |  |
| Surgical drainage tube | 35 | 59 | 30 | 31 | 57 | 20 | 10 | 13 | 35 | 53 |  |  |  |  |  |  |  |  |  |  |
| Radiological drainage tube | 22 | 41 | 13 | 25 | 43 | 40 | 0 | 25 | 19 | 27 |  |  |  |  |  |  |  |  |  |  |
| Endoscopic nasomediastinum drainage | 21 | 18 | 12 | 36 | 36 | 0 | 20 | 13 | 22 | 14 |  |  |  |  |  |  |  |  |  |  |
| Endoscopic vacuum therapy (EVT) | 55 | 55 | 53 | 56 | 57 | 40 | 60 | 38 | 57 | 50 |  |  |  |  |  |  |  |  |  |  |
| Surgical drainage (Altered) | 49 | 50 | 52 | 44 | 53 | 40 | 40 | 63 | 49 | 47 |  |  |  |  |  |  |  |  |  |  |
| Indicate for each of the following leak closure interventions if you agree that it is useful in patients with a cervical leak. | | | | | | | | | | |  |  |  |  |  |  |  |  |  |  |
| Stent placement (endoscopically or radiologically) | 14 | 29 | 15 | 6 | 36 | 0 | 10 | 29 | 11 | 7 |  |  |  |  |  |  |  |  |  |  |
| Endoscopic clipping | 7 | 19 | 5 | 4 | 21 | 0 | 0 | 0 | 8 | 0 |  |  |  |  |  |  |  |  |  |  |
| Endoscopic suturing | 4 | 14 | 2 | 2 | 14 | 0 | 0 | 0 | 4 | 0 |  |  |  |  |  |  |  |  |  |  |
| Endoscopic tissue adhesives | 2 | 5 | 0 | 2 | 7 | 0 | 0 | 0 | 1 | 0 |  |  |  |  |  |  |  |  |  |  |
| Endoscopic vacuum therapy (EVT) (Altered) | 50 | 57 | 53 | 43 | 62 | 0 | 50 | 63 | 49 | 50 |  |  |  |  |  |  |  |  |  |  |
| **Statements** | **General** | **Low** | **Middle** | **High** | **Asia** | **Africa** | **North America** | **South America** | **Europe** | **Oceania** |  |  |  |  |  |  |  |  |  |  |
| Endoscopic vacuum therapy combined with stent (e.g. VACStent) | 21 | 42 | 16 | 17 | 21 | 25 | 11 | 14 | 23 | 15 |  |  |  |  |  |  |  |  |  |  |
| Surgical closure | 6 | 14 | 3 | 6 | 21 | 0 | 0 | 0 | 5 | 7 |  |  |  |  |  |  |  |  |  |  |
| The next statements are formulated regarding patients with an intrathoracic leak with only local manifestations without fluid collections and mild symptoms. Indicate for each of the following statements if you agree with that statement. (Altered) | | | | | | | | | | |  |  |  |  |  |  |  |  |  |  |
| Only non-invasive management/supportive care should be performed | 44 | 50 | 43 | 44 | 64 | 25 | 20 | 38 | 43 | 60 |  |  |  |  |  |  |  |  |  |  |
| Additional drainage interventions should be performed (e.g. EVT, drainage tube) | 48 | 50 | 54 | 40 | 36 | 25 | 20 | 50 | 58 | 27 |  |  |  |  |  |  |  |  |  |  |
| Additional leak closure should be performed (e.g. stent, EVT, surgical closure) | 40 | 38 | 44 | 34 | 36 | 50 | 30 | 43 | 43 | 27 |  |  |  |  |  |  |  |  |  |  |
| The following statements are formulated regarding patients with an intrathoracic leak without conduit ischemia/necrosis. Indicate for each scenario if, in addition to supportive care and drainage interventions, leak closure interventions (e.g. stent, EVT, surgical closure) should be performed when … (New) | | | | | | | | | | |  |  |  |  |  |  |  |  |  |  |
| The patient has mild symptoms and mediastinal fluid collections | 67 | 64 | 76 | 57 | 71 | 50 | 50 | 88 | 73 | 33 |  |  |  |  |  |  |  |  |  |  |
| The patient is septic and mediastinal fluid collections | 83 | 82 | 87 | 78 | 71 | 75 | 100 | 88 | 84 | 73 |  |  |  |  |  |  |  |  |  |  |
| The patient has mild symptoms and pleural fluid collections | 77 | 82 | 86 | 65 | 86 | 100 | 90 | 88 | 74 | 67 |  |  |  |  |  |  |  |  |  |  |
| The patient is septic and has pleural fluid collections | 83 | 77 | 87 | 80 | 79 | 75 | 100 | 88 | 83 | 73 |  |  |  |  |  |  |  |  |  |  |
| Indicate for each of the following drainage tools if you agree that it is useful in patients with an intrathoracic leak. | | | | | | | | | | |  |  |  |  |  |  |  |  |  |  |
| Nasogastric drainage tube | 76 | 64 | 76 | 80 | 79 | 50 | 70 | 50 | 79 | 80 |  |  |  |  |  |  |  |  |  |  |
| Surgical drainage tube | 79 | 100 | 75 | 76 | 71 | 100 | 80 | 88 | 79 | 80 |  |  |  |  |  |  |  |  |  |  |
| Radiological drainage tube | 81 | 91 | 84 | 72 | 64 | 75 | 90 | 86 | 80 | 93 |  |  |  |  |  |  |  |  |  |  |
| Endoscopic nasomediastinum drainage | 44 | 43 | 39 | 51 | 29 | 0 | 22 | 43 | 48 | 60 |  |  |  |  |  |  |  |  |  |  |
| Surgical drainage (Altered) | 49 | 68 | 41 | 50 | 57 | 25 | 50 | 50 | 49 | 47 |  |  |  |  |  |  |  |  |  |  |
| **Statements** | **General** | **Low** | **Middle** | **High** | **Asia** | **Africa** | **North America** | **South America** | **Europe** | **Oceania** |  |  |  |  |  |  |  |  |  |  |
| Indicate for each of the following leak closure interventions if you agree that it is useful in patients with an intrathoracic leak. | | | | | | | | | | |  |  |  |  |  |  |  |  |  |  |
| Stent placement (endoscopically or radiologically) | 65 | 91 | 67 | 52 | 71 | 75 | 80 | 71 | 63 | 53 |  |  |  |  |  |  |  |  |  |  |
| Endoscopic clipping | 17 | 45 | 16 | 8 | 21 | 33 | 10 | 29 | 14 | 27 |  |  |  |  |  |  |  |  |  |  |
| Endoscopic suturing | 7 | 26 | 6 | 0 | 7 | 0 | 0 | 0 | 7 | 13 |  |  |  |  |  |  |  |  |  |  |
| Endoscopic tissue adhesives | 6 | 16 | 5 | 2 | 7 | 0 | 0 | 0 | 5 | 14 |  |  |  |  |  |  |  |  |  |  |
| Endoscopic vacuum therapy (EVT) (Altered) | 82 | 81 | 83 | 82 | 65 | 0 | 80 | 100 | 88 | 73 |  |  |  |  |  |  |  |  |  |  |
| Endoscopic vacuum therapy combined with stent (e.g. VACStent) | 73 | 95 | 70 | 67 | 57 | 100 | 80 | 71 | 77 | 57 |  |  |  |  |  |  |  |  |  |  |
| Surgical closure | 20 | 29 | 19 | 18 | 36 | 0 | 20 | 0 | 17 | 40 |  |  |  |  |  |  |  |  |  |  |
| Indicate for each of the following treatment options, if you agree that it should be the first choice treatment for patients with anastomotic leak and mild symptoms, and limited ischemia/necrosis of the top. (New) | | | | | | | | | | |  |  |  |  |  |  |  |  |  |  |
| Primary esophageal diversion | 9 | 24 | 7 | 6 | 21 | 0 | 0 | 38 | 6 | 7 |  |  |  |  |  |  |  |  |  |  |
| Continuity-preserving treatment | 84 | 77 | 84 | 88 | 64 | 75 | 90 | 75 | 89 | 80 |  |  |  |  |  |  |  |  |  |  |
| Indicate for each of the following treatment options, if you agree that it should be the first choice treatment for septic patients with anastomotic leak, and limited ischemia/necrosis of the top. (New) | | | | | | | | | | |  |  |  |  |  |  |  |  |  |  |
| Primary esophageal diversion | 27 | 48 | 23 | 24 | 64 | 60 | 20 | 38 | 23 | 7 |  |  |  |  |  |  |  |  |  |  |
| Continuity-preserving treatment | 61 | 46 | 65 | 62 | 36 | 50 | 60 | 63 | 64 | 67 |  |  |  |  |  |  |  |  |  |  |
| Indicate for each of the following treatment options, if you agree that it should be the first choice treatment for patients with anastomotic leak and mild symptoms, and overall ischemia/necrosis of the top. (New) | | | | | | | | | | |  |  |  |  |  |  |  |  |  |  |
| Primary esophageal diversion | 75 | 86 | 73 | 74 | 100 | 75 | 70 | 100 | 72 | 57 |  |  |  |  |  |  |  |  |  |  |
| Continuity-preserving treatment | 19 | 14 | 21 | 20 | 21 | 25 | 10 | 13 | 19 | 27 |  |  |  |  |  |  |  |  |  |  |
| Indicate for each of the following treatment options, if you agree that it should be the first choice treatment for septic patients with anastomotic leak, and overall ischemia/necrosis of the top. (New) | | | | | | | | | | |  |  |  |  |  |  |  |  |  |  |
| Primary esophageal diversion | 89 | 91 | 86 | 92 | 93 | 75 | 90 | 100 | 89 | 79 |  |  |  |  |  |  |  |  |  |  |
| Continuity-preserving treatment | 5 | 0 | 10 | 2 | 7 | 0 | 10 | 0 | 4 | 13 |  |  |  |  |  |  |  |  |  |  |
| **Statements** | **General** | **Low** | **Middle** | **High** | **Asia** | **Africa** | **North America** | **South America** | **Europe** | **Oceania** |  |  |  |  |  |  |  |  |  |  |
| Indicate for each of the following situations if you agree that it is reason for surgical washout and better drainage. (New) | | | | | | | | | | |  |  |  |  |  |  |  |  |  |  |
| When the patient with a leak shows signs of uncontrolled sepsis | 84 | 96 | 86 | 84 | 86 | 80 | 100 | 100 | 81 | 87 |  |  |  |  |  |  |  |  |  |  |
| Step-up after failure of initiated treatment of anastomotic leak | 82 | 86 | 76 | 88 | 71 | 80 | 100 | 88 | 83 | 73 |  |  |  |  |  |  |  |  |  |  |
| Early leakage (within the first 72 hours after esophagectomy) | 41 | 55 | 43 | 32 | 43 | 60 | 50 | 50 | 34 | 60 |  |  |  |  |  |  |  |  |  |  |
| Uncontained intrathoracic anastomotic leaks | 74 | 91 | 65 | 78 | 79 | 75 | 70 | 100 | 69 | 87 |  |  |  |  |  |  |  |  |  |  |
| Indicate for each of the following situations if you agree that it is reason for surgical repair of the anastomotic defect. (New) | | | | | | | | | | |  |  |  |  |  |  |  |  |  |  |
| When the patient with a leak shows signs of uncontrolled sepsis | 29 | 23 | 29 | 32 | 36 | 0 | 20 | 13 | 33 | 27 |  |  |  |  |  |  |  |  |  |  |
| Step-up after failure of initiated treatment of anastomotic leak | 35 | 41 | 27 | 42 | 50 | 25 | 10 | 13 | 38 | 40 |  |  |  |  |  |  |  |  |  |  |
| Early leakage (within the first 72 hours after esophagectomy) | 50 | 64 | 47 | 48 | 43 | 100 | 50 | 38 | 46 | 73 |  |  |  |  |  |  |  |  |  |  |
| Uncontained intrathoracic anastomotic leaks | 34 | 46 | 29 | 36 | 36 | 0 | 40 | 25 | 37 | 27 |  |  |  |  |  |  |  |  |  |  |
| Indicate for each of the following situations if you agree that it is reason for esophageal diversion. (New) | | | | | | | | | | |  |  |  |  |  |  |  |  |  |  |
| When the patient with a leak shows signs of uncontrolled sepsis | 57 | 82 | 55 | 50 | 86 | 100 | 60 | 75 | 48 | 60 |  |  |  |  |  |  |  |  |  |  |
| Step-up after failure of initiated treatment of anastomotic leak | 52 | 73 | 47 | 50 | 57 | 100 | 50 | 75 | 44 | 67 |  |  |  |  |  |  |  |  |  |  |
| Early leakage (within the first 72 hours after esophagectomy) | 10 | 18 | 8 | 8 | 21 | 50 | 0 | 38 | 2 | 20 |  |  |  |  |  |  |  |  |  |  |
| Uncontained intrathoracic anastomotic leaks | 38 | 59 | 37 | 30 | 71 | 75 | 30 | 88 | 23 | 60 |  |  |  |  |  |  |  |  |  |  |
| *CRP; C-reactive protein. POD; postoperative day. CT; computed tomography. Consensus defined as % respondents that agreed or strongly agreed with the statement.* | | | | | | | | | | |  |  |  |  |  |  |  |  |  |  |

# **Stage 3: Guideline development meetings – Overview of the recommendations regarding the diagnosis and treatment of anastomotic leak after esophagectomy, including the level of evidence, relevant articles, the strength of recommendation, corresponding results from the two-round Delphi survey and additional remarks and considerations of the expert panel.**

Levels of evidence adapted from the European Society of Cardiology (ESV) evidence grading system:

- Level of evidence A: Data derived from multiple randomised clinical trials or meta analyses
- Level of evidence B: Data derived from a single randomised clinical trial or large non-randomised studies
- Level of evidence C: Consensus of opinion of the experts and/or small studies, retrospective studies, registries.

Strength of recommendations adapted from the European Society of Cardiology (ESV) evidence grading system:

- Strength I: Evidence and or general agreement that a given treatment or procedure is beneficial, useful, effective.
- Conflicting evidence and or divergence of opinion about the usefulness/efficacy about the given treatment or procedure.
  - Strength IIa: Weight of evidence/opinion is in favour of usefulness/efficacy.
  - Strength IIb: Usefulness/efficacy is less well established by evidence/opinion.
  - Strength III: Evidence or general agreement that the given treatment or procedure is not useful/effective and in some cases may be harmful

**Diagnostic recommendations**

| **Recommendation 1A** | | |
| --- | --- | --- |
| Changed aspect of postoperative drain fluids is suggestive for an anastomotic leak. | | |
| Level of evidence | Strength of recommendation | References |
| C | I | Fabbi et al (2021), Grimminger et al (2018), Jones et al (2015), Low et al (2011), Min et al (2019), Moon et al (2019), Page et al (2005), Tsujimoto et al (2012), Bhasin et al (2000). |
| Delphi statements *(% consensus)*:   - Changed aspect of postoperative drain fluids is a useful clinical sign in the diagnostic process of anastomotic leak after esophagectomy *(92% consensus).* | | |
| Additional remarks/considerations from the expert panel:   - The use of changed aspect of postoperative fluids is a universally accessible clinical sign and comes at no cost. - The changed aspect of the fluid is a specific indicator of AL, especially if it resembles enteric content. Although an alternative diagnosis to consider is a chyle leak, identified by the milky aspect of the fluid. | | |

| **Recommendation 1B** | | |
| --- | --- | --- |
| Any of the following clinical signs can be taken into account for the diagnosis of an anastomotic leak: fever, respiratory distress, tachycardia. | | |
| Level of evidence | Strength of recommendation | References |
| C | IIa | Fabbi et al (2021), Grimminger et al (2018), Honing et al (2009), Jones et al (2015), Low et al (2011), Min et al (2019), Moon et al (2019), Tsujimoto et al (2012), Yin et al (2012). |
| Delphi statements *(% consensus)*:   - Fever (temperature > 38,5 degrees Celsius) is a useful clinical sign in the diagnostic process of anastomotic leak after esophagectomy *(85% consensus).* - Respiratory distress (e.g. tachypnea, oxygen delivery increase) is a useful clinical sign in the diagnostic process of anastomotic leak after esophagectomy *(81% consensus).* - Tachycardia is a useful clinical sign in the diagnostic process of anastomotic leak after esophagectomy *(84% consensus)*. | | |
| Additional remarks/considerations from the expert panel:   - Despite the weak quality of evidence, it is recommended to use some form of clinical judgment. - However, the expert panel believes that these clinical signs are not specific for AL. Pulmonary complications or other infectious issues can present with a similar clinical picture. Nevertheless, these signs should raise suspicion in the treating physician for a potential AL, and further investigation to exclude AL is warranted. | | |

| **Recommendation 2** | | |
| --- | --- | --- |
| Redness and swelling of the neck incision in patients with a cervical anastomosis are suggestive for an anastomotic leak. | | |
| Level of evidence | Strength of recommendation | References |
| C | I | Fabbi et al (2021), Grimminger et al (2018), Jones et al (2015), Low et al (2011), Min et al (2019), Moon et al (2019). |
| Delphi statements *(% consensus)*:   - Redness and swelling in the associated neck incision (cervical anastomosis) is a useful clinical sign in the diagnostic process of anastomotic leak after esophagectomy *(87% consensus)*. | | |
| Additional remarks/considerations from the expert panel:   - If the cervical incision appears visibly infected (red and/or swollen), it raises a high suspicion of issues with the underlying anastomosis, warranting additional diagnostic investigations for a possible AL. | | |

| **Recommendation 3** | | |
| --- | --- | --- |
| Serum C-Reactive Protein (CRP) can be useful in the diagnostic process for anastomotic leak, and monitoring the trend from postoperative day 3 to 7 after esophagectomy can be considered. | | |
| Level of evidence | Strength of recommendation | References |
| C | IIa | Aiolfi et al (2018), Andreatta et al (2022), Asti et al (2018), Azer et al (2022), Barbaro et al (2021), de Mooij et al (2019), Giulini et al (2019), Lemmens et al (2023), McAnena et al (2020), Noble et al (2012), Park et al (2017), Prochazka et al (2018), Rat et al (2022), Shao et al (2019), Stuart et al (2022), Zhang et al (2021), Yu et al (2019). |
| Delphi statements *(% consensus)*:   - CRP is a useful biochemical test in the diagnostic process of anastomotic leak after esophagectomy *(90% consensus)*. - Postoperative day 5 is a useful day to monitor CRP in the diagnostic process of anastomotic leak *(86% consensus)*. - The trend in CRP values is useful in the decision for additional diagnostics regarding anastomotic leak *(89% consensus)*. - If routine monitoring of CRP is performed, monitoring should start on POD 3 *(74% consensus)*. - Postoperative day 6 is a useful day to monitor CRP in the diagnostic process of anastomotic leak *(76% consensus)*. - Postoperative day 7 is a useful day to monitor CRP in the diagnostic process of anastomotic leak *(72% consensus)*. | | |
| Additional remarks/considerations from the expert panel:   - Serial measurements of serum CRP to monitor the trend can be useful in the diagnostic process of AL. Unfortunately, CRP is not specific to AL. It can be elevated as a normal response of the body to the trauma of surgery or may rise due to other infectious complications. However, if an increase in CRP is observed, or if it continues to be high after postoperative day 3, especially in combination with other clinical signs, it can be used as a diagnostic criterion for further investigation. - Additionally, CRP is a good negative predictor, and can therefore be helpful for the exclusion of AL. - 200 mg/L was most frequently chosen as the cutoff value during the first Delphi survey round and may be considered, but it did not reach consensus during the second round. Recommending a cutoff value for CRP is challenging as it depends on the postoperative day on which it is measured and quality of the literature is weak. Of greater significance is the evaluation of the CRP trend - If CRP is not available, an alternative may be WBC. However, the literature indicates a lower diagnostic accuracy for WBC compared to CRP, and therefore, it is not included in the recommendations of this guideline. | | |

| **Recommendation 4** | | |
| --- | --- | --- |
| If drains are placed during esophagectomy, drain amylase levels might be useful in the diagnostic process for anastomotic leak. | | |
| Level of evidence | Strength of recommendation | References |
| C | IIb | Andreatta et al (2022), Barbaro et al (2021), Baker et al (2016), Berkelmans et al (2015) de Mooij et al (2019), Matsumoto et al (2021), Perry et al (2015), Stuart et al (2022), Yu et al (2019). |
| Delphi statements with (near) consensus:   - Drain amylase is a useful biochemical test in the diagnostic process of anastomotic leak after esophagectomy *(74% consensus)*. | | |
| Additional remarks/considerations from the expert panel:   - While literature suggests the potential benefits of using drain amylase levels, the quality of evidence is low. The panel suggests considering drain amylase values in the diagnostic process if a drain is placed during surgery. However, a low level of drain amylase does not exclude AL. If the value is high and trending upwards, particularly when combined with other clinical signs, it may be useful as a diagnostic indicator for further investigation. - It's important to note that amylase levels can also be elevated due to leakage of gastric contents during the creation of the anastomosis. | | |

| **Recommendation 5** | | |
| --- | --- | --- |
| For patients with a suspected intrathoracic anastomotic leak, CT with oral contrast should be performed as the first diagnostic modality. | | |
| Level of evidence | Strength of recommendation | References |
| C | I | Barbaro et al (2021), El-Sourani et al (2022), Lantos et al (2013), Lemmens et al (2023), Plat et al (2020), Song et al (2020), Strauss et al (2010), Veziant et al (2022) |
| Delphi statements *(% consensus)*:   - Performing a computed tomography (CT) is the first choice diagnostic assessment if you suspect intrathoracic anastomotic leak based on clinical sign(s) and/or biochemical test(s) *(84% consensus)*. | | |
| Additional remarks/considerations from the expert panel:   - CT with oral contrast is recommended as the primary diagnostic tool for AL because it provides immediate information on the extent of the leakage, including the degree of mediastinal and/or pleural contamination. - Additionally, a CT scan can diagnose potential other complications. - CT-scoring systems have been proposed to increase diagnostic accuracy of the CT-scan and can be considered in clinical practice. | | |

| **Recommendation 6** | | |
| --- | --- | --- |
| For patients with suspected cervical leak without local signs of infection, a CT with oral contrast can be performed as the first diagnostic modality. | | |
| Level of evidence | Strength of recommendation | References |
| C | IIa | Barbaro et al (2021), Goense et al (2017), Lantos et al (2013), Lemmens et al (2023), Shoji et al (2018), Song et al (2020) |
| Delphi statements *(% consensus)*:   - When a patient with a cervical anastomosis is suspected of a leak based on systemic sign(s) and/or biochemical test(s) without local signs of infection, the next diagnostic assessment to be performed is a CT *(80% consensus)*. | | |
| Additional remarks/considerations from the expert panel:   - CT with oral contrast is also recommended as the first-choice diagnostic modality for AL in patients with a cervical anastomosis, for the same reasons as with intrathoracic anastomosis. However, due to a potential risk of aspiration with a high anastomosis, some of the experts use a CT without oral contrast or endoscopy as an alternative. | | |

| **Recommendation 7** | | |
| --- | --- | --- |
| For patients with suspected cervical leak with local signs of infection, CT with oral contrast or bedside opening of the incision can be used as a diagnostic of first choice. | | |
| Level of evidence | Strength of recommendation | References |
| C | IIa | Barbaro et al (2021), Goense et al (2017), Lantos et al (2013), Lemmens et al (2023), Shoji et al (2017), Song et al (2020) |
| Delphi statements *(% consensus)*:   - When a patient with a cervical anastomosis is suspected of a leak and the cervical incision shows local signs of infection, the next diagnostic assessment to be performed is a CT *(77% consensus)*. - When a patient with a cervical anastomosis is suspected of a leak and the cervical incision shows local signs of infection, the next diagnostic assessment to be performed is bedside opening the cervical incision *(72% consensus)*. - If a cervical leak is suspected, bedside opening of the cervical incision is indicated when the patient shows local signs of infection (red/swollen) *(74% consensus)*. - If a cervical leak is suspected, bedside opening of the cervical incision is indicated when the patient is septic *(76% consensus)*. | | |
| Additional remarks/considerations from the expert panel:   - The cervical incision should be opened when the patient is septic and/or shows local signs of infection, at least for effective drainage. However, some experts prefer to conduct a CT scan first and then determine, based on the CT scan findings, whether it is necessary to open the incision in the operating room for a more thorough exploration. - Alternatively, some experts advocate opening the cervical incision first and then performing a CT scan. Both options are considered valid diagnostic approaches, which also reflects the international opinion based on the Delphi survey results. | | |

| **Recommendation 8** | | |
| --- | --- | --- |
| For septic patients with an anastomotic leak confirmed by bedside opening of the cervical incision, an additional CT scan should be performed*.* | | |
| Level of evidence | Strength of recommendation | References |
| C | I | Barbaro et al (2021), Grimminger et al (2018). |
| Delphi statements *(% consensus)*:   - When a cervical leak is confirmed by opening the cervical incision and the patient is septic, it is useful to perform a CT *(89% consensus)*. - When a cervical leak is confirmed by opening the cervical incision and the patient has mild symptoms, it is useful to perform a CT *(69% consensus)*. | | |
| Additional remarks/considerations from the expert panel:   - Even if an anastomotic leak has already been confirmed by bedside opening of the cervical incision, it is still recommended to proceed with a CT scan to identify complications of the leak, such as mediastinal contamination. - However, if the patient is clinically well and there are no concerns of mediastinal collections, the endoscopy may be omitted. | | |

| **Recommendation 9** | | |
| --- | --- | --- |
| If anastomotic leak is confirmed by endoscopy, an additional CT scan with oral contrast should be performed to assess the extent of contamination. | | |
| Level of evidence | Strength of recommendation | References |
| C | I | Goense et al (2017), Plat et al (2020), Ubels et al (2022) |
| Delphi statements *(% consensus)*:   - When an anastomotic leak is diagnosed on endoscopy, it is useful to perform an additional CT scan *(90% consensus)*. - The extent of contamination (e.g. mediastinal and/or pleural fluid collections) should be assessed on CT in order to guide anastomotic leak treatment *(99% consensus)*. | | |
| Additional remarks/considerations from the expert panel:   - As stated by recommendations 5-8, a CT-scan is recommended as first choice diagnostic modality to assess the extent of contamination before diagnosis and/or treatment by endoscopy. This information can be necessary to decide on which potential endoscopic treatment to perform. If, for any reason, an endoscopy was performed first, we still recommend to perform a CT-scan afterwards. | | |

| **Recommendation 10** | | |
| --- | --- | --- |
| If anastomotic leak is confirmed by CT scan, an additional endoscopy can be performed to assess the size of the defect and condition of the gastric conduit. | | |
| Level of evidence | Strength of recommendation | References |
| C | IIa | El-Sourani et al (2022), Griffin et al (2001), Lin et al (2017), Moon et al (2019), Page et al (2013), Song et al (2020), Ubels et al (2022), Veziant et al (2022) |
| Delphi statements *(% consensus)*:   - When a cervical or intrathoracic anastomotic leak is diagnosed on CT, it is useful to perform additional endoscopy *(77% consensus)*. - The size of the defect should be measured during endoscopy in order to guide anastomotic leak treatment *(85% consensus)*. - The condition of the gastric conduit (e.g. presence of conduit ischemia/necrosis) should be assessed during endoscopy in order to guide anastomotic leak treatment *(98% consensus)*. | | |
| Additional remarks/considerations from the expert panel:   - An additional endoscopy is recommended to assess the size of the defect and the condition of the gastric conduit. It also provides the opportunity to initiate treatment during the procedure. - Omitting endoscopy in a patient with a cervical anastomosis and mild symptoms can be considered. However, there should be a low threshold to perform an endoscopy if there are any signs of issues with the vitality of the gastric conduit. | | |

| **Recommendation 11** | | |
| --- | --- | --- |
| For patients with a persistent suspicion of anastomotic leak, despite a negative or inconclusive CT scan, an additional endoscopy should be performed. | | |
| Level of evidence | Strength of recommendation | References |
| C | I | Moon et al (2019), El-Sourani et al (2022), Lemmens et al (2023), Page et al (2013), Song et al (2020), Strauss et al (2010), Veziant et al (2022), |
| Delphi statements *(% consensus)*:   - None | | |
| Additional remarks/considerations from the expert panel:   - The expert panel reached consensus that if there is persistent clinical suspicion of AL, an endoscopy should be performed even after a negative or inconclusive CT scan (the day before). This aligns with the literature indicating higher sensitivity for endoscopy. | | |

| **Recommendation 12** | | |
| --- | --- | --- |
| Routine screening for anastomotic leak in asymptomatic postoperative patients is not considered useful. | | |
| Level of evidence | Strength of recommendation | References |
| C | III | Barbaro et al (2021), Boone et al (2008), Cools-Lartigue et al (2014), Jones et al (2015), Jones et al (2015), Kang et al (2022), Lin et al (2017), Moon et al (2019), Murray et al (2018), Nederlof et al (2017), Page et al (2013), Shoji et al (2018), Song et al (2020), Strauss et al (2010), Upponi et al (2008), Yonis et al (2019). |
| Delphi statements *(% consensus)*:   - Screening should not be performed in asymptomatic patients with normal biochemical results *(58% consensus).* - Screening for anastomotic leak in asymptomatic patients and normal biochemical results should not be performed by endoscopy *(84% consensus)*. - Screening for anastomotic leak in asymptomatic patients and normal biochemical results should not be performed by CT scan *(71% consensus)*. - Screening for anastomotic leak in asymptomatic patients and normal biochemical results should not be performed by radiography *(59% consensus)*. | | |
| Additional remarks/considerations from the expert panel:   - A meta-analysis of small cohort studies examining the routine use of radiological contrast swallow concluded that it lacks adequate sensitivity and positive predictive value to be considered an effective screening tool. Additionally, there is a risk of aspiration associated with using contrast swallow. - The panel concludes that, given the current lack of evidence and the associated risk of complications from both radiological contrast swallow and endoscopy, routine screening is not considered useful. | | |

**Treatment recommendations**

| **Recommendation 13A** | | |
| --- | --- | --- |
| The initial supportive care treatment for anastomotic leak, should include broad-spectrum antibiotics, feeding support, and nil-by-mouth. | | |
| Level of evidence | Strength of recommendation | References |
| C | I | Al-issa et al (2015), Bhasin et al (2000), Birla et al (2019), Chen et al (2020), Chevallay et al (2020), Chon et al (2022), Fabbi et al (2021), Griffin et al (2001), Griffiths et al (2022), Grimminger et al (2018), Hünerbein et al (2004), Jones et al (2015), Low et al (2011), Ma et al (2018), Mardin et al (2012), Page et al (2005), Plum et al (2018), Schaheen et al (2014), Shuto et al (2017), Turkyilmaz et al 2009, Ubels et al (2023), Verstegen et al (2019), Xu et al (2016), Yin et al (2012), Yin et al (2021), Zhang et al (2017), Zhong et al (2021). |
| Delphi statements *(% consensus)*:   - Broad spectrum antibiotics (according to local protocol) should be part of non-invasive management/supportive care of all anastomotic leak patients *(99% consensus).* - Feeding support (e.g. enteral feeding using jejunal tube placement, jejunostomy or parenteral feeding) should be part of non-invasive management/supportive care of all anastomotic leak patients *(98% consensus)*. - Nil-by-mouth should be part of best supportive care in all leak patients *(84% consensus)*. | | |
| Additional remarks/considerations from the expert panel:   - The recommendation for nil-by-mouth depends on the timing in the treatment of AL. Initially, all leak patients should be treated with nil-by-mouth. However, if the leak is controlled, e.g., by stent, oral intake can be gradually increased. - Additionally, consider allowing patients to drink water for drainage after opening the cervical incision. | | |

| **Recommendation 13B** | | |
| --- | --- | --- |
| Proton-pump inhibitors (PPI) can be used or continued as supportive care treatment for anastomotic leak. | | |
| Level of evidence | Strength of recommendation | References |
| C | IIa | Fabbi et al (2021), Grimminger et al (2018), Shuto et al (2017), Xu et al (2016), Yin et al (2021), Zhong et al (2021). |
| Delphi statements *(% consensus)*:   - PPI should be part of best supportive care in all leak patients *(79% consensus).* | | |
| Additional remarks/considerations from the expert panel:   - Many experts use PPIs as standard postoperative care after esophageal surgery, not only when a leak occurs. Unfortunately, there is no scientific evidence supporting this practice, other than the theory that it reduces fluid leakage into the leak cavity and makes the fluid less toxic to tissues by increasing the pH. | | |

| **Recommendation 13C** | | |
| --- | --- | --- |
| Antifungal therapy may be considered as supportive care treatment for anastomotic leak. | | |
| Level of evidence | Strength of recommendation | References |
| C | IIb | Griffin et al (2001), Grimminger et al (2018), Low et al (2011), Plum et al (2018). |
| Delphi statements *(% consensus)*:   - Antifungal therapy should be part of best supportive care in all leak patients *(74% consensus).* | | |
| Additional remarks/considerations from the expert panel:   - There are variations in the use of antifungal therapy among experts. Some may choose a prophylactic approach, but this can contribute to increased resistance and the potential need for a more complex antifungal treatment in the future. To consider is the use of antifungal therapy after a specific time period or guided by cultures, in consultation with the medical microbiologist or infectious disease specialist. - Other considerations for the panel to refrain from initiating or continuing antifungal therapy include when the leak is well-controlled, e.g., through drainage, or when dealing with a small, contained leak. | | |

| **Recommendation 14** | | |
| --- | --- | --- |
| Nasogastric tube drainage, for drainage and decompression of the gastric conduit, should be considered during the initial treatment of all leak patients. | | |
| Level of evidence | Strength of recommendation | References |
| C | I | Fabbi et al (2021), Famiglietti et al (2020), Griffin et al (2001), Grimminger et al (2018), Page et al (2005), Plum et al (2018), Shuto et al (2017), Ubels et al (2023), Turkyilmaz et al (2009), Xu et al (2016), Yin et al (2012), Yin et al (2021), Zhang et al (2017). |
| Delphi statements *(% consensus)*:   - A nasogastric drainage tube is a useful drainage tool in patients with an intrathoracic leak *(76% consensus).* - A nasogastric drainage tube is a useful drainage tool in patients with a cervical leak *(62% consensus).* | | |
| Additional remarks/considerations from the expert panel:   - A nasogastric drainage tube should be considered for the initial management of a leak to drain and decompress the gastric conduit. The panel believes that it is a safe and widely available modality to effectively stop ongoing contamination. However, it may not always be necessary, particularly in cases of a small leak. Additionally, the nasogastric tube can be removed over time in favor of alternative treatments, such as EVT or stenting. | | |

| **Recommendation 15** | | |
| --- | --- | --- |
| For patients with a confirmed leak without fluid collections, and only mild clinical signs, non-invasive management with supportive care may be considered as primary treatment. | | |
| Level of evidence | Strength of recommendation | References |
| C | IIb | Al-issa et al (2014), Birla et al (2019), Chevallay et al (2020), Fabbi et al (2021), Famiglietti et al (2020), Grimminger et al (2018), Hünerbein et al (2004), Hummel et al (2017), Jones et al (2015), Low et al (2011), Ma et al (2019), Mardin et al (2012), Ubels et al (2023), Verstegen (2019). |
| Delphi statements *(% consensus)*:   - Only non-invasive management/supportive care should be performed in patients with a cervical leak with only local manifestations without fluid collections and mild symptoms *(48% consensus)*. - Only non-invasive management/supportive care should be performed in patients with an intrathoracic leak with only local manifestations without fluid collections and mild symptoms *(44% consensus)*. | | |
| Additional remarks/considerations from the expert panel:   - Non-invasive treatment with supportive care alone may be appropriate for patients with a confirmed leak who have no fluid collections and only mild clinical signs. Although the experts believe that early endoscopic treatment may also be considered, as it may prevent patient deterioration and the formation of mediastinal fluid collections. | | |

| **Recommendation 16** | | |
| --- | --- | --- |
| For leak patients with mediastinal and/or pleural fluid collections, drainage should be performed in addition to supportive care. | | |
| Level of evidence | Strength of recommendation | References |
| C | I | Birla et al (2019), Chevallay et al (2020), Fabbi et al (2021), Griffin et al (2001), Griffiths et al (2022), Grimminger et al (2018), Famiglietti et al (2020), Jiang et al (2011), Jones et al (2015), Kauer et al (2008), Mardin et al (2012), Page et al (2005), Plum et al (2018), Shuto et al (2017), Turkyilmaz et al (2009), Ubels et al (2023), Xu et al (2016), Yin et al (2012), Yin et al (2021), Zhang et al (2017). |
| Delphi statements *(% consensus)*:   - Additional drainage interventions should be performed in patients with a cervical leak and mediastinal fluid collections *(91% consensus).* - Additional drainage interventions should be performed in patients with a cervical leak and pleural fluid collections *(92% consensus).* - Additional drainage interventions should be performed in patients with an intrathoracic leak and mediastinal fluid collections *(92% consensus).* - Additional drainage interventions should be performed in patients with an intrathoracic leak and pleural fluid collections *(96% consensus).* | | |
| Additional remarks/considerations from the expert panel:   - The expert panel believes that drainage is the most important treatment for a leak. | | |

| **Recommendation 17** | | |
| --- | --- | --- |
| Drainage modalities to consider for mediastinal fluid collections are endoscopic vacuum therapy (EVT), naso-mediastinal tube drainage, and/or radiological tube drainage. For pleural fluid collections, radiological tube drainage and/or surgical tube drainage should be considered. For a cervical leak, bedside opening of the cervical incision should be considered as a drainage modality. | | |
| Level of evidence | Strength of recommendation | References |
| C | I | Bhasin et al (2000), Birla et al (2019), Chevallay et al (2020), Chon et al (2022), El-Sourani et al (2022), El-Sourani et al (2022), Fabbi et al (2021), Famiglietti et al (2020), Griffin et al (2001), Griffiths et al (2022), Grimminger et al (2018), Hua et al (2022), Jones et al (2012), Kauer et al (2008), LeBedis et al (2013), Lee et al (2020), Mardin et al (2012), Min et al (2019), Page et al (2005), Plum et al (2018), Shuto et al (2017), Tavares et al (2021), Turkyilmaz et al (2009), Ubels et al (2023), Verstegen et al (2019), Wichmann et al (2022), Xu et al (2016), Yin et al (2021), Zhang et al (2021). |
| Delphi statements *(% consensus)*:   - A radiologic drainage tube is a useful drainage tool in patients with an intrathoracic leak *(81% consensus)*. - Endoscopic vacuum therapy (EVT) is a useful drainage tool in patients with an intrathoracic leak *(82% consensus).* - A surgical drainage tube is a useful drainage tool in patients with an intrathoracic leak *(79% consensus).* - Bedside opening of the cervical incision is a useful drainage tool in patients with a cervical leak *(81% consensus).* | | |
| Additional remarks/considerations from the expert panel:   - The panel believes there are several options for draining fluid collections caused by AL, depending on the availability of these modalities and the location of the collections. - Endoscopic vacuum therapy has the advantage of serving as both a drainage and leak closure tool, but it might not be available in some centers. - External drainage is preferably performed through image-guided percutaneous drainage. However, surgically placed tubes are a viable option if radiological guidance is unavailable. - Take into account the postoperative timeframe for bedside opening of the cervical incision, as it might be more difficult if the surgery occurred more than a week ago. | | |

| **Recommendation 18** | | |
| --- | --- | --- |
| For leak patients with signs of uncontrolled sepsis and/or failure of primary treatment, surgical washout and drainage should be performed. | | |
| Level of evidence | Strength of recommendation | References |
| C | I | Birla et al (2019), Chevallay et al (2020), Fabbi et al (2021), Griffiths et al (2022), Griffin et al (2001), Hummel et al (2017), Jones et al (2012), Min et al (2019), Page et al (2005), Turkyilmaz et al (2009). |
| Delphi statements *(% consensus)*:   - Signs of uncontrolled sepsis is a reason for surgical washout and better drainage *(84% consensus).* - Failure of initiated treatment is a reason for surgical washout and better drainage *(82% consensus).* | | |

| **Recommendation 19** | | |
| --- | --- | --- |
| For patients with an intrathoracic or cervical leak with mediastinal and/or pleural fluid collections, endoscopic leak closure interventions can be performed in addition to drainage and supportive care. | | |
| Level of evidence | Strength of recommendation | References |
| C | IIa | Al-issa et al (2015), Birla et al (2019), Chevallay et al (2020), Chon et al (2022), El-Sourani et al (2022), Fabbi et al (2021), Famiglietti et al (2020), Grimminger et al (2018), Hua et al (2011), Hünerbein et al (2004), Jones et al (2015), Kauer et al (2008), Low et al (2011), Ma et al (2018), Lee et al (2020), Mardin et al (2012), Min et al (2019), Plum et al (2018), Ruiz de Angelo et al (2018), Schweigert et al (2014), Smith et al (2020), Tavares et al (2021), Turkyilmaz et al (2009), Ubels et al (2023), Verstegen et al (2019), Wichmann et al (2022), Zhang et al (2021). |
| Delphi statements *(% consensus)*:   - Additional leak closure interventions should be performed in patients with an intrathoracic leak without conduit ischemia when the patient has mild symptoms and mediastinal fluid collections *(67% consensus).* - Additional leak closure interventions should be performed in patients with an intrathoracic leak without conduit ischemia when the patient has mild symptoms and pleural fluid collections *(77% consensus).* - Additional leak closure interventions should be performed in patients with an intrathoracic leak without conduit ischemia when the patient is septic and has mediastinal fluid collections *(83% consensus).* - Additional leak closure interventions should be performed in patients with an intrathoracic leak without conduit ischemia when the patient is septic and has pleural fluid collections *(83% consensus).* - Additional leak closure interventions should be performed in patients with a cervical leak without conduit ischemia when the patient has mild symptoms and mediastinal fluid collections *(52% consensus).* - Additional leak closure interventions should be performed in patients with a cervical leak without conduit ischemia when the patient has mild symptoms and pleural fluid collections *(56% consensus).* - Additional leak closure interventions should be performed in patients with a cervical leak without conduit ischemia when the patient is septic and has mediastinal fluid collections *(70% consensus).* - Additional leak closure interventions should be performed in patients with a cervical leak without conduit ischemia when the patient is septic and has pleural fluid collections *(69% consensus).* | | |
| Additional remarks/considerations from the expert panel:   - The expert panel believes that drainage is the most important treatment for a leak. However, managing sepsis by draining the collections and concurrently, or at a later stage, resolving the leak through endoscopic closure interventions can also be considered. - The rationale for considering leak closure interventions in septic patients is that it provides source control, which is a general principle in the treatment of sepsis. Adequate drainage may allow the leak to heal without closure interventions. However, performing leak closure interventions can prevent sepsis due to inadequate source control. - If a leak requires pleural fluid drainage, it is likely too large to close with drainage alone. Therefore, additional leak closure interventions can be considered. - Some experts believe that leak closure interventions are unnecessary for a cervical leak and that adequate drainage through opening the cervical incision is sufficient. However, if the leak extends into the thoracic cavity, interventions such as EVT may still be necessary. - When considering leak closure interventions for cervical anastomoses, reduced patient tolerance of stents and EVT should be taken into account. | | |

| **Recommendation 20** | | |
| --- | --- | --- |
| If a leak closure intervention is performed in patients with an intrathoracic leak, endoscopic vacuum therapy (EVT), stent, and/or endoscopic vacuum therapy combined with stent should be considered as useful closure modalities. | | |
| Level of evidence | Strength of recommendation | References |
| C | I | Al-Issa et al (2015), Birla et al (2019), Chevallay et al (2020), Chon et al (2022), El-Sourani et al (2022), Fabbi et al (2021), Famiglietti et al (2020), Grimminger et al (2018), Hua et al (2011), Hünerbein et al (2004), Jones et al (2015), Kauer et al (2008), Low et al (2011), Ma et al (2018), Lee et al (2020), Mardin et al (2012), Min et al (2019), Plum et al (2018), Ruiz de Angelo et al (2018), Schweigert et al (2014), Smith et al (2020), Tavares et al (2021), Turkyilmaz et al (2009), Ubels et al (2023), Verstegen et al (2019), Wichmann et al (2022), Zhang et al (2021). |
| Delphi statements *(% consensus)*:   - EVT is a useful leak closure intervention in patients with an intrathoracic leak *(82% consensus).* - VACStent is a useful leak closure intervention in patients with an intrathoracic leak *(77% consensus).* - Stent placement is a useful leak closure interventions in patients with an intrathoracic leak *(71% consensus).* | | |
| Additional remarks/considerations from the expert panel:   - In recent years, there appears to be an increasing trend of choosing EVT over stents, with good results. However, there is still no randomized data published due to the novelty of the procedure, but several trials are ongoing. - Also, due to their novelty, these treatment options are not available in all centers, especially the combination of EVT and stent. It may be worth considering referring patients with an AL to a facility where these options are available. | | |

| **Recommendation 21** | | |
| --- | --- | --- |
| If surgical washout and drainage is performed in patients, additional surgical closure of the defect using sutures or tissue may be considered. | | |
| Level of evidence | Strength of recommendation | References |
| C | IIb | Al-issa et al (2014), Birla et al (2019), Chevallay et al (2020), Fabbi et al (2021), Famiglietti et al (2020), Grimminger et al (2018), Hünerbein et al (2004), Hummel et al (2017), Jones et al (2015), Low et al (2011), Ma et al (2019), Mardin et al (2012), Ubels et al (2023), Verstegen (2019). |
| Delphi statements *(% consensus)*:   - Signs of uncontrolled sepsis are a reason for surgical repair of the anastomotic defect *(29% consensus).* - Step-up after failure of initiated treatment is a reason for surgical repair of the anastomotic defect *(35% consensus).* - Early leakage (within the first 72 hours after esophagectomy) is a reason for surgical repair of the anastomotic defect *(50% consensus).* | | |
| Additional remarks/considerations from the expert panel:   - If you have a severe leak with uncontrolled sepsis and failure of less invasive treatment, where surgical washout and drainage is performed, you may consider additional surgical closure if the defect has not already been addressed using endoscopic treatment. - Indications to consider for additional closure include unstable patients with very early leaks, mostly seen in technical failures, and small leaks that are easily reachable during surgery. - In the case of a late leak with an insufficient anastomosis, surgical suturing of the defect is not recommended. | | |

| **Recommendation 22** | | |
| --- | --- | --- |
| For non-septic leak patients with limited ischemia/necrosis of the top of the gastric conduit, continuity-preserving treatment should be considered as primary treatment. | | |
| Level of evidence | Strength of recommendation | References |
| C | I | Birla et al (2019), Fabbi et al (2021), Griffin et al (2001), Griffiths et al (2022), Grimminger et al (2018), Hummel et al (2017), Low et al (2011), Mardin et al (2012), Page et al (2005), Shuto et al (2017), Ubels et al (2023). |
| Delphi statements *(% consensus)*:   - Continuity-preserving treatment should be the first-choice treatment in leak patients with mild symptoms and limited ischemia/necrosis of the top *(84% consensus).* | | |
| Additional remarks/considerations from the expert panel:   - There is experience using endoscopic treatment to successfully preserve a gastric conduit, even when circumferential necrosis of approximately 3 cm is present. Therefore, continuity preservation should be attempted in patients with mild symptoms, rather than opting for primary esophageal diversion. | | |

| **Recommendation 23** | | |
| --- | --- | --- |
| For leak patients with substantial ischemia/necrosis of the gastric conduit, primary esophageal diversion should be performed. | | |
| Level of evidence | Strength of recommendation | References |
| C | I | Birla et al (2019), Chevallay et al (2020), Fabbi et al (2021), Griffin et al (2001), Griffiths et al (2022), Grimminger et al (2018), Hummel et al (2017), Low et al (2011), Mardin et al (2012), Page et al (2005), Turkyilmaz et al (2009), Ubels et al (2023). |
| Delphi statements *(% consensus)*:   - Primary esophageal diversion should be the first-choice treatment for septic leak patients and overall ischemia/necrosis of the conduit *(89% consensus).* - Primary esophageal diversion should be the first-choice treatment in leak patients with mild symptoms and overall ischemia/necrosis of the conduit *(75% consensus).* | | |
